# Changes in tropospheric air quality related to the protection of stratospheric ozone in a changing climate

**DOI:** 10.1007/s43630-023-00369-6

**Published:** 2023-06-13

**Authors:** S. Madronich, B. Sulzberger, J. D. Longstreth, T. Schikowski, M. P. Sulbæk Andersen, K. R. Solomon, S. R. Wilson

**Affiliations:** 1 National Center for Atmospheric Research, Boulder, USA; 2 USDA UV-B Monitoring and Research Program, Natural Resource Ecology Laboratory, Colorado State University, Fort Collins, USA; 3 Academic Guest after retirement from Eawag: Swiss Federal Institute of Aquatic Science and Technology, CH-8600 Duebendorf, Switzerland; 4 The Institute for Global Risk Research, LLC, Bethesda, USA; 5 IUF-Leibniz Research Institute for Environmental Medicine, Dusseldorf, Germany; 6 Department of Chemistry and Biochemistry, California State University, Northridge, USA; 7 School of Environmental Sciences, University of Guelph, Guelph, Canada; 8 School of Earth, Atmospheric and Life Sciences, University of Wollongong, Wollongong, Australia

## Abstract

Ultraviolet (UV) radiation drives the net production of tropospheric ozone (O_3_) and a large fraction of particulate matter (PM) including sulfate, nitrate, and secondary organic aerosols. Ground-level O_3_ and PM are detrimental to human health, leading to several million premature deaths per year globally, and have adverse effects on plants and the yields of crops. The Montreal Protocol has prevented large increases in UV radiation that would have had major impacts on air quality. Future scenarios in which stratospheric O_3_ returns to 1980 values or even exceeds them (the so-called super-recovery) will tend to ameliorate urban ground-level O_3_ slightly but worsen it in rural areas. Furthermore, recovery of stratospheric O_3_ is expected to increase the amount of O_3_ transported into the troposphere by meteorological processes that are sensitive to climate change. UV radiation also generates hydroxyl radicals (OH) that control the amounts of many environmentally important chemicals in the atmosphere including some greenhouse gases, e.g., methane (CH_4_), and some short-lived ozone-depleting substances (ODSs). Recent modeling studies have shown that the increases in UV radiation associated with the depletion of stratospheric ozone over 1980–2020 have contributed a small increase (~ 3%) to the globally averaged concentrations of OH. Replacements for ODSs include chemicals that react with OH radicals, hence preventing the transport of these chemicals to the stratosphere. Some of these chemicals, e.g., hydrofluorocarbons that are currently being phased out, and hydrofluoroolefins now used increasingly, decompose into products whose fate in the environment warrants further investigation. One such product, trifluoroacetic acid (TFA), has no obvious pathway of degradation and might accumulate in some water bodies, but is unlikely to cause adverse effects out to 2100.

**Graphical abstract:**

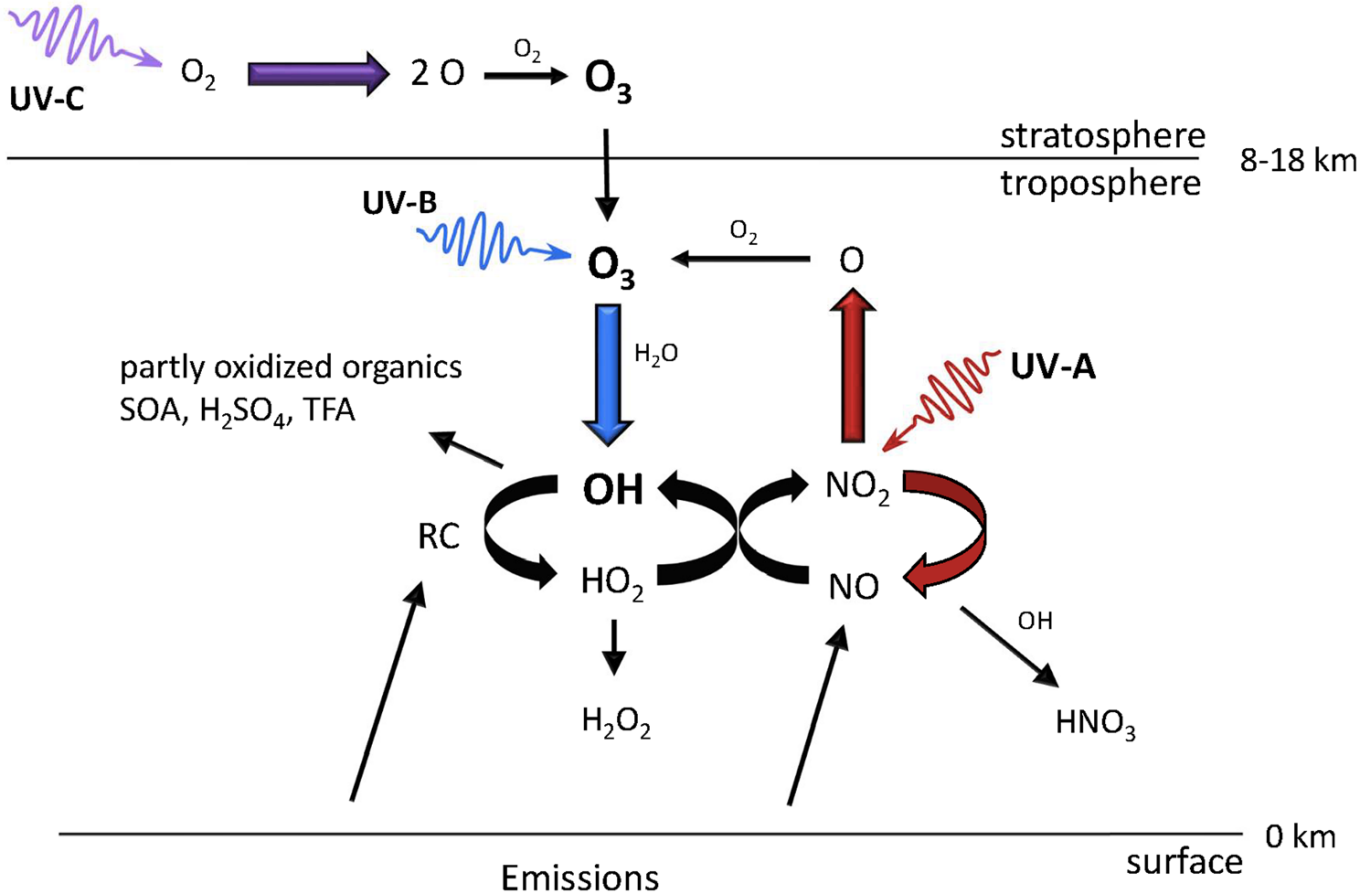

**Supplementary Information:**

The online version contains supplementary material available at 10.1007/s43630-023-00369-6.

## Introduction

The protection of stratospheric ozone (O_3_) has had important consequences for the chemical composition of the lower atmosphere (the troposphere) and the quality of the air that humans and many other organisms breathe. Ultraviolet (UV) radiation plays an essential role in the generation of photochemical smog, exposure to which is associated with widespread health effects, reductions in life expectancies, damage to forests, and smaller agricultural yields. Given the number of populations and ecosystems currently affected by photochemical smog, even small changes in UV radiation are important, such as those associated with the few percent depletion of stratospheric O_3_ that occurred at mid-latitudes over 1980–2000. By limiting the depletion of stratospheric O_3_, the Montreal Protocol has avoided large increases in tropospheric UV radiation that would have exacerbated photochemical air pollution in urban areas.

On the global scale, UV-B radiation (280–315 nm) controls the self-cleaning capacity of the troposphere by generating hydroxyl radicals (OH). These radicals react with many chemicals emitted to the troposphere, including greenhouse gases such as methane, facilitating their removal from the atmosphere and essentially determining their atmospheric lifetime. The Montreal Protocol has maintained the troposphere’s self-cleaning capacity at near natural levels, but future changes remain a concern, especially if the intensity of UV-B radiation decreases substantially due to increasing stratospheric ozone under some future scenarios.

Actions under the Montreal Protocol have led to the introduction of new chemicals to the atmosphere as replacements to some of the ozone-depleting substances, including hydrofluorochlorocarbons (HCFCs), hydrofluorocarbons (HFCs), hydrofluoroethers (HFEs), hydrofluoroolefins (HFOs) and hydrochlorofluoroolefins (HCFOs). However, their atmospheric photo-degradation can lead to persistent secondary pollutants such as trifluoroacetic acid (TFA), whose ultimate fate in the environment remains unclear, requiring continued monitoring and assessment relative to other natural and/or anthropogenic sources.

We have reported on these issues in our previous assessments [1–6] and our objective here is to provide an updated overview and assessment of the scientific evidence. As will be presented in the following sections, our previous conclusions remain qualitatively unchanged and consistent with increasingly available observations and numerical simulations. This assessment consists of two parts: The first part (Sect. 2) focuses on the effects of depletion of stratospheric ozone, solar UV radiation (particularly UV-B), and interactions with climate change on tropospheric air quality and how changes in tropospheric air quality affect human health and ecosystems. The second part (Sect. 3) assesses the known sources of trifluoroacetic acid (TFA), including those related to the replacement chemicals under the purview of the Montreal Protocol, and their potential risk to humans and ecosystems.

In conducting this assessment, we have searched the literature through PubMed^®^, Google Scholar, ScienceDirect®, and relevant journals to obtain peer-reviewed papers from the recent literature (2018–2022) as well as reports from recognized international agencies (e.g., World Health Organization) and government agencies (e.g., US Environmental Protection Agency). We have critically evaluated these papers and reports before including information from them in this Quadrennial Assessment.

## UV-dependent air pollutants and their effects on human health, plants, and the self-cleaning capacity of the troposphere

The importance of UV radiation to the formation of some types of air pollution has been known at least since the studies of photochemical smog in Los Angeles in the 1950s, when it was shown that ambient O_3_ was generated by UV-induced reactions involving nitrogen oxides (NOx) and volatile organic compounds (VOCs) [7]. Since then, many details of these photochemical reactions have been elucidated, such as the central role of UV-generated OH radicals in controlling the overall reactivity; and the formation of O_3_, peroxides, acids, and other harmful gaseous intermediates, including some that can condense to form particulate matter (PM). This gas- and condensed-phase chemistry is complex, involving hundreds of different chemicals, often with rapidly changing emissions and different environmental conditions.

A brief overview/summary of tropospheric chemistry, with emphasis on the distinct roles of UV-B and UV-A radiation, is provided in Sect. 2.1. The formation of photochemical smog, specifically its ground-level O_3_ and UV-sensitive PM components, is discussed in Sects. 2.2 and 2.3, including an assessment of the possible effects of changes in UV radiation related to the recovery of stratospheric ozone over the coming decades. Exposure to photochemical smog can have large impacts on human health, particularly in vulnerable populations even at low concentrations of pollutants (Sect. 2.4). Tropospheric O_3_ and PM can also affect plant health (Sect. 2.5). UV-B radiation has beneficial effects by causing the formation of OH, the cleaning agent of the troposphere (Sect. 2.6). Finally, changes in atmospheric circulation and the transport of pollutants affect the tropospheric air quality (Sect. 2.7). A summary of our assessment is provided in Sect. 2.8.

### Background: the UV photochemistry of tropospheric air

**Fig. 1:**
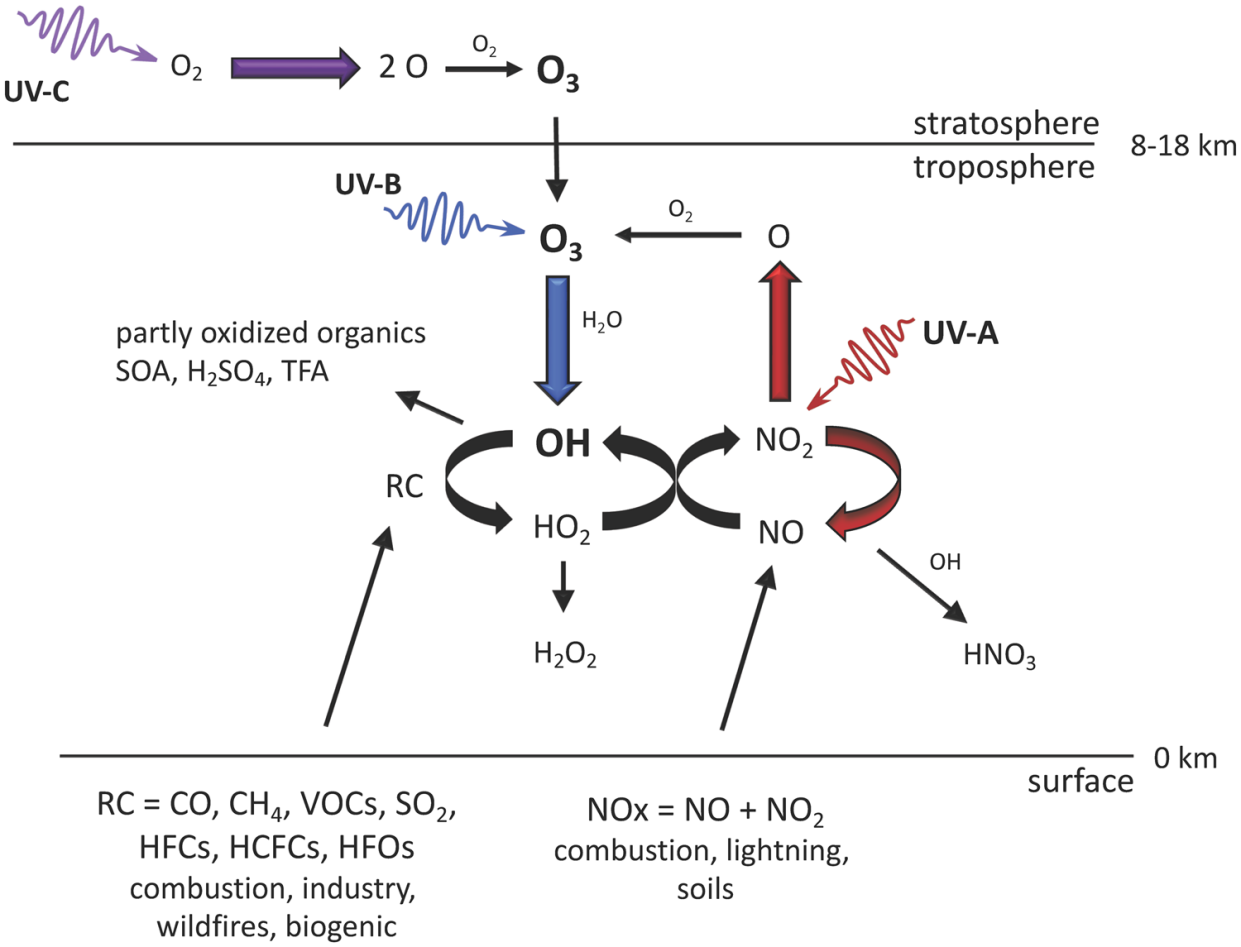
Simplified schematic of tropospheric photochemistry. UV-C radiation (100–280 nm) in the stratosphere generates ozone, O_3_, some of which is transported to the troposphere. UV-B radiation initiates tropospheric chemistry by photo-dissociating O_3_ and generating highly reactive hydroxyl radicals (OH). These react with many compounds emitted by human activities and natural processes, e.g., carbon monoxide, methane, volatile organic compounds including halocarbons, and others, all generalized in the figure as RC (reduced compounds). By removing these compounds, the concentration of OH controls the self-cleaning capacity of the atmosphere. Nitrogen oxides (NO_*x*_ = NO + NO_2_) catalyze the photo-oxidation by regenerating OH via reaction of NO with HO_2_. This coupling of the NO_*x*_ and HO_*x*_ (OH + HO_2_) cycles also leads to autocatalytic production of O_3_, often in amounts larger than lost initially via its UV-B photolysis, since NO_*x*_ and HO_*x*_ molecules can cycle many times before being removed. The cycles are terminated by reaction with OH to make nitric acid, (HNO_3_) or by reaction of HO_2_ to inorganic or organic peroxides, e.g., hydrogen peroxide (H_2_O_2_). Other products, depending on the reduced compounds being oxidized by OH, could include partly oxidized organics, secondary organic aerosols (SOA), sulfuric acid (H_2_SO_4_), and trifluoroacetic acid (TFA)

The most important UV-induced processes that control air quality in the troposphere are shown in Fig. 1. UV-B radiation is responsible for the formation of the OH radical, the major oxidizing agent in the troposphere. This occurs through the photolysis of O_3_ and subsequent reaction of an electronically excited oxygen atom with water (H_2_O). Hydroxyl radicals are lost by reaction with reduced chemicals, including carbon monoxide (CO), methane (CH_4_), VOCs, sulfur dioxide (SO_2_), and nitrogen dioxide (NO_2_) (Fig. 1). Hydroxyl radicals control the atmospheric amounts of these chemicals as well as those of many other important trace gases, e.g., HFCs, HCFCs, HFOs, and very-short-lived substances (VSLSs, e.g., halo-organics with a lifetime of less than or equal to 6 months). Chemicals such as chlorofluorocarbons (CFCs) that do not react with OH have the potential to reach the stratosphere in large amounts; the CFCs were, therefore, replaced with chemicals that react with OH (HCFCs, HFCs, HFOs, etc.).

UV-B and UV-A radiation are together responsible for the net production of O_3_ in the troposphere. This occurs via photo-dissociation of nitrogen dioxide (NO_2_) to NO and O, mainly by UV-A radiation, and subsequent reaction of the oxygen atom with molecular oxygen (Fig. 1). Nitrogen oxides (NO_*x*_ = NO + NO_2_) are emitted primarily as NO, and the NO_2_ is produced via the re-cycling of the hydroxyl radical (OH) generated from the UV-B photolysis of O_3._ Due to this autocatalytic production of O_3_ involving OH, the net production of O_3_ depends not only on UV-A radiation but also on UV-B radiation. The different effects of UV-B and UV-A radiation are discussed in more detail in Box 1. Note that Fig. 1 is restricted to reactions occurring in the gas phase, while heterogeneous, UV-induced processes involving aerosols are discussed in Sect. 2.3.

**Figure:**
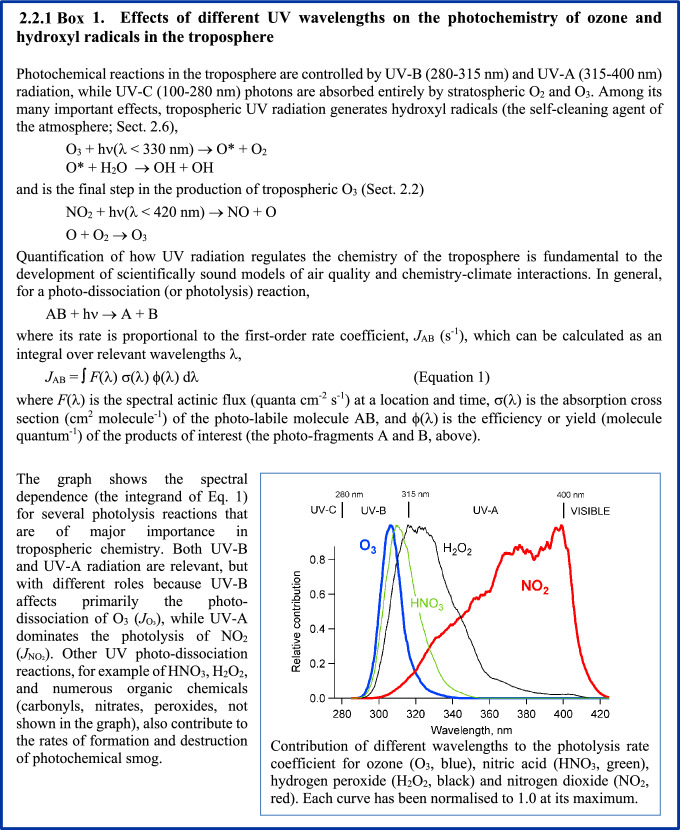

### UV radiation and ground-level ozone

**Fig. 2:**
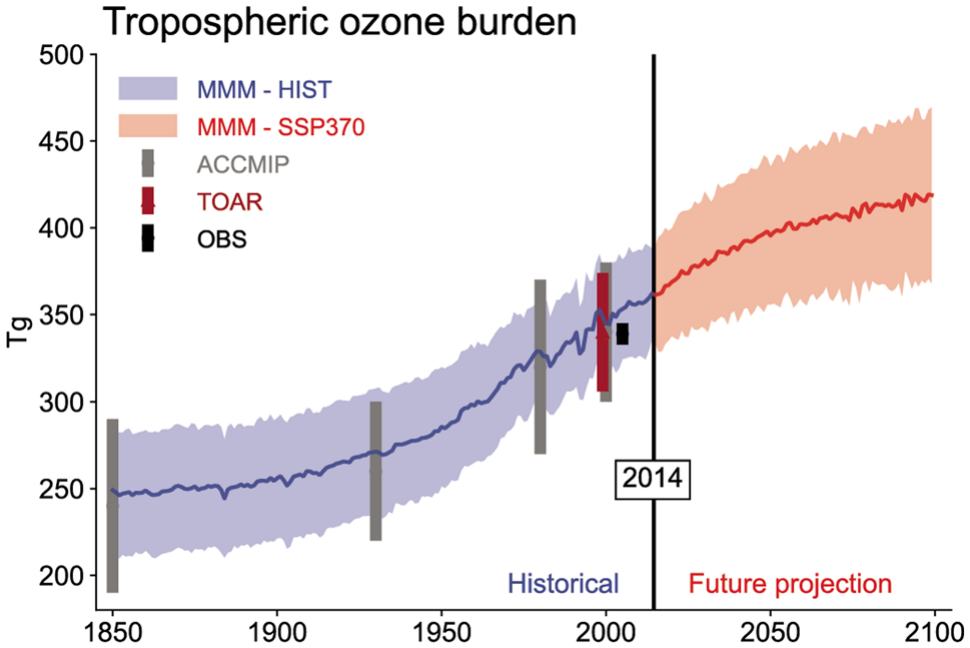
Global tropospheric ozone (Tg) estimated by multi-model assessments (MMM, ACCMIP, TOAR) and observations (OBS, for the year 2000). Future projections are for one Shared Socioeconomic Pathway (SSP370 scenario). From IPCC 2021 Ch. 6 [11]

Ground-level O_3_ continues to be a major environmental problem, with costly impacts on human health and vegetation. Most ground-level O_3_ is produced by the UV photochemical processing of pollutants (see Sect. 2.1), with occasional contributions from downward transport of ozone-rich stratospheric air (see Sect. 2.7). It is generally accepted that concentrations of O_3_ have increased throughout the global troposphere over the past century, due to increasing emissions of VOCs and NOx. This is borne out in numerical simulations with chemistry-climate models, as shown in Fig. 2. Contributions from stratospheric ozone depletion (1980 to current) and the associated increases in UV-B radiation have been negligible by comparison, at least for the global scale. Future projections tend to show an increasing global burden of tropospheric O_3_, although the details depend on the assumed scenario of greenhouse gas emissions (only one shown in the figure, SSP370).

**Fig. 3:**
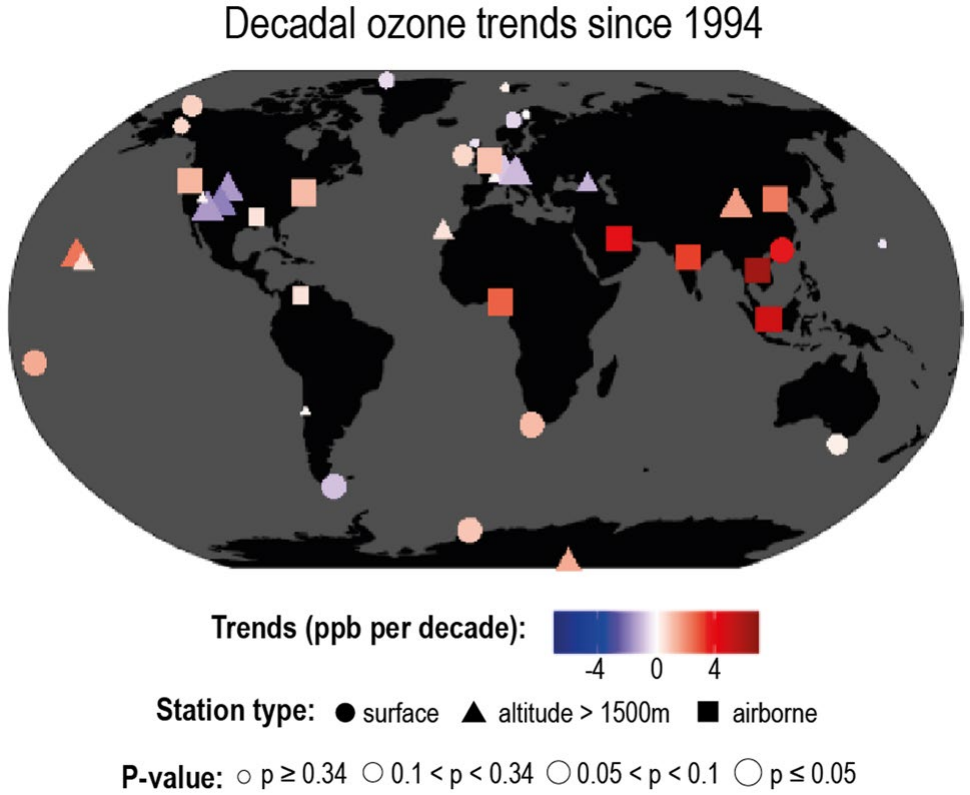
Regional and local trends in tropospheric ozone at the surface and lower atmosphere. From IPCC 2021 Ch. 6 [11]

Trends in local and regional concentrations of tropospheric O_3_ vary greatly as shown in Fig. 3. Over the past few decades, concentrations of O_3_ at ground-level and in the lower atmosphere have been generally decreasing in most developed countries, while increasing greatly in some locations including East and South Asia, in response to changes in emissions of NOx and VOCs. Reductions in emissions of NOx and VOCs will be necessary to reverse the observed increasing trends, but any substantial future changes in UV radiation could modify the effectiveness of such reductions.

The possibility that ground-level O_3_ can be affected by changes in UV related to depletion of stratospheric ozone was first pointed out by Liu and Trainer [12] and has since been confirmed by several studies using numerical models [13–17], as well as observations at the South Pole [18] and at mid-latitudes [19]. More intense UV-B irradiation generally induces faster chemical reactivity in air parcels (Box 1), leading to faster removal of primary chemicals, but also more rapid and intense build up (and removal) of intermediate or secondary pollutants, such as hetero-organics (e.g., aldehydes, ketones, and organic nitrates) and other by-products including O_3_, peroxides, and secondary PM. Conversely, reductions in UV radiation decrease chemical reactivity, causing slower production of O_3_ and other photochemical pollutants near source regions, e.g., urban areas, while slowing the destruction on regional and global scales.

The decreases in UV radiation at the surface, expected from the recovery of stratospheric O_3_, to 1980 levels are estimated to have only a small impact on ambient O_3_, as reported previously [14, 15], since reductions of O_3_ at mid-latitudes have been limited to only a few percent (relative to a 1980 baseline). For the United States, the recovery to 1980 levels will increase the O_3_ column by 4–6%, and the resulting lower levels of UV radiation will tend to lower ambient O_3_ over a few large urban areas, while raising it slightly elsewhere, consistent with an overall slower chemical reactivity. However, recent climate model simulations [20–22], also reviewed by Bernhard et al. [23], suggest that under some scenarios of increasing greenhouse gas emissions, stratospheric O_3_ could exceed the 1980 baseline (the so-called “super-recovery”). By 2100, stratospheric O_3_ could increase by an additional 10% (above the 1980 levels) under high emission scenarios (SSP3-7.0, SSP4-6.0 and SSP5-8.5; see Fig. 3 of Bernhard et al. [23]). This would decrease tropospheric *J*_O3_ by about 14% (Box 2), like the changes used in the sensitivity calculation shown in Box 3. Under these scenarios, the changes in tropospheric O_3_ (urban declines and regional increases) would be about three times greater than those estimated for recovery limited to 1980 levels [14, 15]. Even at current levels (see Figs. 2 and 3), ground-level O_3_ damages vegetation and causes economically significant reductions in crop yields (see Sects. 2.4 and 2.5). Additional increases due to the recovery (or super-recovery) of stratospheric O_3_ are of concern but could be offset by more stringent reductions in emissions of NOx and VOCs.

Much larger changes would have occurred without the implementation of the Montreal Protocol (the “world avoided”), where unabated growth of emissions of CFCs would have resulted in catastrophic global loss of stratospheric O_3_ [24], with major impacts on UV radiation [25], incidence of skin cancer [26, 27], and reduction of the global carbon sink by damage to vegetation [28]. However, to our knowledge, calculations of the impacts of such large increases in UV radiation on air quality, particularly ambient O_3_ and secondary aerosols, have not been carried out.

While large changes in UV-B radiation have been avoided by the Montreal Protocol [23], trends and variability in UV radiation exist also for other reasons (especially due to aerosols and clouds), and these changes provide ongoing opportunities to better understand and quantify the representation of UV-driven chemical processes in models for air quality. Reduced emissions have systematically improved air quality in many locations, but this has increased UV radiation near the surface, potentially negating some of the benefits of the reduced emissions. For example, aerosol haze in China was reduced substantially during the last decade due to lower emissions of NOx and SO_2_. This has led to surface brightening (e.g., by 0.70–1.16 W m^−2^ year^−1^ in eastern China over 2014–2019) [29] but also to undesirable increases in ambient O_3_ [30–33] (e.g., by 2–6 µg m^−3^ year^−1^ in megacity clusters of Beijing and Shanghai over 2013–2017) [34]. Simulations with numerical models show several possible reasons including increases in UV radiation [31, 33–39], shifts in the VOC/NOx chemical regime resulting in increased production of O_3_ efficiency [35, 40], higher emissions of biogenic VOC due to rising temperatures [30], and decreased competition for gas-phase radicals by aerosol surfaces [34, 40], with reality likely being a combination of these factors.

Long-term increases in UV radiation at the surface due to improved local air quality have been recorded in many other locations. For example, increasing trends in UV radiation over 1996–2016 were found [41] for stations in Japan and Greece, and were attributed to reductions in absorbing aerosols. More recently, Ipiña et al. [42] found that the UV Index in Mexico City increased by ca. 20% over 2000–2019 (due to reductions in PM, SO_2_, O_3_, and NO_2_) and estimated that such increases in UV radiation would require an additional 10% reduction in VOC emissions to meet the same ground-level O_3_ concentrations had the UV remained constant. Brief increases in UV radiation at the surface have been observed during the economic slowdowns related to the COVID-19 pandemic, e.g., in Brazil [43], East Asia [44], India [45], and likely in many other locations. Analysis of these data is ongoing and should yield insights on how atmospheric pollution responds to changes in UV radiation under different conditions.

**Figure:**
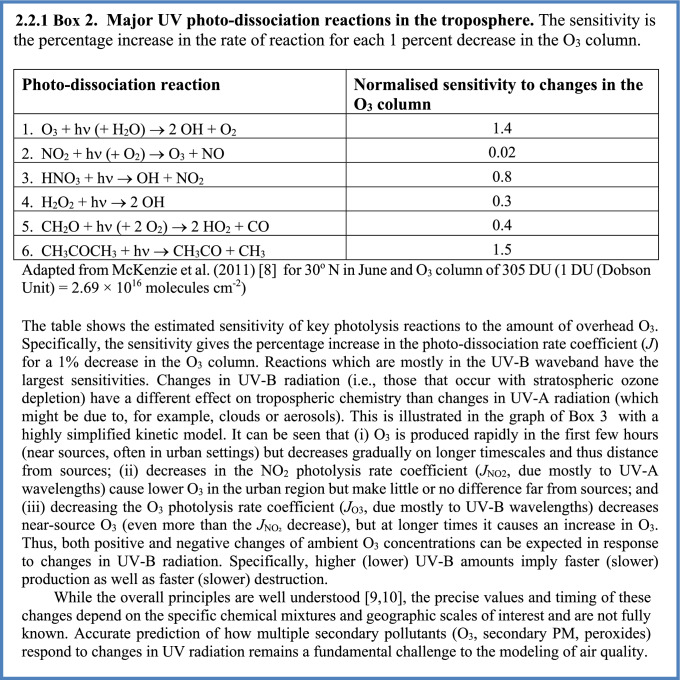

**Figure:**
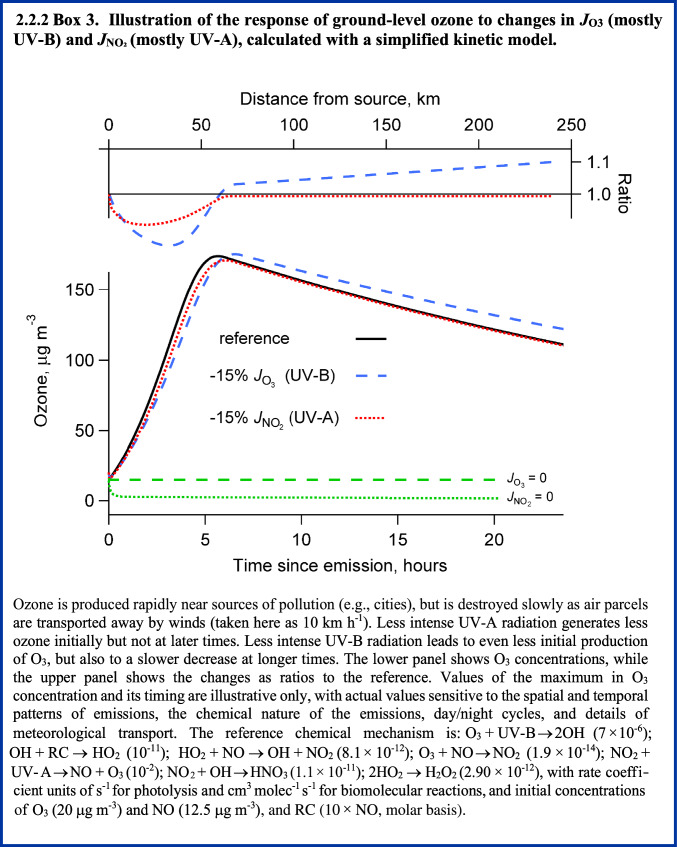

### UV radiation and particulate matter

Particulate matter is a major component of air pollution, and particles smaller than about 2.5 µm (PM_2.5_) are believed to be particularly damaging due to their ability to penetrate deeply into lungs. A large fraction of PM_2.5_ is formed by UV-initiated photochemistry. While “primary” PM is emitted directly (e.g., dust, black carbon, or sea spray) “secondary” PM is produced in the atmosphere, typically by condensation of gases having low saturation vapor pressures. These condensable gases are mostly produced by reactions of OH with pollutants such as SO_2_, NO_2_, or VOCs, to yield sulfate, nitrate, or secondary organic aerosols (SOA), respectively:

$$\begin{gathered} {\text{OH }} + {\text{ NO}}_{{2}} \to {\text{HNO}}_{{3}} , \hfill \\ {\text{OH }} + {\text{ SO}}_{{2}} \to \ldots \to {\text{H}}_{{2}} {\text{SO}}_{{4}} , \hfill \\ {\text{OH }} + {\text{ VOC}} \to \ldots \to {\text{ }}\left( {\text{various partly oxidised VOCs}} \right) \to {\text{SOA,}} \hfill \\ \end{gathered}$$

where the last two reactions involve several intermediate steps. The rate-limiting step in the production of these particles is the reaction of OH with the precursors (NO_2_, SO_2_, or VOCs), so that the dependence of OH on UV radiation (see Sect. 2.1) applies directly to the rate of formation of secondary PM as well. Decreases (increases) in stratospheric O_3_ lead to increases (decreases) in tropospheric UV-B radiation and concentrations of OH radicals, and therefore to faster (slower) formation of these PM. The Montreal Protocol, through its influence on the amount of UV radiation reaching the troposphere, has direct consequences for the formation of secondary PM. Primary PM, on the other hand, is not expected to depend strongly on UV irradiation.

**Fig. 4:**
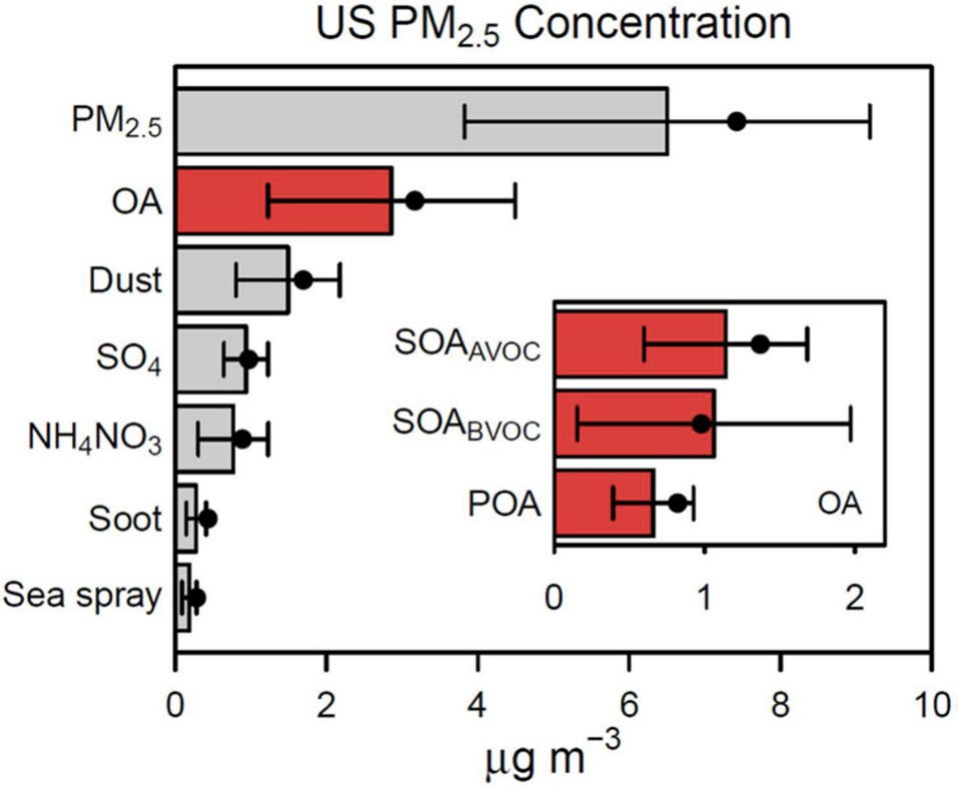
Composition of PM_2.5_ over the contiguous United States calculated with a chemistry-transport model. Particles produced by UV photochemical reactions (secondary aerosols) include sulfate (SO_4_), ammonium nitrate (NH_4_NO_3_) and secondary organic aerosols (SOA) from anthropogenic or biogenic precursors (SOA_AVOC_ and SOA_BVOC_, respectively), and account for more than half of the total PM_2.5_, compared to directly emitted particles (primary aerosols) such as dust, soot, and sea spray. VOC, volatile organic compounds. From Pye et al. [46]

The relative amounts of primary and secondary PM vary greatly in time and space, and estimates exist only for regions where reliable emission inventories exist. For the contiguous United States (Fig. 4) modeling studies indicate that more than half of the PM_2.5_ is secondary in origin, and thus directly sensitive to variations in UV radiation [46]. Globally, major contributors to PM_2.5_ are sulfate, nitrate, organics, ammonium, and black carbon (see Fig. 6.7 of IPCC 2021 [11]). While sulfate and nitrate PM are of secondary origin, for organics the relative global contribution of primary and secondary PM is less clear. Satellite-based observations of aerosol optical properties provide only very limited information about chemical composition [47, 48]. A better understanding of the secondary/primary ratio of PM_2.5_ in all populated regions is required to fully assess the role of UV radiation and, hence, the relevance of the Montreal Protocol to this global air pollution problem.

An emerging and rapidly evolving topic is the effect of UV radiation on chemical reactions within and on the surface of aerosol particles. These heterogeneous processes are extremely complex and still poorly understood, at least in part because aerosols may be composed of many different chemicals, often mixed within the same particle, and each with different UV-absorbing properties and different photolytic fragments. Depending on the specific particle, several UV-mediated processes have already been identified: (a) photolysis of particle-bound organic chemicals into volatile gases (e.g., CO and CO_2_), leading to loss of particle mass [49–54]; (b) photolysis of particle-bound inorganic and organic nitrogen into gaseous NO, NO_2_, or HONO, potentially increasing the oxidizing capacity of the atmosphere [55–58]; (c) conversion of SO_2_ gas on the surface of particles to particulate sulfate (thus, increasing the mass of the particle) [59–61]; (d) formation of molecular chlorine (Cl_2_) leading to enhanced reactivity of a daytime suburban atmosphere [62], (e) increased absorption of shortwave radiation (from formation of brown carbon) with consequences for radiative forcing of climate [63–67]; and (f) formation of various reactive oxygen species (ROS), some of which (e.g., peroxides) are sufficiently long-lived that they may persist during inhalation and play a major role in deleterious health effects of air pollution [61, 64, 68–72].

Ultraviolet-induced photo-processes within and on the surface of aerosols can also increase the ability of aerosols to act as cloud condensation nuclei (CCN) [73, 74], which can change cloud properties and therefore indirectly impact climate. UV-B irradiation (~ 4.5 days solar radiation equivalent) of dissolved organic matter (DOM, used as a surrogate for organic aerosols) from freshwaters resulted in an increase of hygroscopicity by up to 2.5 times [73]. This is a result of the photo-degradation of DOM into hydrophilic low molecular weight chemicals, a process that has been reported in sunlit surface waters [75, 76]. A similar effect on CCN was also observed upon UV-B irradiation of SOA formed from the oxidation of α-pinene and naphthalene [74], which are biogenic and anthropogenic SOA precursors, respectively. Considering that CCN are central to the lifecycle of clouds, this represents a newly recognized and potentially important dependence of the hydrological cycle (and climate in general) on UV radiation.

In most cases, the spectral dependence of these heterogeneous photo-processes is still unknown, so that it is not yet possible to assess reliably how much they would be influenced by changes in UV-B radiation resulting from changes in stratospheric ozone. As more spectral data become available, our understanding of the multiple ways in which UV radiation influences aerosol properties and lifetimes will increase and illuminate what role this plays in the natural and perturbed atmosphere.

In summary, changes in UV radiation affect the formation, transformation, and destruction of PM. Quantification of these effects of UV radiation is still somewhat problematic because it involves complex chemical feedbacks that depend on specific physical and chemical variables, such as temperature, humidity, and the amounts of VOCs and NOx present. However, despite this uncertainty, even small changes in UV radiation should be of concern, because of the large number of people currently exposed to poor air quality and its significance to human health.

### Health impacts of photochemical smog

Air pollution is a major public health concern. Estimates of the impacts vary but are consistently large. Thus, the World Health Organization (WHO) estimates that 4.2 million deaths every year occur because of exposure to ambient (outdoor) air pollution which includes particulates and gases, such as O_3_, NO_x_, etc. [77]. These values are somewhat higher than the values reviewed in our previous assessment, which ranged from 1.75 to 4.3 million depending on year and source of estimates [6]. Regular reports on concentrations of tropospheric O_3_ and its effects on humans and the environment are published in Tropospheric Ozone Assessment Reports, e.g., [78]. Our previous Quadrennial Assessment [6] provided an overview of the effects of air pollution on human health with much of the information focusing on respiratory and cardiovascular morbidity and mortality, although reproductive and neurological effects were also briefly addressed. Information on these and other effects from exposure to air pollution continues to accumulate.

An umbrella review (a review of systematic reviews and meta-analyses) [79] evaluated 548 meta-analyses derived from 75 systematic reviews on non-region-specific associations between outdoor air pollution and human health. Of these meta-analyses, 57% (313) were not statistically significant. Of the 235 nominally significant meta-analyses, all but 5 indicated an adverse effect on human health. Analyses were graded as strong (13), highly suggestive (23), suggestive (67) or weak (132). Strong evidence for an association between outdoor air pollution exposure and cardiorespiratory diseases was found for:

1. an increased risk of stroke-related mortality per 10 µg m^−3^ increase of PM_10_ and PM_2.5_ (short-term exposure; relative risk (RR): 1.005 95% CI 1.003–1.007 and RR: 1.014, 95% CI 1.009–1.020, respectively);
2. hypertension per 10 µg m^−3^ increase of PM_2.5_ (short-term exposure; odds ratio (OR) 1.097, 95% CI 1.060–1.136);
3. asthma-related admissions per 10 µg m^−3^ increase of PM_2.5_ and NO_2_ levels (short-term exposure; OR: 1.022, 95% CI 1.014–1.031 and OR: 1.019, 95% CI 1.1013–1.024, respectively);
4. chronic obstructive pulmonary disease (COPD) and asthma-related admissions of the elderly per 10 µg m^−3^ increase of NO_2_ (24 h average; RR: 1.386%, 95% CI 1.110–1.661%);
5. mortality due to pneumonia per 10 µg m^−3^ increase NO_2_ levels (long-term exposure; Hazard Ratio (HR): 1.077, 95% CI 1.060–1.094).

#### Health impacts at low concentrations of air pollution

New studies, assessed here, indicate that even relatively low levels of pollution may be detrimental [80–82]. Many countries have acted by regulating concentrations of key pollutants and there has been a remarkable decrease in air pollution levels in almost all countries with developed economies leading to levels below the air pollution standards. With the decrease in concentrations of key pollutants, studies now show the detrimental effects on health at relatively low levels of air pollution. Many show effects at concentrations lower than the current annual average standard. In response to this, the World Health Organization (WHO) updated its 2005 Global Air Quality Guidelines (AQG) in September 2021 [83, 84]. These new air quality guidelines [83] set ambitious goals, which will be difficult to achieve in most countries. They reflect the large impact that air pollution has on health globally. The new guidelines are aiming for annual mean concentrations of PM_2.5_ not exceeding 5 µg m^−3^ and NO_2_ not exceeding 10 µg m^−3^, and the peak season mean 8-h O_3_ concentration not exceeding 60 µg m^−3^ [83]. For comparison, the corresponding 2005 WHO guideline values for PM_2.5_ and NO_2_ were 10 µg m^−3^ and 40 µg m^−3^ with no recommendation issued for long-term concentrations of O_3_ [85]. Table 1 presents the new WHO guidelines in comparison to standards from the European Union, the EPA (USA) and China. Note that UV radiation is involved in the formation of many of these pollutants, including O_3_, NO_2_, and a large fraction of PM_10_ and PM_2.5_, including sulfate, nitrate, and secondary organic aerosols. Furthermore, UV radiation may make PM_10_ and PM_2.5_ more toxic by generating ROS (Sect. 2.3).

**Table 1:** Summary of air quality guidelines in several jurisdictions

Air pollutant	Time frame, h	WHO new	WHO old	EU	EPA USA	China–Grade 1^a^	China– Grade 2^b^
PM_2.5_ µg m^−3^	24-h	15	25		35	35	75
	annual	5	10	25	12	15	35
PM_10_ µg m^−3^	24-h	45	50	50	150	50	150
	annual	15	20	40		40	70
NO_2_ µg m^−3^	24-h	25				80	80
	annual	10	40	40	100	40	40
	1-h		200	200	190	200	200
SO_2_ µg m^−3^	24-h	40	20	125		50	150
	annual					20	60
	1-h			350	200	150	500
CO mg m^−3^	24-h	4				4	4
	1 h				40	10	10
	daily, 8-h max	10	10	10	10		
O_3_ µg m^−3^	1 h					160	200
	daily, 8-h max	100	100	120	140	100	160

*EU* European Union, *EPA* Environmental Protection Agency, PM_2.5_ particles with a diameter of 2.5 µm or less (≤ PM_2.5_), PM_10_, particles with a diameter of 10 µm or less (≤ PM_10_), NO_2_ nitrogen dioxide, SO_2_ sulfur dioxide, CO carbon monoxide, O_3_ ozone

^a^China Grade 1 Road: National Highways

^b^China Grade 2 Roads: Provincial Highway

Evidence continues to accumulate demonstrating that exposure to air pollution can have serious effects on nearly all organ systems of the human body. As outlined in our earlier assessment, the health effects of air pollution include cardiovascular and respiratory disease, cancer, effects on the brain and the reproductive system including adverse birth outcomes [6]. Much of the more recent support documenting the effects of low-level exposure has come from the study of large cohorts in Canada [80], Europe [81] and the United States [82], where regulatory efforts have reduced the average level of exposure. These studies have consistently shown that the adverse effects of air pollution are not limited to high exposures; harmful health effects can be observed at very low concentrations (see below), with no observable thresholds below which exposure can be considered safe.

Research conducted as part of the ‘Effects of Low-Level Air Pollution: A Study in Europe’ (ELAPSE) [81, 86–92] examined the mortality and morbidity effects of exposure to low concentrations of four air pollutants: PM_2.5_, NO_2_, black carbon (BC), and tropospheric warm season O_3_, with some of the research also investigating the importance of elemental components of PM_2.5_ [88, 92–94]. The ELAPSE study consisted of two sets of cohorts: The first was a pooled cohort of up to 15 conventional research cohorts, most of which were in a region with at least one large city with an associated smaller town. This resulted in a rich amount of individual data for up to 325,000 participants. The second set of cohorts comprised seven large administrative cohorts, which were formed by linking census data, population registries, and death registries. These were analyzed individually, and, in some cases, meta-analyses were conducted to produce overall results. The key strength of the administrative cohorts was their large sample size (about 28 million) and national representativeness.

The effect of low-level air pollution exposure in 22 cohorts (a combination of research and administrative cohorts) across Europe was associated with several health outcomes and mortality [81]. Almost all participants had annual average exposures below the European Union guidance values (Table 1) for PM_2.5_ and NO_2_, and about 14% had mean annual exposures below the United States National Ambient Air Quality Standards for PM_2.5_ (12 µg m^−3^). In the pooled analysis of the research cohorts, participants had been exposed to 15 µg m^−3^ PM_2.5_, 1.5 × 10^–5^ m^−1^ black carbon (BC), 25 µg m^−3^ NO_2_, and 67 µg m^−3^ O_3_ on average. Among the cohorts, mean concentrations of PM_2.5_ ranged from 12 to 19 µg m^−3^, except for the Norwegian cohort (8 µg m^−3^). The study followed 325,367 adults and found significant positive associations between even low exposure to PM_2.5_, BC, and NO_2_ and mortality from natural-causes as well as cause-specific mortality such as cardiovascular and ischemic heart disease, cerebrovascular disease, respiratory disease, COPD, diabetes, cardiometabolic disease, and lung cancer mortality [91]. An increase of 5 µg m^−3^ in PM_2.5_ was associated with 13% (95% CI 10.6–15.5%) increase in natural deaths. For participants with exposures below the United States standard of 12 µg m^−3^, an increase of 5 µg m^−3^ PM_2.5_ was associated with nearly a 30% [29.6% (95% CI 14–47.4%)] increase in natural deaths. For NO_2_, hazard ratios remained elevated and significant when analyses were restricted to observations below 20 μg m^−3^.

Liu and colleagues [95] analyzed the association between the incidence of asthma and low concentrations of air pollution using three large cohorts from Scandinavia (*n* = 98,326). They found a hazard ratio of 1.22 (95% CI 1.04–1.43) per 5 µg m^−3^ increase in PM_2.5_, 1.17 (95% CI 1.10–1.25) per 10 µg m^−3^ for NO_2_ and 1.15 (95% CI 1.08–1.23) per 0.5 × 10^–5^ m^−1^ for BC. Hazard ratios were larger in cohort subsets with exposure levels below the annual average limits for the European Union and United States (Table 1) and proposed World Health Organization guidelines for PM_2.5_ and NO_2_ (Table 1) compared to the hazard ratios in cohorts exposed to levels above the annual limits.

A meta-analysis [96] of 107 studies on the effect of long-term exposure to air pollution on mortality showed that there was strong evidence that exposure to PM_2.5_ and PM_10_ is associated with increased mortality from all causes, cardiovascular disease, respiratory disease, and lung cancer. The combined Hazard Ratios (HRs) for natural-cause mortality were 1.08 (95% CI 1.06, 1.09) per 10 µg m^−3^ increase in PM_2.5_, and 1.04 (95% CI 1.03, 1.06) per 10 µg m^−3^ increase in PM_10_. This study also indicated that associations with PM_2.5_ remained relevant below the current WHO standards of 10 µg PM_2.5_ m^−3^.

#### Health impacts due to components of particulate matter

Studies are only beginning to link health effects to specific chemicals identified in aerosols. Current guidelines and standards for PM are based on the mass of PM_2.5_, without consideration of chemical composition of the particles. Although it stands to reason that the chemical composition would be an important determinant of health impacts, relatively few studies have examined this specific issue. In the context of the Montreal Protocol, UV-dependent secondary PM (sulfate, nitrate, and secondary organics) is chemically distinct from primary, UV-independent, PM. Thus, the relative health impacts of secondary *vs*. primary PM are central to this assessment.

Several groups have examined exposure to elemental constituents of aerosols, e.g., Cu, Fe, K, Ni, S, Si, V and Zn in PM_2.5_ [92, 93, 97]. While most show increases in HRs with increasing atomic abundances, they cannot identify the contribution of secondary particulates, such as secondary organics, sulfates, and nitrates, that depend on UV radiation.

The composition of PM across the United States was modeled recently by Pye et al. [46], using the Community Multiscale Air Quality (CMAQ) model with improved representation of aerosol composition, and was analyzed for associations with mortality data (for 2016) for cardiovascular and respiratory disease. The median county-level cardiovascular and respiratory disease age-adjusted death rate was 320 per 100,000 population across 2708 counties, while the average concentration of PM_2.5_ was 6.5 μg m^−3^, with organic aerosols (OA) being the most abundant component at 2.9 μg m^−3^. They estimated that, across the United States, for every 1 μg m^−3^ increase in PM_2.5_, 'there is an increase of 1.4 (95% CI 0.5–2.3) cardiovascular and respiratory deaths per 100,000 people. The sensitivity appears much greater for OA, with increases of 1 μg m^−3^ leading to an increase of 8.1 (95% CI 5.4–11) cardiovascular and respiratory deaths per 100,000 people. Subdivision of OA into primary and secondary types showed greater sensitivity for the latter, especially for PM formed by the OH-initiated oxidation of natural VOCs, such as isoprene and terpenes, commonly emitted by vegetation. The importance of secondary organic PM is consistent with a likely role of ROS in tissue damage (Sect. 2.3).

In conclusion, early indications are that secondary aerosols, including SOA, may be particularly damaging. This is of direct relevance to the Montreal Protocol, since secondary aerosols are generated by UV-driven photochemistry. In many locations (e.g., the contiguous United States, see Fig. 4), secondary aerosols may be the largest and the most detrimental fraction of PM_2.5_.

#### Interactions of air pollution and temperature on health

Episodes of air pollution frequently occur in combination with extremes in temperature with synergistic effects on health depending on the pollutant(s) involved, the degree and direction of temperature change, and the characteristics of the geographic area and the populations affected. Reviews and meta-analyses of the adverse health effects from extremes of temperature (both highs and lows) have proliferated in recent years indicating just how much research in this area is being done due to concerns about climate change [98–106].

A study from nine European cities by Analitis et al. [107] reported that the daily number of deaths increases by 2.20% (95% CI 1.28–3.13) on days with high O_3_ per 1 °C increase in temperature. The interaction of temperature with PM_10_ was significant for cardiovascular causes of death for all ages (2.24% on days with low PM_10_ (95% CI 1.01–3.47), while it was 2.63% (95% CI 1.57–3.71) on days with high PM_10_.

In a recent meta-analysis, Areal et al. [108] showed that effects of air pollutants were modified by high temperatures, leading to higher mortality from respiratory diseases and an increase in hospital admissions. The effect of PM_10_ during higher temperatures increased the risk of mortality by 2.1%, and for hospital admissions the effects increased by 11%. The effects of ground-level O_3_ during high temperatures were similar [108].

#### Health impacts of air pollution in vulnerable populations

Air pollution affects people from the beginning to the end of life, causing a wide range of acute and chronic diseases. Sensitive populations include, among others, children, the elderly, and people with existing chronic diseases. Accordingly, people with cardiovascular diseases are more likely to suffer a heart attack, stroke, or death when exposed to air pollution [109].

Ambient air pollution not only contributes to adverse health outcomes in individuals after birth, but it may also have immediate adverse impacts on reproductive processes. Animal and epidemiological evidence demonstrates that air pollution may influence fertility. A large Danish study investigated 10,183 participants between 2007 and 2018 [110] who were trying to conceive. The study showed that higher concentrations of PM_10_ and PM_2.5_ were associated with small reductions in fecundability, for example, the reductions in fecundability ratios from a one interquartile range (IQR) increase in PM_2.5_ (IQR = 3.2 µg m^−3^) and PM_10_ (IQR = 5.3 µg m^−3^) during each menstrual cycle were 0.93 (95% CI 0.87–0.99) and 0.91 (95% CI 0.84–0.99).

In another study on exposure to air pollution and the risk of pre-term birth, the authors investigated 2.7 million births across the state of California from 2011 to 2017 [111]. This study found an increased risk of pre-term birth with higher concentrations of PM_2.5_ [adjusted relative risks (aRR) (per interquartile increase)] = 1.04, (95% CI 1.04–1.05) and particulate matter from diesel exhaust, aRR = 1.02 (95% CI 1.01–1.03). Similar results were observed in another study from California, where the authors investigated 196,970 singleton pregnancies between 2007 and 2015. These authors found that, during cold seasons, increased exposure to PM_2.5_ during the three days prior to the premature birth was associated with 5–6% increased odds of very-early pre-term birth (OR_lag3_ 1.06, 95% CI 1.02–1.11). These studies confirm results from human and other animal studies that air pollutants can enter a pregnant female’s circulatory system and exert many deleterious health effects in multiple body organs including the placenta and the developing fetus [111].

In the umbrella review discussed above, Markozannes et al. [79] also found strong associations for a number of pregnancy/birth related outcomes. These included:

1. a 10 µg m^−3^ increase in PM_2.5_ for various durations of exposure was associated with an increased risk of having an infant born small for gestational age, (a) long-term exposure entire pregnancy OR: 1.151, 95% CI 1.104–1.200; (b) long-term exposure first trimester: OR: 1.074, 95% CI 1.046–1.103; (c) long-term exposure last trimester: OR: 1.062, 95% CI 1.042–1.083;
2. a 13 µg m^−3^ increase in SO_2_, (24 h average) was associated with an increased risk of low birthweight OR: 1.035, 95% CI 1.031–1.049, as was
3. a 10 µg m^−3^ increase in PM_10_ (long-term exposure; mean difference 7.42 g, 95% CI 8.10–6.75;
4. a 10-µg m^−3^ increase in PM_2.5_ for the third trimester was associated with an increased risk for hypertension during pregnancy OR: 2.177 95% 1.710–2.773.

There is also growing evidence that exposure to air pollutants maybe detrimental to the central nervous system and contribute to deficits in cognitive development, neurodegenerative diseases and dementia [112, 113]. A recent review [113] found that, despite a substantial increase in publications, there is only suggestive evidence that air pollution may influence late-life cognitive health as there is still substantial heterogeneity of findings across the studies. The strongest effect found was with respect to PM_2.5_ and cognitive decline. The review included two different outcomes, namely, incidence of dementia and abnormal neuroimaging. Since then, a large Canadian study investigated the effect of exposure to air pollution and incidence of dementia in ~ 2.1 million individuals [114]. The study identified 257,816 incident cases of dementia and found a positive association between an interquartile range (IQR) increase in PM_2.5_ of 4.8 µg m^−3^ and incidence of dementia, with a hazard ratio (HR) of 1.04 (95% CI 1.03–1.05) and an IQR increase of 26.7 μg m^−3^ in NO_2_ HR = 1.10 (95% CI 1.08–1.12) over a 5-year period, respectively. A similar large study using data from Medicare from the United States examined ~ 2.0 million incidences of dementia cases [115]. Per IQR increase in the 5-year average PM_2.5_ (3.2 µg m^−3^) and NO_2_ (22 µg m^−3^), they found an association with the development of dementia HR = 1.060 (95% CI 1.054–1.066) and with exposure to NO_2_ HR = 1.019 (95% CI 1.012–1.026), respectively. The authors also observed significant associations between exposure to PM_2.5_ and the development of Alzheimer’s disease HR = 1.078 (95% CI 1.070–1.086) and NO_2_ exposure HR = 1.031 (95% CI 1.023–1.039). The results of these new studies lend support to the theory that there is an association between air pollution and dementia and Alzheimer’s disease.

### Effects of tropospheric ozone and particulates on plants 

Photochemical air pollution can damage plants, with potentially adverse effects on agriculture and other natural resources. Ground-level ozone is a particular concern, since numerous studies have demonstrated significant damage [78, 116]. Other air pollutants co-produced with O_3_, e.g., peroxyacetyl nitrate (CH_3_C(O)O_2_NO_2_) are also phytotoxic, although their specific effects are difficult to separate from those of O_3_ [117]. The understanding of mechanisms and mitigation of these effects has improved, and some effects of particulates on plants are assessed below.

#### Effects of tropospheric ozone on health and yields of plants 

In the previous Quadrennial Assessment [6], we evaluated the adverse effects of O_3_ on crop and other plants. We noted that tropospheric O_3_ could contribute to significant losses in quality and yield of crops, e.g., 10–36% for wheat and 7–24% for rice. The adverse effects of O_3_ on plants continue to be documented in the literature. A metanalysis of 48 studies on the exposure of soybeans to tropospheric O_3_ conducted between 1980 and 2019 showed increases in degradation of chlorophyll and foliar injury. Leaf-area was reduced by 21%, biomass of leaves by 14%, shoots by 23%, and roots by 17% [118]. Chronic exposure to O_3_ of about 150 μg m^−3^ caused a decrease in yield of seed by 28%. In a study in Argentina [119], exposures of soybeans (a sensitive crop) to O_3_ at a concentration of 274 μg m^−3^ for 7 days resulted in a reduction in below-ground biomass of 25%, a 30% reduction of nodule biomass, and a 21% reduction of biological nitrogen fixation. Effects were more severe in tests with soils of low fertility where production of seed and seed protein was reduced by 10% and 12%, respectively. These effects in soybean exposed to O_3_ at 160 μg m^−3^ for 7 days were linked to decreases in metabolism of carbon and capacity for detoxification in the roots of soybean [120]. A study on the historical losses to air pollutants in maize and soybean grown in the United States showed that improvements in the control of O_3_, SO_2_, PM, and NO_2_ have improved yields by an average of 20% [121]. Of these pollutants, PM and NO_2_ appeared to cause more damage than O_3_ and SO_2_. Overall, the improvement in yields was equivalent to *ca* US$ 5 billion.

Observations between 2015 and 2018 in the province of Henan in China [122] showed that annual losses in yield of wheat exposed to O_3_ at concentrations above 80 μg m^−3^ were 12.8, 8.9, 10.8, and 14.1%. These were equivalent to annual losses of US$ 2.14, 1.32, 1.68, and 2.16 billion, respectively. A model was developed to extrapolate these losses to other crops in China [123]. Based on a 4-year average of tropospheric concentration of O_3_, estimated losses in wheat were 50 million tons per year, mostly in winter wheat (48 million tons); 21 million tons in rice; 18 million tons in maize and 1.6 million tons in soybeans [123]. A separate modeling study estimated that current concentrations of O_3_ reduced yield by 6.9% for rice and 10.4% for wheat [124]. Clearly, tropospheric O_3_ has significant adverse effects on food security in some countries and this might be exacerbated in the event of super-recovery of stratospheric ozone.

A modeling study on the effects of measured concentrations of O_3_ on grapes in the Demarcated Region of Douro in Portugal indicated that, in 2 years of high levels of O_3_, productivity of grapes was reduced by 27% and sugar content by 32% [125]. Similar effects were echoed in other grape-growing regions across the globe [126].

Crops are not the only class of plants to suffer reductions in yields from exposure to air pollutants. Forests are important sources of wood and fiber and can be affected by tropospheric air pollutants such as O_3_. In an analysis of the impacts of O_3_ on production of forests in Italy, Sacchelli et al. [127] calculated that the average cost of potential O_3_ damage to forests in Italy in 2005 ranged from 31.6 to 57.1 million € (i.e., 10–17 € ha^−1^ year^−1^). This damage resulted in a 1.1% reduction in the profitable forest areas. Estimated decreases in the annual national production of firewood, timber for poles, roundwood and wood for pulp and paper were 7.5, 7.4, 5.0, and 4.8%, respectively. A study on the effects of O_3_ on trees in Mediterranean forests in Istria and Dalmatia showed that current levels cause inhibition of growth for two species of oak (*Quercus pubescens* and *Q. ilex*) as well as pine (*Pinus nigra*) [128]. A climatological modeling study in European forests has shown that climate change has lengthened the growing season by *ca* 7 days decade^−1^ [129]. Because of this, the total phytotoxic dose of O_3_ taken up by trees over the season has increased and outweighs the benefits of a decrease in concentration of tropospheric O_3_ (1.6%) that resulted from measures to control pollution between 2000 and 2014.

Because of their sensitivity, the potential effects of O_3_ in the environment have been more extensively studied in plants than in animals. However, a recent study has focused on the effects of O_3_ in amphibians [130]. The authors exposed tadpoles of the midwife toad (*Alytes obstetricans*) to air-borne O_3_ at concentration up to 180–220 µg m^−3^ for 8 h per day from an early stage of development (limbs not yet formed) to metamorphosis. This is equivalent to the maximum concentrations observed in the Sierra de Guadarrama Mountains over a period of 10 years. The measured responses were successful development and infection of the developing tadpoles with the aquatic fungus *Batrachochytrium dendrobatid*is (*Bd*), which causes the disease known as chytridiomycosis. Airborne concentrations of O_3_ were measured in the exposure chambers but not in the water containing the tadpoles, so that actual dose could not be calculated. Results suggested that, at the greatest air-borne exposure, development of the tadpole was delayed and that susceptibility to *Bd* was increased. This study is preliminary and further work is needed to elucidate potential effects.

In summary, future changes in UV-B radiation will influence ground-level O_3_ and other pollutants. The recovery of stratospheric O_3_ to 1980 levels is expected to contribute 1–2 µg m^−3^ to ground-level O_3_ outside major urban areas [14, 15, 36], but super-recovery under some future climate scenarios could lead to larger increases (Sect. 2.2, and Box 1). This could be offset by further reductions in NO and VOC emissions, so the actual O_3_ concentrations will depend on local and regional air quality control measures, as well as the impacts of climate change and the Montreal Protocol on stratospheric O_3_. These impacts could affect both food security and forests.

#### Toxicological mechanisms

Effects of ozone on plants are mediated by the formation of free radicals in the tissues of the plants. Ozone enters the leaf of the plant through the stomata and forms ROS, which include O_2_^•−^, H_2_O_2_, OH, ^1^O_2_, as well as reactive carbonyl species such as malondialdehyde and methylglyoxal [131]. These reaction products damage components of the cells but also stimulate signaling systems, such as the release of isoprene [132] to activate defense mechanisms. These defenses include physical actions, such as closure of the stomata, biochemical responses such as the release of superoxide dismutase, catalase, and peroxidases to destroy the ROS, and release of chemical buffers, such as ascorbic acid, glutathione, phenolic chemicals, flavonoids, proline [133], and other amino acids, carotenoids, tocopherols, polyamines, and sugars [131].

**Fig. 5:**
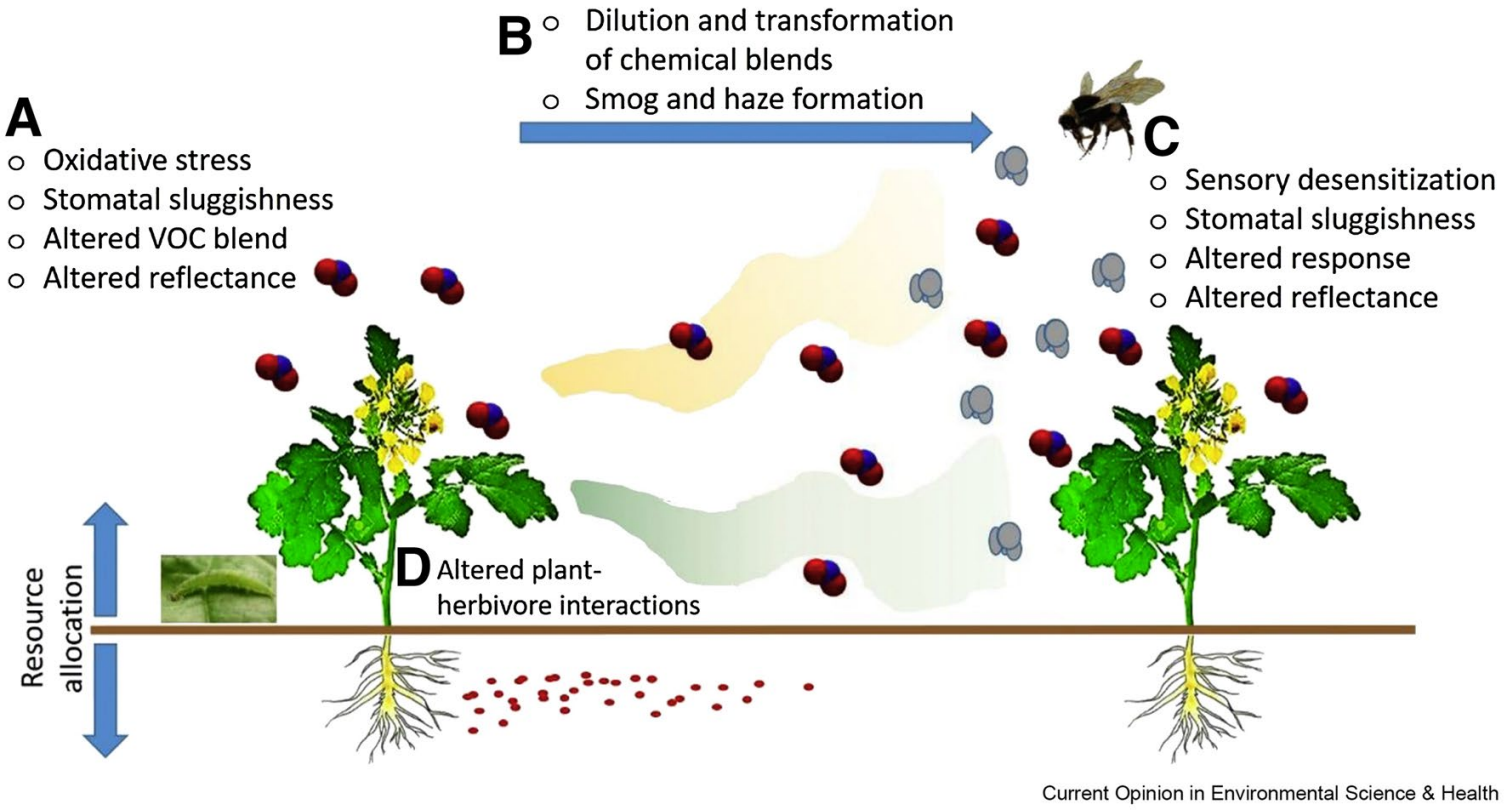
Sites at which air pollution can affect interactions mediated by olfactory or visual cues between plants and their associated community. **A** Effects of pollutants on signal-emitting organisms. **B** The degradation of VOCs by air pollutants and formation of reaction products and secondary organic aerosol (SOA). **C** Effects on the signal receiving organisms, e.g., pollinating insects. In addition, exposure to air pollution can influence the interactions between herbivores and plants. **D** From [134], reproduced with permission

Some adverse effects of tropospheric air pollutants on plants are indirect. For example, air pollutants can affect visual and chemical signals that mediate interaction between plants, and organisms that depend on plants or that are needed for the sustainability of plant communities [134] (see Fig. 5). For example, tropospheric O_3_ can destroy or change biogenic volatile chemicals and, thus, interfere with attraction of pollinators or pests to plants [135]. O_3_ could also interfere with sensory organs and the ability of pollinators to sense sources of nectar or the ability of biological-control organisms to sense their target hosts [134]. Also, physiological responses to damage from O_3_ might alter the ratios of pigments in plants, the phenology (seasonal development) of flowers and whole plants [136], or the time of flowering, thus affecting host recognition and pollinators. Pollutants may also affect reproduction in plants by directly damaging air-borne pollen through stimulating repair mechanisms and redirecting resources to cell repair rather than reproduction [137, 138]. In addition, the allergenicity of pollen can be enhanced with implications for human health [137].

#### Effects of particulates on plants

The effects of PM on plants were previously assessed to be few and minor [5, 6], but new studies suggest they may be important particularly in polluted areas. In a study of water-extractable chemicals collected from PM_2.5_ samplers on roadsides in Hungary, 13 common roadside plants were tested for sensitivity using the OECD-227 guideline test [139]. Endpoints measured in the study were shoot weight, shoot height, visible symptoms of damage on the plants, growth rate, photosynthetic pigments, and activity of peroxidase enzyme. The authors concluded that particulate pollution derived from traffic added substantial additional stress to communities of plants found on roadsides. The study was conducted during mid-winter and near sources, implying that most of the PM was primary rather than secondary, and hence insensitive to any changes in UV radiation. It is unclear if similar plant damage would have been caused by UV-dependent secondary aerosols.

In another study, the combined effects of ambient atmospheric O_3_ and particulate matter on wheat were assessed [140]. The cumulative concentration of ambient O_3_ above the threshold of 80 μg m^−3^ h^−1^ during the 4-month study was 453 μg m^−3^ h^−1^. Concentrations of ambient PM_2.5_ and PM_10_ ranged between 45–412 μg m^−3^ and 103–580 μg m^−3^, respectively. Controls were cleaned of particulates and were protected from O_3_ by treatment with ethylene diurea, a mitigator of ozone-stress. Economic yield was reduced 34% in wheat exposed to O_3_ and PM, 44% in wheat exposed to PM only and 52% in plants exposed to O_3_ alone. Similar observations were reported in a modeling analysis of the effects of O_3_ and PM on the yields of wheat and rice in China [124]. Based on current levels of O_3_ and aerosols, their results indicated that anthropogenic aerosols reduced yield of rice and wheat by 4.6 and 4.7%, respectively. The authors suggested that this was because of the effect of dimming of photosynthetically active radiation by aerosols but that there were some benefits from cooling and nutrients provided via the aerosols. The losses due to both O_3_ and aerosols were estimated to be 11.3 for rice and 14.6% for wheat. The relative contributions of primary and secondary PM were not reported, so that the sensitivity to changes in UV radiation remains unclear.

Overall, these results indicate that aerosols and tropospheric O_3_ alone, or in combination, have adverse effects on plants and yields of crops. The loss from O_3_ is greater than that from aerosols but they do act additively. With few studies on this interaction, the potential for additive and/or synergistic effects of O_3_ and particulates, the significance to crop plants and human activities is uncertain. However, it is likely that these effects will be localized and could be mitigated by increased controls of tropospheric air pollutants.

### Self-cleaning capacity of the atmosphere

The hydroxyl radical (OH) is the major oxidant in the troposphere and its concentration largely determines the lifetime of many tropospheric pollutants. It is produced via UV-B photolysis of O_3_ (see Fig. 1). Hence, increases in the tropospheric concentration of OH are, in part, a consequence of increasing emissions of ODSs. The tropospheric concentration of OH is a balance between OH production and consumption, where both rates are also affected by climate change.

**Fig. 6:**
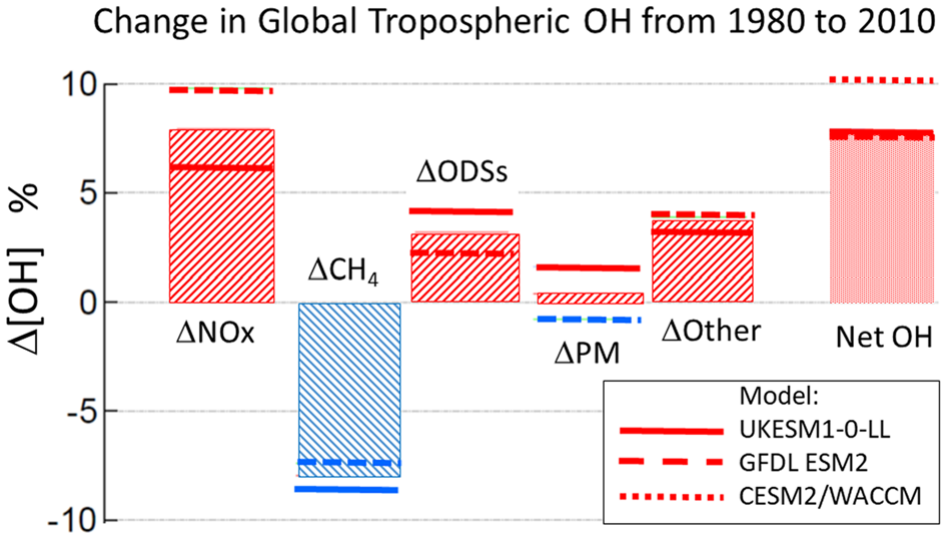
Relative change in global concentrations of tropospheric OH from 1980 to 2020, estimated with three different Earth System Models (ESMs). The net change in OH (rightmost column) has contributions from increased emissions of nitrogen oxides and other precursors of tropospheric ozone (ΔNOx); increased emissions of methane (ΔCH_4_); accumulation of ozone-depleting substances now regulated under the Montreal Protocol (ΔODSs); emissions of particulate matter and its precursors (ΔPM); and other undifferentiated changes attributed to underlying climate change (e.g., water vapor), as well as interactions among these separate factors (ΔOther) (modified from [141])

Global mean concentrations of tropospheric OH have been calculated to have changed little from 1850 to around 1980 [141, 142]. However, in the period 1980–2010 the modeled global tropospheric concentration of OH has increased, mainly because of increasing concentrations of precursors of tropospheric O_3_, and UV radiation [141]. According to the combined output of three computer models (Fig. 6), there was a net increase in OH of about 8% (mean value of the models). The main precursor of tropospheric O_3_ is NO_x_, the tropospheric concentration of which has increased over 1980–2010 (Fig. 6). Global emissions of NO_x_ peaked around 2012, followed by reductions [143]. In addition to NO_x_, also ODSs and factors underlying climate change such as rising water vapor have contributed to the net increase in modeled OH from 1980 to 2010. Increasing atmospheric CH_4_ was the main factor counteracting the trend of rising OH (by about − 8%, see Fig. 6) in this period.

Studies that infer concentrations of OH by the rate of removal of chemicals from the atmosphere have generally indicated a decreasing trend in OH after 2000 [144]. However, the interannual variability in OH from these studies was large, i.e., the difference between modeled and measured OH trends were not statistically significant [141]. For such studies, methyl chloroform (CH_3_CCl_3_) is often used [145] to infer concentrations of OH. The drawback of this method is that the emissions (almost entirely anthropogenic) of CH_3_CCl_3_ have declined substantially in the last 30 years [145], thus affecting the accuracy and precision of derived amounts of global OH.

Future trends in tropospheric concentrations of OH not only depend on solar UV-B radiation and on the concentration of precursors of OH but also on OH sinks, particularly CO and CH_4_. As discussed above, the increasing global concentration of CH_4_ was the main factor counteracting positive modeled OH trends in the period 1980–2010 (Fig. 6). Total global emissions of CH_4_ are currently ~ 525 Tg year^−1^ [146]. If emissions of CH_4_ from anthropogenic and natural sources continue to rise as they have since 2007, this could decrease global mean OH by up to 10% by 2050 [147], increasing the atmospheric lifetime and concentrations of CH_4_ in a positive feedback. Also, emissions of CO, the major sink of OH, may increase as a result of more frequent and longer lasting wildfires related to climate change. A change in the average concentration of OH in the troposphere would have large impacts on the cleaning capacity of the troposphere.

**Fig. 7:**
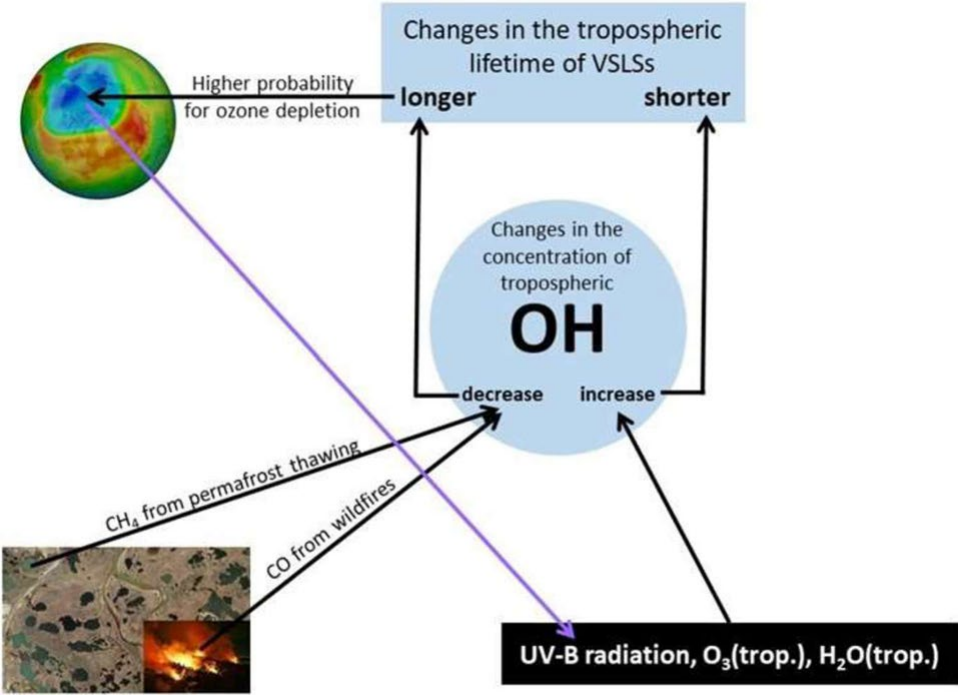
Interacting effects of UV-B radiation and climate change on tropospheric concentrations of OH and on the lifetime of very-short-lived substances (VSLSs). Effects of climate change include more frequent wildfires and thawing of permafrost soils with the formation of thermokarst lakes, which are important sources of CO and CH_4_, respectively. Increased emissions of CO and CH_4_ tend to decrease the tropospheric OH concentration, which in turn results in longer lifetime of VSLSs and, thus, a higher probability of stratospheric ozone depletion

Finally, we note the importance of reactions between tropospheric OH and gases that affect stratospheric ozone (Fig. 7). These include anthropogenic halogenated organics (the HCFCs and HFCs, specifically selected for their reactivity with OH so that they are removed in the troposphere), as well as gases such as CH_4_ and VSLSs. Hydroxyl radicals control the tropospheric lifetimes of these gases, and hence their ability to reach the stratosphere. VSLSs are important pollutants since they can reach the lower stratosphere, despite their tropospheric lifetime of less than 6 months, and contribute to depletion of stratospheric O_3_ [148, 149]. These chemicals are not controlled by the Montreal Protocol and include chlorinated, brominated, and iodinated VSLSs (Cl-VSLSs, Br-VSLSs, and I-VSLSs, respectively). Cl-VSLSs are mostly of anthropogenic origin, while I-VSLSs and Br-VSLSs, particularly bromoform (CHBr_3_) and dibromomethane (CH_2_Br_2_) are mainly produced in biotic processes and are affected by climate change, including increased coastal runoff and thawing of permafrost [148]. The contribution of BrVSLSs to the total stratospheric bromine loading was estimated to be ≈ 25% (in 2016) [150]. The mixing ratio of Br-VSLS at the tropopause has been measured to increase with latitude in the Northern Hemisphere, particularly during polar winter [149] when photochemically driven losses are smallest. This results (via troposphere-to-stratosphere transport) in higher concentrations of Br-VLSLs in the extratropical lower stratosphere, as compared to those in the tropical lower stratosphere [149].

The major sink of halogenated VSLSs is reaction with OH. Hence, the tropospheric lifetime of VSLSs mainly depends on the tropospheric concentration of OH. For example, Rex et al. [151] found a lifetime of dibromomethane (CH_2_Br_2_) as long as 188 days inside an OH minimum zone over the West Pacific, while outside the OH minimum zone, the lifetime of CH_2_Br_2_ was 55 days. In addition to CHBr_3_ and CH_2_Br_2_, methyl bromide (CH_3_Br) is an ODS. Due to the Montreal Protocol and its Amendments, atmospheric mole fractions of CH_3_Br have declined considerably and, at present, emissions of CH_3_Br primarily stem from natural sources [152], with some anthropogenic sources related to commercial quarantine and pre-shipment applications. The production of CH_3_Br in seawater is a biological process mediated by phytoplankton such as diatoms [153]. The interannual variability of atmospheric CH_3_Br concentrations cannot be solely explained by changes in the biological production of CH_3_Br due to changes in sea-surface temperatures (SSTs) and stratification [152]. Also, sinks of CH_3_Br have to be considered, where the major atmospheric sink of CH_3_Br is reaction with OH. Nicewonger et al. [152] found a strong correlation between the interannual variability of CH_3_Br and the Oceanic Niño Index (ONI) from 1995 to 2020. About 36% of the variability in global atmospheric CH_3_Br was explained by the variability in El Niño Southern Ocean (ENSO) during this period, with increases in CH_3_Br during El Niño and decreases during La Niña [152]. One reason for increases in atmospheric CH_3_Br concentrations during El Niño years (positive ONI) could be a global reduction in OH during El Niño years. Based on modeling studies for the period 1980 to 2010, Zhao et al. [142] found decreases in global concentrations of OH during El Niño years that were mainly driven by an elevated loss of OH via reaction with CO from enhanced burning of biomass (Fig. 7). The longer the tropospheric lifetime of halogenated VSLSs, the higher is the probability that they reach the stratosphere and contribute to depletion of stratospheric O_3_ with impacts on ground-level UV-B radiation. Since UV-B radiation, together with tropospheric O_3_ and water vapor enhance the formation of OH, increased levels of UV-B radiation could counterbalance decreasing concentrations of OH due to wildfires and thawing of permafrost soils (Fig. 7).

### Changes in atmospheric circulation and transport of pollutants

#### Ozone from the stratosphere

Ozone as an air quality issue has normally been considered as a local or regional issue. However, O_3_ from the stratosphere is also transported to the troposphere where it contributes an important but variable fraction of O_3_ at ground level and represents a baseline upon which locally or regionally generated O_3_ is added. This is known as stratospheric–tropospheric exchange (STE). The magnitude of the contribution of stratospheric O_3_ to tropospheric O_3_ is difficult to quantify but important. A comparison of measurements and 3 different models estimated the influence of stratospheric O_3_ on tropospheric O_3_, highlighting the challenges of obtaining consistent results [154]. The study estimated the fraction of O_3_ near the Earth’s surface that can be attributed to O_3_ transported down from the stratosphere. This was found to vary between 10% year-round in the tropics increasing to greater than 50% at mid to high latitudes in winter.

**Fig. 8:**
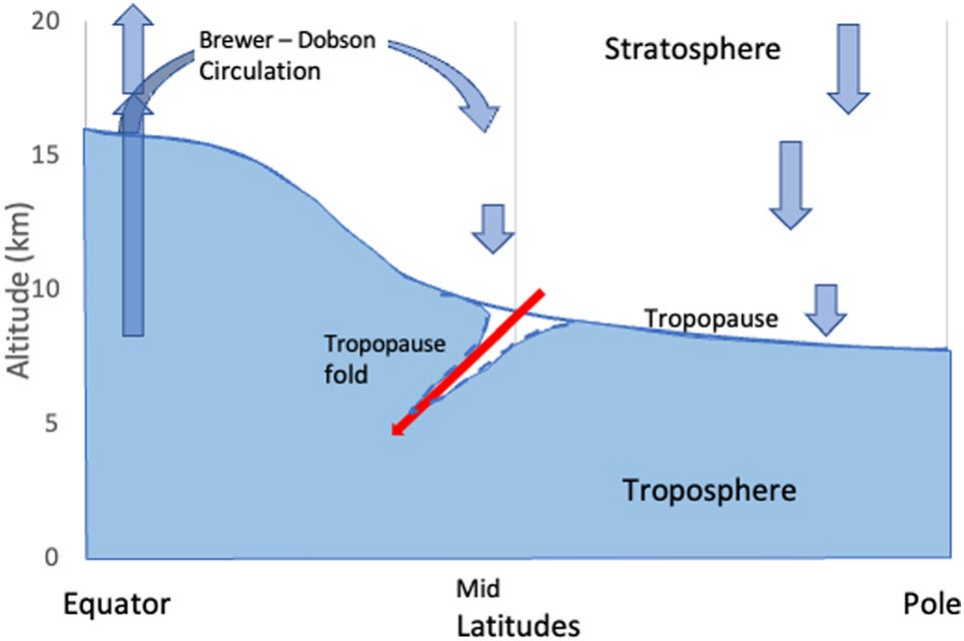
Schematic of the circulation of air into the stratosphere and its return. The general circulation is slow relative to movements in the troposphere. Tropopause folds (shown with a dotted blue line) occur sporadically and inject stratospheric air into the troposphere

The amount of O_3_ transported from the stratosphere to the earth’s surface is likely to change due to human activities. Stratospheric O_3_ is transported to the troposphere as part of the Brewer-Dobson circulation, which describes the turnover of stratospheric air (Fig. 8). Part of this is the transport of tropospheric air into the stratosphere in the tropics, and the return of (O_3_-rich) air in the troposphere of the subtropical regions. Recovery of stratospheric O_3_ because of decreasing release of ODSs would be expected to increase the amount of O_3_ transported into the troposphere. However, the impact also depends on the strength of the meteorology driving STE, which is sensitive to changes in climate.

Estimates of STE are poorly characterized by observations and atmospheric models. An assessment of amounts of O_3_ in the troposphere shows that model estimates of STE were around 1000 Tg year^−1^ O_3_ for results reported in 1995 but by 2015 models provided estimates approaching 400 Tg year^−1^, with a multi-model estimate of 535 ± 160 Tg year^−1^ for the year 2000 [155]. The IPCC AR6 assessment reports a value of 628 ± 800 Tg year^−1^ for 2010, with the large uncertainty highlighting how poorly this value is known [11]. Other recent estimates include 347 ± 12 Tg year^−1^ (2007–2010) [156] and 400 ± 60 Tg year^−1^ (1990–2017) [157].

Typically, the magnitude of the STE is inferred as the difference between the calculated production and loss of O_3_ (termed the residual) rather than modeling STE transport itself [158]. These production and loss terms are an order of magnitude larger (around 5000 Tg year^−1^) than the estimated transport [e.g., 159], so that their difference is highly uncertain. A second confounding factor is that models have used different definitions of the upper boundary (tropopause) of the troposphere.

**Fig. 9:**
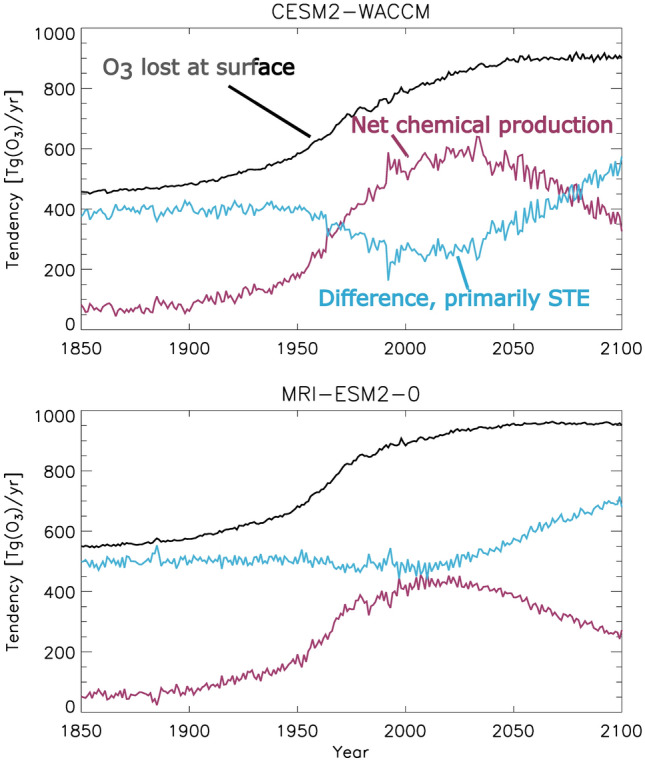
Drivers of tropospheric ozone concentration (redrawn with permission and assistance from Guang Zeng from Fig. 13, in [158]). The deposition amount is directly related to the concentration of O_3_ at the surface of the Earth. The two panels represent the output of different chemistry–climate models considered in the study. STE, stratospheric–tropospheric exchange

The modeling of the impact of STE on tropospheric O_3_ for the period 1850–2100 shows a significant decrease in O_3_ from the stratosphere by the year 2000 [158] (see Fig. 9). A modeling study focusing on the period 1980–2010 calculated a decrease in the transport of O_3_ from the stratosphere to the troposphere due to the impact of ODSs on stratospheric O_3_ [160]. The model estimated a 4% decrease (14 Tg O_3_) in global tropospheric O_3_ resulting from ODS up until 1994. Another study using measurements of N_2_O to constrain the atmospheric modeling estimated an average decrease in STE due to the Antarctic ozone hole (1990–2017) of 30 Tg year^−1^ with a range of 5–55 Tg year^−1^, depending on year [157].

In contrast, the results of a modeling and observational study of the regional changes in O_3_ concentration in the troposphere for the period 1980–1990 through to 2000–2010 [154] suggest an increase in the concentration of O_3_ at the Earth’s surface, but the other studies (noted above) found that stratospheric O_3_ led to a small increase over this period in the Northern Hemisphere and no significant change in the Southern Hemisphere. Clearly more work is needed in this area.

For the period from 2000 to 2100, substantial increases in the amount of O_3_ transported from the stratosphere to the troposphere are predicted [158]. Estimates using the output from seven atmospheric models and focusing primarily on RCP6.0, [161] suggest a 10–16% increase in the amount of O_3_ in the troposphere from STE in the twenty-first century. When assessing the relative importance of changes in GHGs *vs* ODSs in driving the changes in STE, they did not obtain a consistent picture from the models, although it appears that the two factors are of similar magnitude [161]. However, there is insufficient agreement between models to quantify trends. The net change in the concentration of O_3_ in the troposphere by 2100 is very dependent on the magnitude of anthropogenic emissions. The decrease in net chemical production (red curve, Fig. 9) is driven by the predicted controls on the emission of air pollutants. Calculations using the RCP8.5 scenario showed a marked increase in the concentration of O_3_ in the troposphere, with a threefold larger amount of O_3_ transported from the stratosphere than the RCP6.0 scenario [161]. It is not possible to infer the magnitude of the changes in O_3_ at ground level from these models, as they report O_3_ concentrations averaged for the entire vertical extent of the troposphere, and the impact of stratospheric O_3_ is much larger in the upper troposphere than at the Earth’s surface.

Folds in the tropopause have a direct impact on air quality at ground level. These folds are not uniformly distributed longitudinally [162, 163] and are common over the Eastern Mediterranean, where they have been identified as a significant cause of elevated concentrations of O_3_ at ground level that are greater than the European Union air-quality standards [164]. The equivalent effect is also observed in the Southern Hemisphere over the Indian and Southern Oceans. A modeling study of tropopause folds for the period 1960–2100, using emissions as specified in RCP6.0 and including stratospheric O_3_ recovery, suggests that folds will increase during this period. Statistically significant changes in the number of tropopause folds of around 3% have been identified in regions that coincide with a calculated increase of 6 µg m^−3^ in O_3_ near the Earth’s surface [165].

Quantifying the transport of O_3_ from the stratosphere is therefore important to understanding tropospheric air quality but remains difficult. The challenge in measuring and modeling STE of O_3_ is partially due to the mechanism by which the downward transport occurs. Air rich in O_3_ is injected into the troposphere at the edges of the tropics via “folds” (Fig. 8), where thin layers of air from the stratosphere are surrounded (vertically) by air from the troposphere and vice versa. These layers then mix. Methods for identifying folds within model output are being improved [e.g., [165] and showing some promising consistency among different models [162].

The modeling of STE is also hampered by the relatively few measurements of the chemical composition and physical structure of the atmosphere in the upper troposphere and lower stratosphere. As a result, there is little information that can be used to constrain atmospheric models. Efforts are now underway to use measurements of the chemical composition of air on commercial aircraft to build up a robust climatology, which can help modeling [166]. Similarly, there are ongoing efforts to improve the use of measurements of O_3_ by satellites in atmospheric modeling [167], and potentially O_3_ sondes and in situ measurements. Using observations to constrain models introduces sensitivity to changes in quality and calibration of the input data and this requires careful assessment [167, 168] In future, these data should allow better quantification of the changes in tropospheric O_3_ that are caused by changes in stratospheric O_3_.

#### Effects of circulation changes on extreme weather events and air quality 

Air quality is also affected by extreme weather events, such as wildfires. Changes in weather patterns, including extreme weather events, are not only caused by climate change but also by polar stratospheric ozone depletion, which strengthens the stratospheric polar vortex. Changing weather patterns due to the Antarctic ozone hole have been observed in the Southern Hemisphere [23, 169, 170]. For example, anomalies in rainfall and droughts in the Southern Hemisphere are correlated with the duration of the Antarctic Ozone hole [171]. In addition to the strength of the stratospheric polar vortex, the El Niño Southern Oscillation and the Indian Ocean Dipole also affect weather conditions in Australia [172]. Hot and dry weather increases the risk of wildfires. The severe fire season in Australia 2019–2020 led to significant degradation of air quality within Australia and a smoke plume that was traced around the globe [173–175]. The likelihood of wildfires is increasing globally, a trend that is expected to continue [176]. However, the recovery of stratospheric O_3_ should decrease the stability of the Antarctic polar vortex, which should lead to wetter conditions in the Southern Hemisphere in the near future for this region.

Similarly to the effects of the atmospheric dynamics of the Antarctic ozone hole, Arctic stratospheric ozone depletion results in a shift of the Arctic Oscillation (AO) to more positive values (e.g., [177]) and a more zonal Northern Hemisphere jet stream. Consequences are colder than normal surface temperatures in southeastern Europe and southern Asia, but warmer than normal surface temperatures in Western Europe, Russia, and northern Asia [178]. For example, a likely consequence of the unprecedented Arctic stratospheric ozone depletion in spring 2020 was the heat wave in Siberia accompanied by wildfires in this region [179]. Whether such events will occur in the future depends on trends in the emissions of ODSs and GHGs, since GHGs affect the Arctic stratosphere via radiative cooling [180]. Hence, the frequency of extreme weather events such as droughts and therefore wildfires in both hemispheres is influenced by direct effects of climate change and by changes in atmospheric circulation and in polar stratospheric O_3_. Wildfires decrease tropospheric air quality with the emission of PM, CO, and other tropospheric pollutants, which impact human health.

### Conclusions

Changes in stratospheric O_3_ concentrations, and thus in ground-level UV-B radiation, affect tropospheric air quality. Poor air quality remains a major health problem globally, despite progress in reducing emissions of primary air pollutants. Much of the impact of air pollutants is due to chemicals produced by UV-B-initiated photochemistry, including O_3_ and PM, i.e., secondary inorganic and organic aerosols. PM and tropospheric O_3_ pose a significant health risk. Overall, recovery of stratospheric O_3_, and hence lower intensity of ground-level UV-B radiation, is expected to slightly improve air quality in cities in mid-latitudes but slightly worsen air quality in rural areas. For PM, the impacts of changes in UV-B radiation on the amount and chemical composition of PM are still poorly understood.

Transport of O_3_ from the stratosphere into the troposphere adds to tropospheric O_3_ concentrations. This transport is expected to increase because of the recovery of stratospheric O_3_ and changes in global circulation driven by climate change. Given the current state of knowledge, estimating the magnitude of these changes remains a significant challenge.

UV-B radiation is also involved in the formation of OH, the major cleaning agent of the troposphere. Hence, UV-B radiation has some beneficial effects on tropospheric air quality. Reaction with OH drives the atmospheric removal of many problematic tropospheric gases including some pollutants and GHGs such as CH_4_, and VSLSs (noting also that GHGs and VSLSs affect stratospheric O_3_). Given current global CH_4_ emission of ~ 500 Tg year^−1^, a 1% decrease of the global OH concentration would result in an increase of ~ 1% in tropospheric CH_4_ concentrations, equivalent to a sustained increase in emissions of CH_4_ of ~ 5 Tg year^−1^.

The main sink of OH is reaction with CO. An important natural source of CO is wildfires, which have increased in frequency and intensity due to climate change. Hence, UV-B radiation and climate change affect concentrations of tropospheric OH with potential feedbacks on climate change and on stratospheric ozone.

The impact of poor air quality is not limited to human health; it affects plants and other organisms as well. This has had a substantial impact on food production and forests through exposure to ground-level O_3_. There is also evidence of reduced food production due to PM. The magnitude of these impacts will be altered by climate change and the future evolution of stratospheric O_3_.

## Trifluoroacetic acid in the global environment with relevance to the Montreal Protocol 

### Background

Trifluoroacetic acid (TFA) is the terminal breakdown product of many fluorinated chemicals, including those that fall under the purview of the Montreal Protocol and its Amendments. Its properties (discussed below) include very low reactivity, high stability, and recalcitrance to breakdown in the environment. This has raised concerns about the use of fluorinated substitutes for the ozone-depleting and the fluorinated greenhouse gases. The formation, fate, and potential effects of TFA has been the remit of the EEAP for the last two decades, and this overview is a continuation of this activity with a primary focus on new information since the last Quadrennial Assessment [6] to the Parties of the Montreal Protocol.

#### Classification of trifluoroacetic acid as a per- and poly-fluoroalkyl chemical

Trifluoroacetic acid CF_3_-COOH (CAS# 76-05-1) is a perfluorinated chemical, meaning that, aside from its functional group (-COOH), all hydrogen atoms in the molecule have been replaced with fluorine. The European Chemicals Agency has proposed that this chemical be included in a class, the per- and poly-fluoroalkyl substances (PFAS) [181]. Others have suggested that the definition of PFAS should exclude TFA and chemicals that degrade to just give TFA [182]. In 2022, there were 4730 chemicals in the PFAS class, which had been expanded to include all chemicals with at least one aliphatic –CF_2_– or –CF_3_ moiety. The PFAS class includes gases (such as those under the purview of the Montreal Protocol), low boiling point liquids, high boiling point liquids and lubricants, and solid polymers used in industry, medicine, and domestic equipment. As has been pointed out [183], a small number (about 256) of these PFAS are currently used commercially and “*grouping and categorizing PFAS using fundamental classification criteria based on composition and structure can be used to identify appropriate groups of PFAS substances for risk assessment*.” [183] More recently, a majority of a panel of experts agreed that “*all PFAS should not be grouped together, persistence alone is not sufficient for grouping PFAS for the purposes of assessing human health risk, and that the definition of appropriate subgroups can only be defined on a case-by-case manner*.” [184]. In addition, the majority opinion with respect to toxicology was that “*it is inappropriate to assume equal toxicity/potency across the diverse class of PFAS*” [184].

This same argument applies to the inclusion of TFA, with a two-carbon chain and a single CF_3_ group, into a class with longer-chain PFAS. These longer-chain PFAS have key chemical, physical, and biological properties that become quite different with increasing length of the carbon chain (Table 2 and Online Resource Table 1). For example (see Table 2), log K_OW_ (a measure of partitioning between lipids in organisms and water); Henry’s Law Constant (a measure of partitioning between water and air); *K*_OC_ (a measure of adsorption to soil and sediment); and the half-life in humans (related to chronic exposure and chronic toxicity) all vary with changes in the length of the carbon chain. These relationships are well recognized [185–189] as they are important drivers of adsorption, distribution, and excretion in animals, which are major determinants of adverse effects.

**Table 2:** Key physical, chemical, and biological properties of the linear perfluorinated carboxylic acids from 2 to 8 carbons

Property	Trifluoroacetic acid	Perfluoropropanonic acid	Perfluorobutanoic acid	Perfluoropentanoic acid	Perfluorohexanoic acid	Perfluoroheptanoic acid	Perfluorooctanoic acid
Abbreviation	TFA	PFPrA	PFBA	PFPeA	PFHxA	PFHpA	PFOA
CAS#	76-05-1	422-64-0	375-22-4	2706-90-3	307-24-4	375-85-9	335-67-1
Molecular formula	CF_3_COOH	CF_3_CF_2_COOH	CF_3_(CF_2_)_2-_COOH	CF_3_(CF_2_)_3_-COOH	CF_3_(CF_2_)_4_-COOH	CF_3_(CF_2_)_5_-COOH	CF_3_(CF_2_)_6_-COOH
Number of carbon atoms	2	3	4	5	6	7	8
Log K_OW_	0.5	1.5^a^	2.43^a^	3.262^a^	3.48	5.024 ^a^	5.905^a^
Henry’s Law Constant (atm m^−3^ mol^−1^)	1.11 × 10^–7^	4.43 × 10^–6 a^	0.0051 ^a^	0.029^a^	0.174^a^	1.521^a^	3.044^a^
K_oc_ (L kg^−1^)	0.17–20	12.7 ^a^	58 ^a^	270 ^a^	1247 ^a^	5761	30,440
NOEC most sensitive aquatic plant (ng L^−1^)	2.5 × 10^6b^	1.44 × 10^7c^	6.21 × 10^8c^	> 1.00 × 10^9c^	NA	> 1.02 × 10^9c^	5.80 × 10^3c^
NOEC most sensitive aquatic animal (ng L^−1^)	LC50 = 7 × 10^7d^	LC50 = 8.0 × 10^7d^	LC50 = 1.1 × 10^8 d^	LC50 = 1.3 × 10^8 d^	LC50 = 1.4 × 10^8 d^	LC50 > 1.02 × 10^6d^	LC50 = 1.5 × 10^8^
Half-life in humans ^f^	16 h	NA	72–81 h	NA	14–49 d^e^	1.2–1.5 year	2.1–10 year

Unless otherwise stated, references are from [190]

*NOEC* no observed effect concentration, *NA* not applicable

Other sources are: ^a^[191], ^b^[192], ^c^[193], ^d^[194], ^e^[187], ^f^[195]

There has been considerable discussion as to the inclusion of TFA in the class PFAS for regulatory purposes [182, 183, 196–199]. Regulatory agencies in North America acknowledge the physical, chemical, and biological properties of chemicals in the class of PFAS [189] and, in particular, the influence of chain length on these properties [188, 189, 200]. A sound assessment of the environmental impact of TFA needs to consider the relevant physical, chemical, and toxicological data and realistic environmental concentrations (see discussion in Sect. 3.2 to 3.6). We are of the opinion that the properties of TFA indicate that it should not be included in this class for the purposes of generic regulatory risk assessment.

The PFAS class also includes other perfluorinated chemicals that are of concern, e.g., perfluorooctanesulfonic acid, (PFOS). PFOS differs from TFA and its homologues because it is a sulfonic acid and is also more toxic than its alkanoic homolog. Therefore, as these chemicals do not fall under the purview of the Montreal Protocol, they have not been included in this discussion or in Table 2.

#### Properties of trifluoroacetic acid

The physical and chemical properties of TFA are well known [201] but key to assessing environmental risk is that it is a strong acid with a pKa of 0.3 and is completely miscible with water [190]. In the environment, it forms salts with alkali metals, which are also very soluble in water. These properties indicate that TFA and its salts will not bioaccumulate in organisms other than terrestrial plants and will not biomagnify in food chains. The carbon–fluorine bond is the strongest of all bonds with carbon and TFA and its salts are very recalcitrant in the environment. Studies on degradation by microbiota, including species and strains from contaminated areas, have not shown any evidence of TFA being susceptible to microbiological degradation [202, 203].

### Chemical pathways for degradation of precursors to trifluoroacetic acid

HCFCs and HFCs have found widespread use as replacements in applications that previously used CFCs. More recently, short-chain halogenated alkenes (hydrofluoroolefins, HFOs) are finding increasing use in several commercial applications. For example, *E*-CF_3_CH=CHF (HFO-1234ze(E)) and *E*-CF_3_-CH=CHCl (HCFO-1233zd(E)) are being used for foam blowing and in large chillers, whereas 2,3,3,3-tetrafluoropropene, CF_3_CF = CH_2_ (HFO-1234yf) is used as a replacement for 1,1,1,2-tetrafluoroethane, CF_3_CH_2_F (HFC-134a), in vehicle air conditioning units [204]. These chemicals are anthropogenic and there are no known natural sources of HCFCs, HFCs, and HFOs.

**Fig. 10:**
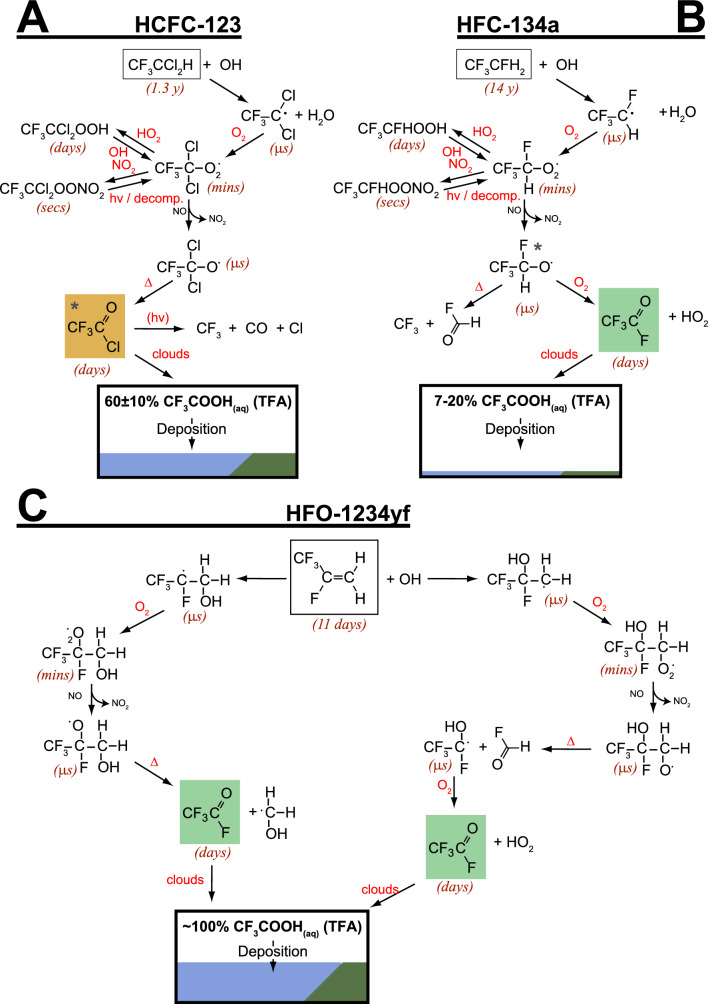
Atmospheric degradation pathways and corresponding yields of TFA for HFCF-123 (**A**), HFC-134a (**B**) and HFO-1234yf (**C**) representing three generations of important CFC replacements. Approximate atmospheric lifetimes for the chemical species involved are indicated in parenthesis. Species marked by an asterisk have significant competing fates in the atmosphere [205, 206]

Tropospheric degradation of HCFCs, HFCs and HFOs is initiated by reaction with OH radicals, leading to formation of small terminal degradation products including CO, CO_2_, and the halo-acids hydrogen fluoride (HF) and hydrogen chloride (HCl). Some of the degradation products are also atmospheric precursors of TFA through hydrolysis of acyl halides, e.g., CF_3_CFO, or via secondary photochemistry of trifluoroacetaldehyde (CF_3_CHO) [205, 206]. The chemistry by which the CFC replacements are converted into precursors of TFA has been extensively studied over the last few decades and recently summarized [207, 208]. Figure 10 illustrates how atmospheric degradation of different CFC replacements, belonging to three successive generations of CFC replacements, can lead to the formation of TFA in significantly different yields. For instance, the dominant atmospheric fate of CF_3_CClO and CF_3_CFO, generated in the atmospheric processing of HCFC-123 and HFC-134a, respectively, is uptake into cloud water, followed by effective hydrolysis to yield TFA, on a timescale of approximately 5–30 days [205]. However, due to competing fates of the intermediary alkoxy radicals (marked with asterisks in Fig. 10), the effective yields of TFA are significantly different (e.g., ~ 60% for HCFC-123 and 7–20% for HFC-134a). In the case of HFO-1234yf, no significant competition exists in the degradation pathway, and the expected yield of TFA is ~ 100% through the hydrolysis of CF_3_CFO.

Even if they are not producing acid-halides during their atmospheric degradation, some other CFC replacements can still form TFA in small yields through the formation of trifluoroaldehyde, CF_3_CHO. This aldehyde is the primary degradation product from several CFC replacements, including HCFCs, HFCs, HFOs and HCFOs, e.g., HCFC-234fb (CF_3_CH_2_CCl_2_F, *τ* = 45 years), HFC-143a (CF_3_CH_3,_ *τ* = 51 years) and HFO-1234ze(E) (*E*-CF_3_CH = CHF, *τ* = 19 days) and HCFO-1233zd(E) (CF_3_CH = CHCl, *τ* = 42 days) [209]. Here τ is the atmospheric lifetime defined as the reciprocal of the pseudo first-order rate constants for the removal of the chemical species, also sometimes referred to as the “*e*-folding lifetime”.

**Fig. 11:**
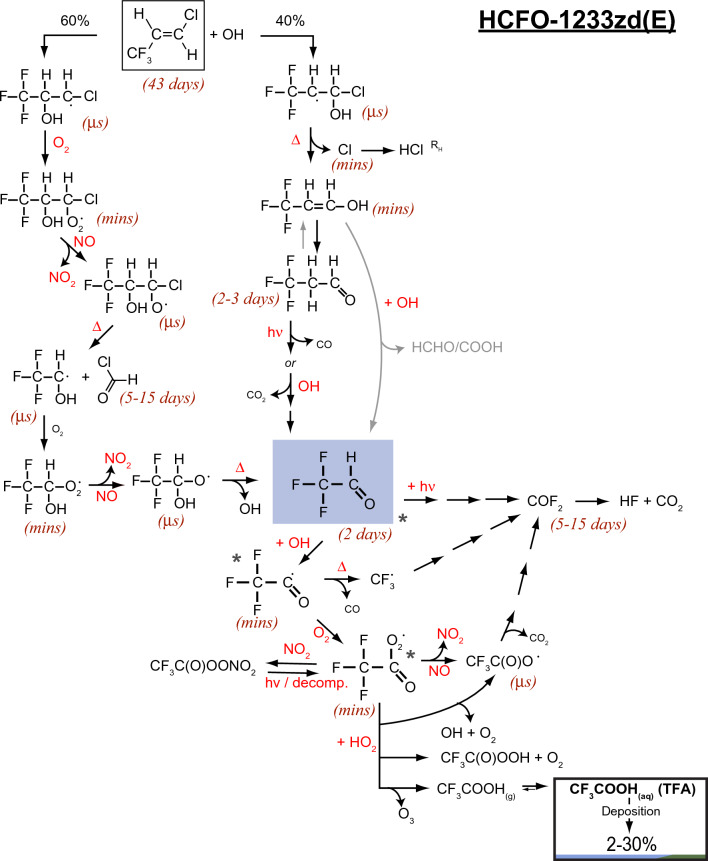
Atmospheric degradation of HCFO-1233zd. The OH-initiated oxidation of the product, CF_3_CHO, is a minor source of TFA. Based on [207] and [208]

Figure 11 illustrates the atmospheric degradation of HCFO-1233zd(E), which produces CF_3_CHO in essentially 100% yield. CF_3_CHO has three competing fates in the atmosphere. First, it undergoes photolysis (annually averaged diurnal atmospheric lifetime in the troposphere of ≤ 2 days at 40° latitude) giving CF_3_ and HCO radicals [210] (see also Sect. 3.7). Second, oxidation initiated by OH produces acyl peroxy radicals, which can react with HO_2_, NO, or NO_2_. Reaction of these acyl peroxy radicals with HO_2_ radicals can lead to the formation of TFA as a minor product. Third, contact with liquid water produces hydrates, which can react with OH radicals leading to the formation of TFA [211]. The latter two processes are currently thought to be minor fates of CF_3_CHO. The reaction of the hydrate with OH radicals is an efficient pathway for generating TFA; however, the importance of hydrolysis of CF_3_CHO to give TFA is uncertain (see Sect. 3.7.3). Due to these competing fates, an estimated 2–30% of atmospheric CF_3_CHO is converted into TFA (see SI Sect. 4).

### Contribution of chemicals under the purview of the Montreal Protocol to the global load of trifluoroacetic acid

**Fig. 12:**
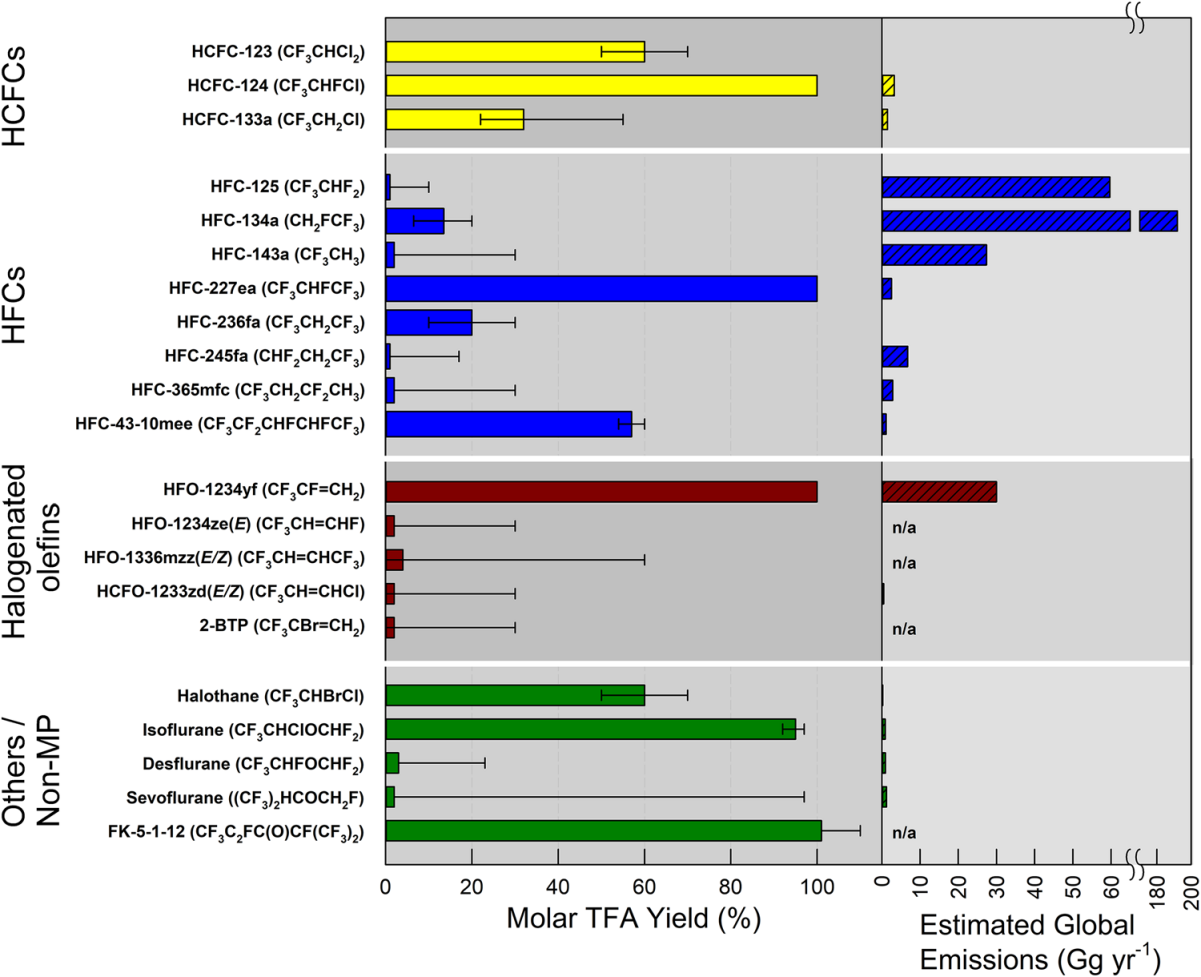
Yields of TFA from selected individual chlorofluorocarbon (CFC) replacement compounds, and their estimated global emissions. Also included are selected compounds not under the purview of the Montreal Protocol and Amendments. Error bars represent both experimental uncertainties and upper and lower yield ranges due to competing reaction channels that depend on environmental conditions. Yields of TFA from individual compounds are estimated based on evaluations of the available literature as described in Online Resource SI Sect. 4. Note split scale for the emission for HFC-134a, which is much higher than those of other compounds

Several CFC replacements give rise to the formation of TFA as an atmospheric oxidation product. Figure 12 provides an overview of estimated molar yields of TFA (%) for CFC replacements, as well as selected chemicals not under the purview of the Montreal Protocol (non-MP). In addition, some replacements such as HCFC-225ca (CF_3_CF_2_CHCl_2_) yield longer-chain PFCAs, CF_2_CF_2_COOH (100%). HFC-134a and HFO-1234yf are the two substitutes that have the largest predicted contribution to global TFA concentrations among those gases that fall under the purview of the Montreal Protocol. The Science Assessment Panel (SAP) and the Technology and Economic Assessment Panel (TEAP) of the Montreal Protocol under the United Nations Environment Program (UNEP) have projected future uses and potential releases from 2020 to 2100 for these two substitutes. A summary of the projected yield of TFA from degradation in the troposphere (Fig. 12) is provided in Table 3.

**Table 3:** Projected global yields of TFA from HFC-134a and HFO-1234yf and total deposition between 2020 and 2100

	HFC-134a	HFO-1234yf	Sum
Annual formation of TFA (a. e., acid equivalents)			
2020	0.01–0.03 Tg year^–1^	0.03–0.03 Tg year^–1^	0.04–0.06 Tg year^–1^
2050	0.02–0.05 Tg year^–1^	0.34–0.49 Tg year^–1^	0.36–0.54 Tg year^–1^
2100	0.01–0.02 Tg year^–1^	0.63–1.03 Tg year^–1^	0.64–1.05 Tg year^–1^
Sums of deposited TFA (a. e.)			
2020–2050	0.5–1.5 Tg	5.3–6.6 Tg	5.8–8.1 Tg
2020–2100	1.0–2.9 Tg	30.5–49.0 Tg	31.5–51.9 Tg
Concentration of TFA as the sodium salt in the oceans in		2050	244–246 ng L^−1^
		2100	266–284 ng L^−1^

These data are taken from Table 7.3 of the 2022 report of the Science Assessment Panel [212] and currently are best estimates for the two listed refrigerants. Releases of other potential sources of TFA (see Fig. 12) have not been included but are expected to be much smaller. Estimated future concentration in the oceans is based on the nominal value of 200 ng a.e. L^−1^ in 2020 and a total volume of 1.36 × 10^9^ km^−3^. For comparison to toxicity values, concentrations have been converted to sodium salt

These amounts of TFA are estimated to increase concentrations in the global oceans from the nominal value of 200 ng a.e. L^−1^ (equivalent to 239 ng TFA sodium salt L^−1^) estimated by Frank et al. [213] to 266–284 ng sodium salt L^−1^ in 2100 if evenly distributed across all oceans. If the actual concentrations were less than the nominal value (239 ng TFA sodium salt L^−1^), the predicted values for 2100 would be smaller. In a recent study in Germany, the contribution of currently used refrigerants to the formation of TFA was estimated [214]. The worst-case annual formation of TFA from refrigerant R134a was estimated at 1050 tons year^−1^, 1170 tons year^−1^ from refrigerant R1234yf, and 141 tons year^−1^ from all other refrigerants. If the proportions of TFA from other refrigerants in Germany are applied to the global estimate of deposition, the maximum value for contributions from all refrigerants would be about 6% greater, i.e., 302 ng sodium salt L^−1^.

It should be noted that the geographic distribution of TFA released into the atmosphere across the globe has changed with the introduction of refrigerants and blowing agents such as HFOs with short atmospheric lifetimes (days). The longer atmospheric lifetimes of the older generation HFCs allowed wider and more even distribution of parent HFCs and deposition of TFA, across the globe [215–217]. The HFOs will be degraded by tropospheric OH radicals closer to the source of release with resulting steeper gradients of concentration depending on wind direction and velocity [see examples of modeling of deposition in [217]. As a result of this uneven deposition, concentrations of TFA in surface waters will vary with flow rates and volumes of water. Prediction of concentrations of TFA in surface waters will require the development of hydrologic models, such as those now used to model distribution and concentrations of other pollutants in water. These types of models are available from the USEPA [218] but would need to be modified for modeling of the dispersion of HFOs and TFA once it reaches the surface.

### Trifluoroacetic acid in precipitation 

**Fig. 13:**
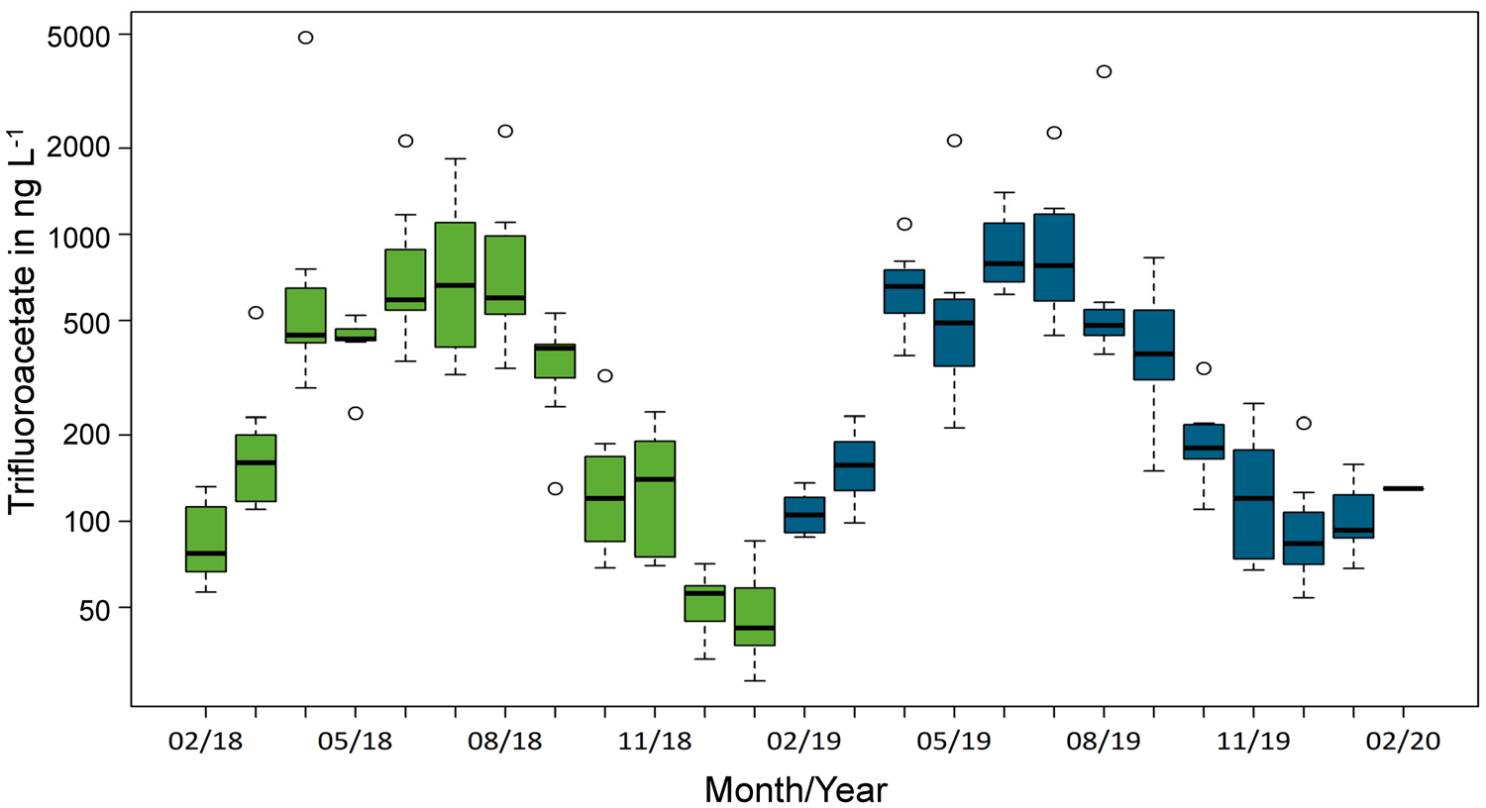
Concentration of trifluoroacetate in composite precipitation samples from eight sites in Germany from February 2018 to February 2020. The y-axis is on a binary logarithmic scale (log_2_) and the solid horizontal bar is the median, the box indicates the upper and lower quartiles, the whiskers the upper of lower quartile – or + the interquartile range × 1.5, and the data symbols are the outliers. From Fig. 27 in [220]

The presence of TFA in precipitation continues to be studied, with several new reports published since the last Quadrennial Assessment [6] Unless otherwise stated, only those studies with complete descriptions of analytical methods have been included. Analysis of rainwater samples collected in 2016 in 28 cities across China showed detectable amounts of TFA in all samples [219]. Concentrations ranged from a low of 9.1 to a high of 320 ng TFA a.e. L^−1^ and fluxes from 160 to 16,000 ng TFA a.e. m^−2^ day^−1^ (Tables SI-7 and SI-8 in [219]). A study on concentrations of TFA present in rainwater samples in eight locations across Germany from February 2018 to 2019 showed a seasonal range of concentrations over 1 year [220]. Across all sites, frequency of detection was greater than 90% except in December, January, and February. The greatest median concentration, 703 ng TFA a.e. L^−1^, was in June 2018. Over the year, daily fluxes across collection sites showed less variability with the greatest median flux of 774 ng TFA a.e. m^−2^ day^−1^ and the smallest of about 205 ng m^−2^ day^−1^ [Table 2 in [220]. This study was continued for an additional year [214] and a similar pattern was observed (Fig. 13). The source of the TFA was most likely degradation of fluorinated gases in the troposphere and the authors suggest that the seasonality is because of seasonal changes in solar UV radiation and the photochemical formation of OH radicals responsible for production of TFA in the troposphere [220]. Fluxes of TFA in rainwater in Germany [220] were less than those reported from China [219], probably because of the release of more precursors in greater concentrations in the latter location. A recent study on temporal trends in concentrations of TFA in surface waters reported increases in concentrations of sixfold between samples collected in 1998 and those collected in 2021 [221]. Concentrations in samples collected down-wind from the San Francisco Bay area were greater in 2021 (up to 2790 ng L^−1^) than in 1998 (up to 287 ng L^−1^). The author suggests that these residues of TFA are from the breakdown of fluorinated refrigerants, but fluxes were not reported so the role of reduced precipitation in generating the greater concentrations is unknown. Once reaching the surface, TFA will form salts with alkali metals and mix with surface- or interstitial-water in the soil. These salts can be taken up by plants (see below) and accumulate in plant tissues, particularly leaves. Based on this property, archived samples of various leaves of some species of trees from the German Environmental Specimen Bank were analyzed for the presence of TFA [222]. The leaves collected spanned the period from 1989 to 2020 and showed an increase in concentrations of TFA. For example, concentrations of TFA in leaves of Lombardy poplar increased from *ca*. 160 µg kg^−1^ (d.w.) in 1991 to ca 970 µg kg^−1^ in 2019. The authors suggest that the sources of TFA are replacements for the CFCs, mostly from precipitation. The authors are likely correct in this conclusion.

Breakdown products of some fluorinated chemicals include hydrofluoric acid (HF) and TFA, which are strong acids. However, the amounts generated from the oxidation of HFOs represent only a small (< 0.5%) contribution to the formation of acid rain in comparison to other sources such as sulfur and nitrogen oxides [223] and this is judged to not be of concern.

### Other sources of trifluoroacetic acid in the global environment

In previous assessments [6, 224, 225], we have discussed other potential sources of TFA that are not related to the chemicals under the purview of the Montreal Protocol. In the global context, there is a paucity of information on these sources, but they fall into some general groupings, *inter alia*:

- Geogenic sources
- Effluents and releases from the manufacture of fluorinated chemicals, including chemicals under the purview of the Montreal Protocol and Amendments
- Combustion and degradation of fluorinated chemicals in commercial and household waste
- Biological and environmental degradation of chemicals such as pharmaceuticals and pesticides that contain fluorine atoms, specifically the -CF_3_ moiety.

These are discussed in more detail below.

#### Geogenic sources

Since the early reports of the widespread presence of TFA in marine waters [213, 226] and its association with ^14^C-dated deep waters older than 1000 years [227], it was believed that there are natural sources of TFA. This is consistent with the report that concentrations of TFA increase with proximity to locations of undersea volcanic vents [227, 228].

However, the theory that TFA can be formed from geogenic sources has been challenged [229]. This challenge was partially based on potential analytical errors and lack of information on levels of detection and quantitation, and high variance in concentrations measured in samples at different depths and in different oceanic basins. The authors focused on atmospheric sources of TFA in surface waters and ice, which originates in precipitation and did not consider measurements in other bodies of water such as endorheic lakes and playas located in areas of low precipitation and little fluorochemical industry. One of these locations, the Dead Sea, had a reported concentration of 6400 ng L^−1^ [226]. The Dead Sea is in a rift valley with a history of geological faulting and with a volume of 114 km^3^, so that this concentration is equivalent to 730 tons of TFA. That this amount of TFA (measured in the 1990s) is all from anthropogenic sources is very unlikely, and geogenic sources are more plausible.

Another argument put forward that TFA does not originate from geogenic sources is the lack of TFA in older (> 2000-year-old) samples of glacial melt, surface, and ground water. These older waters originated from precipitation from evaporated (distilled) marine and surface waters. Like chloride, TFA from oceans (and possible endorheic lakes) could be carried to land close to the shore. However, this transport would only be for short distances and is unlikely to be a significant source of TFA and other PFAS in precipitation and/or ice cores [220]. While it is possible that TFA could have been released from surficial volcanos, these potential sources lack the combinations of high temperature and high pressures found in thermal vents in the deep ocean. It should be noted that some authors have reported the presence of fluorinated and/or chlorinated short-chain and aromatic carbon compounds (but not TFA) in emissions from surficial volcanos [reviewed in [230, 231].

The background value of 200 ng TFA L^−1^ in the oceans as suggested by Frank et al. [213] would be equivalent to 268 × 10^6^ tons of TFA in the global oceans if well mixed globally. However, based on analyses in several oceanic basins, lesser amounts (a range of 61–205 × 10^6^ tons equivalent to 45–152 ng L^−1^) were suggested by Scott et al. [227]. From the total known use and release of HFC-134a, HFC-143a, and HFC-227ea between 1990 and 2015, the total amount of TFA that could theoretically have been produced is 4.5 × 10^6^ tons. This is very much less than the total based on the range of concentrations measured in the oceans, which, using an estimated ten-fold range, would be equivalent to 27–270 × 10^6^ tons. Even with this assumption, this is equivalent to a discrepancy of 6 to 60-fold that is much larger than would be explainable by anthropogenic activity in relation to use of the HFCs. This gap is most likely from natural sources.

In the absence of more rigorous and consistent sampling of the oceans, these concentrations and amounts are speculative. For the purposes of comparisons to toxicity values and risk assessment, the EEAP [6, 224, 225, 232] used the larger value to err on the side of caution when estimating further contribution from the chemicals under the purview of the Montreal Protocol to the total load in the global oceans. Another major unknown in characterizing the source of reported concentrations of TFA in the oceans is the degradation half-life of TFA in the environment. As discussed above, TFA is very recalcitrant and is essentially unreactive under normal environmental conditions. If, as seems to be the case, the half-life is likely very long (≈ centuries), very small amounts could accumulate over time to explain the amounts observed in oceans and endorheic basins.

#### Manufacturing of fluorinated chemicals 

In the 1990s, there was only one manufacturer of TFA in the USA [201] and relatively few manufacturers of fluorinated refrigerants that fall under the purview of the Montreal Protocol. Since that time, the manufacture of fluorinated chemicals has increased, and these facilities are found in many countries around the world. Details on the amounts produced, use, and release of most of these chemicals and by-products are not reported in a way that is accessible to the public or the scientific community such as it is for chemicals under the purview of the Montreal Protocol. Several recent papers have reported measurements of TFA and potential precursors in locations near manufacturing facilities. Here, we focus mostly on publications in the last 4 years.

A study of leachates and effluents from municipal waste disposal sites and landfills in the environs of Tianjin (China), the location of several fluorochemical manufacturing facilities, showed the presence of many PFAS as well as TFA at all sampling sites [233]. Greatest amounts of TFA (60,000 and 50,000 ng a.e. L^−1^) were measured in leachates from two incineration plants, although leachates from one landfill and one transfer-site had similarly high concentrations approaching 40,000 ng a.e. L^−1^. TFA was detected in samples of surface water and soils near a fluorochemical complex in Jinan (China) [234]. Concentration of TFA in lotic (flowing) surface waters ranged from 300 to 1000 ng L^−1^ but two lentic (still water sites) had mean concentrations of 1700 and 2600 ng a.e. L^−1^. Well- and tap-water had concentrations of about 250 ng a.e. L^−1^. In the same study, concentrations of TFA in soil samples close to factories ranged from 59 to 2081 ng a.e. kg^−1^. Uptake of TFA from nutrient solution (containing 2,000,000,000 ng a.e. L^−1^) by roots and leaves of wheat plants resulted in accumulated concentrations 170,000,000 ng kg^−1^ in roots and 100,000,000 ng kg^−1^ in shoots after exposure for 80 h [235]. Given the large and unrealistic concentrations in the nutrient solution, this is not surprising. Other PFAS were also taken up but to a lesser extent than TFA. This study did demonstrate uptake of TFA by plants. As discussed in the previous update report [225], maize and poplar plants take up TFA from contaminated soils [236]. Transport to the leaves was greater than to the stalk or the maize kernels. This is consistent with transport with water to the sites of transpiration and loss of water vapor through the leaves, resulting in accumulation of non-volatile salts of TFA. The median bioaccumulation factor (soil:leaf) was about 200. When these authors fed maize leaves to herbivores (locusts) the leaf-to-locust transfer factors were < 1 (0.028–0.185), indicative of negative trophic magnification (trophic dilution), most likely because locusts can excrete TFA, as has been demonstrated in mammals [201]. The large concentrations of TFA found in the environments around factories and plants manufacturing fluorinated chemicals indicate large fugitive emissions from some facilities that manufacture these chemicals. Given the small number of studies, the total load to the environment is very uncertain but it is an issue that needs to be addressed regionally, even if it is outside the scope of the Montreal Protocol.

#### Combustion and thermolysis of fluorinated chemicals

Polymers containing fluorine, such as polytetrafuoroethylene (PTFE) and related products, are heavily used in urban and industrial areas [237]. Data on amounts of fluoropolymers produced each year are not easily obtained; median estimates of value are in the region of 8 billion US dollars; however, there were no data on the mass of the products. When these polymers are subjected to high temperatures, they can degrade to yield TFA or precursors of TFA [238]. When heated to 500 °C in the presence of air, PTFE, polychlorotrifluoroethylene (CPTFE), ethylene chlorotrifluoroethylene (ECTFE), and polytetrafluoroethylene-co-tetrafluoroethylene perfluoropropylether (PFEPE) yielded 7.8, 9.5, 6.3, and 2.5% TFA, respectively [238]. A similar study on this source of TFA in Beijing (China) indicated yields of TFA from thermolysis of PTFE, poly(vinylidene fluoride-hexafluoropropylene) (PVDF-HFP), and poly(vinylidene fluoride-co-chlorotrifluoroethylene) (PVDF-CTFE) were 1.2%, 0.9% and 0.3%, respectively, which was estimated to contribute 0.6–6.1 ng a.e. L^−1^ to precipitation over this city [239]. These are potential sources of TFA to the environment but little information was found in the literature on the effect of conditions of combustion (ranging from very high incineration temperature of waste to open-burning) on the rates of formation of TFA. However, this does remain a possible, but globally uncertain, source of TFA. A recent laboratory study of degradation of PFCAs [240], has shown that exposure of PFCAs (see Table 2) to sodium hydroxide in a polar aprotic solvent (e.g., dimethyl sulfoxide) resulted in degradation to fluoride ions in yields between 78 and 100% in 24 h. TFA was formed in amounts of 19–39 mol% for PFCAs with 5–9 carbon chains. The authors suggest that this observation might lead to the development of methods for disposing of PFCAs. However, the TFA produced in the process might become a source of TFA to the environment.

#### Unidentified sources of exposure to trifluoroacetic acid 

A review of the global occurrence of PFCs in water from wastewater treatment plants identified only two studies (included in previous reports from the EEAP) that had reported the presence of TFA [241]. Whether the TFA was formed during treatment of the wastewater or was present in the incoming effluent could not be determined; however, the authors speculated that it could have been formed from degradation of longer-chain PFAS precursors.

In a study of the concentrations of PFAS in the serum of staff and support workers in Nankai University in Tianjin (China), a location where fluorochemicals are manufactured, TFA and other PFAS were detected [242]. The frequency of detection of TFA was 97% but 12 other PFAS had greater frequencies of detection. The median concentration of TFA in serum of the volunteers was 8460 ng L^−1^ and the 75^th^ centile was 12,550 ng a.e. L^−1^. Given the high solubility in water as noted above (and low K_ow_, Table 2), this concentration is likely equivalent to a systemic burden of the same values in ng kg^−1^. These values are 4.4 orders of magnitude less than the NOED (No Observed Effect Dose) for TFA in rats (discussed below) and do not suggest biologically significant risks for humans. Concentrations of PFOS and PFOA were greater than TFA and these chemicals are more toxic than TFA and are retained in the body for longer periods (Table 2). The authors reported an association between the sum of the concentrations of PFAS and biomarkers of diabetes but offered no insight as to causality by a specific chemical or the route of exposure to these chemicals.

Residues of TFA have been found in beverages such as beer and herbal infusions (teas) [243]. Analysis of samples of beer from 23 countries spanning the globe provided a range of concentrations with a median of 6100 ng L^−1^ and a maximum of 51,000 ng a.e. L^−1^. The authors opined that the source of TFA in the beer and teas was not the water used to make the beverage, suggesting rather that the barley or the hops and the dried leaf of the tea(s) was the source of the contamination. Measurements of TFA in barley have not yet been reported in the literature but uptake of TFA from soil into maize kernels (discussed above) resulted in accumulations with a range of 40,400 to 102,000 ng a.e. kg^−1^ [236]. If accumulation in barley is like maize, this is a possible explanation; however, the source of the TFA in the barley is uncertain. It could originate from industrial sources of contamination or pesticides used in agriculture that break down to produce TFA and a terminal residue (see below).

An earlier paper from China [244] had reported the detection of many PFAS in outdoor dust; however, they did not analyze for TFA. Residues of TFA (and other PFAS) have now been detected in indoor and outdoor dust in China [245]. Median concentrations of TFA in outdoor dust from six locations in China ranged from 61,000 to 222,000 µg a.e. kg^−1^ with no consistent differences between rural and urban sites. In urban sites, concentrations of TFA in indoor dust from six locations in China ranged from 117,000 to 470,000 µg a.e. kg^−1^. Concentrations of other PFAS were much smaller [245]. Using procedures from the USEPA, these authors also estimated daily intake values of PFAS of toddlers and adults that could result from ingestion of dust. The 95th centile estimated daily intakes of TFA for toddlers and adults were 5.3 and 0.55 ng a.e. kg^−1^ body weight, respectively, for indoor dust. The 95th centile estimated daily intakes for toddlers and adults from outdoor dust were 3.2 and 0.33 ng a.e. kg^−1^ body weight. The original source(s) of the contamination in the dusts are unknown but there were amounts of unknown precursors (37 − 67 mol %) for PFAS in the dust [245].

#### Pharmaceuticals and pesticides

Fluorine atoms are frequently added to pharmaceuticals and pesticides to enhance or modify their biological properties. The most common use is replacement of a hydrogen with a fluorine atom. The van der Waals radii for hydrogen (0.12 nm) and fluorine (0.14 nm) are similar and small compared to that of other halogens such as chlorine (0.18 nm) [246]. Thus, fluorine-substituted chemicals are more likely to successfully dock with receptor sites than chemicals substituted with larger halogens such as Cl. The C-F bond is one of the strongest in organic chemistry, so this substitution tends to make the molecule more resistant to biochemical breakdown, which prolongs biological activity. In addition, substitution of hydrogen with fluorine can change other properties of the chemical, especially of adjacent chemical groups. For example, the F atom is a much stronger withdrawer of electrons than an H atom. As compared to hydrogen with a Hammett sigma value of zero, the Hammett for a single fluorine substituent on a benzene ring is + 0.062 for para-effect and + 0.337 for the meta-affect. For a -CF_3_ on a benzene ring, the Hammett sigma values are + 0.54 and + 0.43, respectively.

For comparison with the precursors of TFA that are under the purview of the Montreal Protocol, the following discussion is focused on chemicals with a C–CF_3_ moieties since these could potentially degrade to produce TFA as a terminal residue. It is estimated that about 20% of pharmaceuticals currently in commerce contain one or more fluorine atoms. As of 2020, the number of pharmaceuticals containing fluorine atom(s) was 369 [247] and in 2021, 13 new products containing fluorine were added [248]. Of these, 77 contain C–CF_3_ moieties [from Fig. 4 in [247]]. Some bacteria use fluoxetine (Prozac®, CAS# 54,910-89-3) as a sole source of carbon and the terminal metabolite is TFA [249], which is not further metabolized. These authors also reported photolytic defluorination of intermediates formed in the degradation of fluoxetine [Fig. 7 in [249]] but did not specify intensity or wavelength. Whether this photolytic defluorination occurs under environmental conditions is unknown. TFA can also be formed from some fluoro-pharmaceuticals during treatment of water with O_3_. The molar yield of TFA from fluoxetine solutions treated with O_3_ at 4 mg L^−1^ was as large as 40% [202].

Pharmaceuticals are used globally but there is a paucity of information on the amounts produced and used in the treatment of humans and other animals. However, these pharmaceuticals and/or their breakdown products are excreted and, thus, enter the environment. For example, anesthetic procedures involving halothane (CF_3_CHClBr), isoflurane (CF_3_CHClOCHF_2_), and desfluorane (CF_3_CHFOCHF_2_) are known to produce TFA as a metabolite in humans. Anesthesia using halothane can result in significant levels of TFA in blood (20,000–110,000 µg L^−1^), which is excreted in urine [250–253]. Those pharmaceuticals that contain the C–CF_3_ moiety are expected to be a source of TFA in the environment but there are two unknowns, the yield of TFA from the breakdown of the pharmaceutical and the amounts of pharmaceuticals that are used. More publicly available information is needed to even begin to address this uncertainty. To estimate the contribution of pharmaceuticals to the total global load of TFA would be highly speculative but they are a potential source of TFA.

Some pesticides also contain one or more C–CF_3_ moieties [254] and are potential sources of TFA in the environment [225]. Breakdown of some of these pesticides to TFA has been investigated. For example, ozonation (4 mg L^−1^) of solutions of trembotrione (mesotrione, CAS# 104,206-82-8), flufenacet (CAS# 142,459-58-3), flurtamone (CAS# 96,525-23-4), and fluopyram (CAS# 658,066–35-4) at 100 µg L^−1^ for times between 5 and 60 min resulted in 5, 20, 43, and 32% production of TFA on a molar basis [Fig. 7 in 202]. Whether ozonation is a good model for the formation of TFA from pesticides in agricultural soils or not is unknown. The yield of TFA from the degradation of pesticides is dependent on the other substituents on the molecule and the environmental conditions. Studies on the photolysis of the lampricide 3-trifluoromethyl-4-nitrophenol (TFM CAS# 88–30-2) used to control the sea lamprey in the North American Great Lakes have shown that photolysis (365 nm) results in the formation of TFA [255]. Yields were dependent on the pH of the solution and ranged from 5 to 18%. Conversion of the nitro-group to an amino-group increased the rate of conversion but not the yield. In another study, yields of TFA from photolysis of the penoxsulam (an herbicide) and sulfoxaflor (an insecticide) exposed to UV radiation in river water were less than 5% under laboratory conditions [256]. Neither of these pesticides were included in Table 4. Yields of TFA from penoxsulam were greater in river water than in distilled water at pH 7 (Fig. SI-18 in [256]). This dependence of the formation of TFA on environmental conditions and substituents on the other parts of the molecule likely applies to other pesticides and to pharmaceuticals and other potential precursors of TFA.

**Table 4:** Potential release of TFA from fluorinated-pesticides used in the USA from 1992 to 2018

Name^a^	MW^a^	Formula^a^	Number of C-CF_3_ moieties^a^	Molar yield of TFA	Estimated tons of TFA
Acifluorfen	361.657	C_14_H_7_ClF_3_NO_5_	1	0.315	3332
Bicyclopyrone	399.366	C_19_H_20_F_3_NO_5_	1	0.286	117
Bifenthrin	422.872	C_23_H_22_ClF_3_O_2_	1	0.270	2116
Chlorfenapyr	407.615	C_15_H_11_BrClF_3_N_2_O	1	0.280	315
Cyflumetofen	447.454	C_24_H_24_F_3_NO_4_	1	0.255	28
Cyhalothrin-gamma	449.854	C_23_H_19_ClF_3_NO_3_	1	0.253	73
Cyhalothrin-lambda	449.854	C_23_H_19_ClF_3_NO_3_	1	0.253	1431
Dithiopyr	401.41	C_15_H_16_F_5_NO_2_S_2_	1	0.284	1
Ethalfluralin	333.267	C_13_H_14_F_3_N_3_O_4_	1	0.342	11,437
Fipronil	437.141	C_12_H_4_Cl_2_F_6_N_4_OS	2	0.522	809
Flonicamid	229.162	C_9_H_6_F_3_N_3_O	1	0.498	168
Fluazifop	327.259	C_15_H_12_F_3_NO_4_	1	0.348	1887
Fluazinam	465.089	C_13_H_4_Cl_2_F_6_N_4_O_4_	2	0.490	407
Flubendiamide	682.392	C_23_H_22_F_7_IN_2_O_4_S	2	0.334	233
Flucarbazone	396.297	C_12_H_11_F_3_N_4_O_6_S	1	0.288	105
Flufenacet	363.331	C_14_H_13_F_4_N_3_O_2_S	1	0.314	2623
Flumetralin	421.733	C_16_H_12_ClF_4_N_3_O_4_	1	0.270	306
Fluometuron	232.206	C_10_H_11_F_3_N_2_O	1	0.491	12,618
Fluopicolide	383.576	C_14_H_8_Cl_3_F_3_N_2_O	1	0.297	30
Fluopyram	396.717	C_16_H_11_ClF_6_N_2_O	2	0.575	292
Fluridone	329.322	C_19_H_14_F_3_NO	1	0.346	27
Flutolanil	323.315	C_17_H_16_F_3_NO_2_	1	0.353	1229
Fluvalinate	502.918	C_26_H_22_ClF_3_N_2O3_	1	0.227	4
Fomesafen	438.758	C_15_H_10_ClF_3_N_2_O_6_S	1	0.260	6519
Isoxaflutole	359.319	C_15_H_12_F_3_NO_4_S	1	0.317	1495
Lactofen	461.774	C_19_H_15_ClF_3_NO_7_	1	0.247	1120
Mesotrione (trembotrione)	440.814	C_17_H_16_ClF_3_O_6_S	1	0.259	4565
Novaluron	492.706	C_17_H_9_ClF_8_N_2_O_4_	1	0.231	221
Oxyfluorfen	361.701	C_15_H_11_ClF_3_NO_4_	1	0.315	3117
Prosulfuron	419.379	C_15_H_16_F_3_N_5_O_4_S	1	0.272	151
Saflufenacil	500.85	C_17_H_17_ClF_4_N_4_O_5_S	1	0.228	525
Tefluthrin	418.736	C_17_H_14_ClF_7_O_2_	1	0.272	1893
Thiazopyr	396.376	C_16_H_17_F_5_N_2_O_2_S	1	0.288	7
Trifloxystrobin	408.377	C_20_H_19_F_3_N_2_O_4_	1	0.279	1492
Trifluralin	335.283	C_13_H_16_F_3_N_3_O_4_	1	0.340	65,327
Triflusulfuron	478.403	C_16_H_17_F_3_N_6_O_6_S	1	0.238	30
Total					122,604

^a^Data from [254]. Data on use of pesticides in the United States are from [257]. See Online Resource Table 2 for annual quantities

Similarly, as for pharmaceuticals, the amounts of pesticides used across the globe are not known at the level of individual chemicals. The Food and Agricultural Organization of the United Nations collects data on pesticide use by country, but these data are grouped by chemical class of pesticide and information on individual products is not available. However, data on annual estimates of the use of individual pesticides are available in the United States through the database on Estimated Annual Agricultural Pesticide Use, maintained by the US Geological Survey through the National Water-Quality Assessment Project [257].

To obtain a better understanding of the possible contributions of pesticides to the global load of TFA, we estimated the use of those pesticides containing one or more C-CF_3_ moieties from data available in the United States. Maps and associated estimates of amount of pesticide applied [257] were downloaded and then compiled. As a worst case, upper estimates of use were selected and all data from 1992 to 2018 were collected, summed, and converted to tons of active ingredient. The molar yield of TFA was calculated using the ratio of the molecular weight of TFA (114.02 Daltons) and the pesticide (from Table S3 in [254]). This is a conservative assumption as the actual yield of TFA is dependent on other substituents on the molecule and the primary driver(s) of degradation. The potential total tonnage of TFA released from these pesticides was then calculated. These results are shown in Table 4.

It should be noted that these estimated values are based on worst-case assumption of highest estimated use and complete conversion of all C-CF_3_ moieties in the chemical to TFA. Global use of pesticides in 2019 was estimated as 4,190,985 tons with 495,475 tons in North America [258], approximately 12% of the global use. Because of selection for resistance and the availability of alternatives, some of the pesticides included in Table 4 are no longer in use and the use-pattern of those currently in use will likely change in the future. For this reason and the lack of data on global pesticide use, the estimates of total global contribution to loads of TFA are uncertain; however, in comparison to future loads of TFA resulting from the release of chemicals under the purview of the Montreal Protocol, the potential contribution from pesticides is small.

**Fig. 14:**
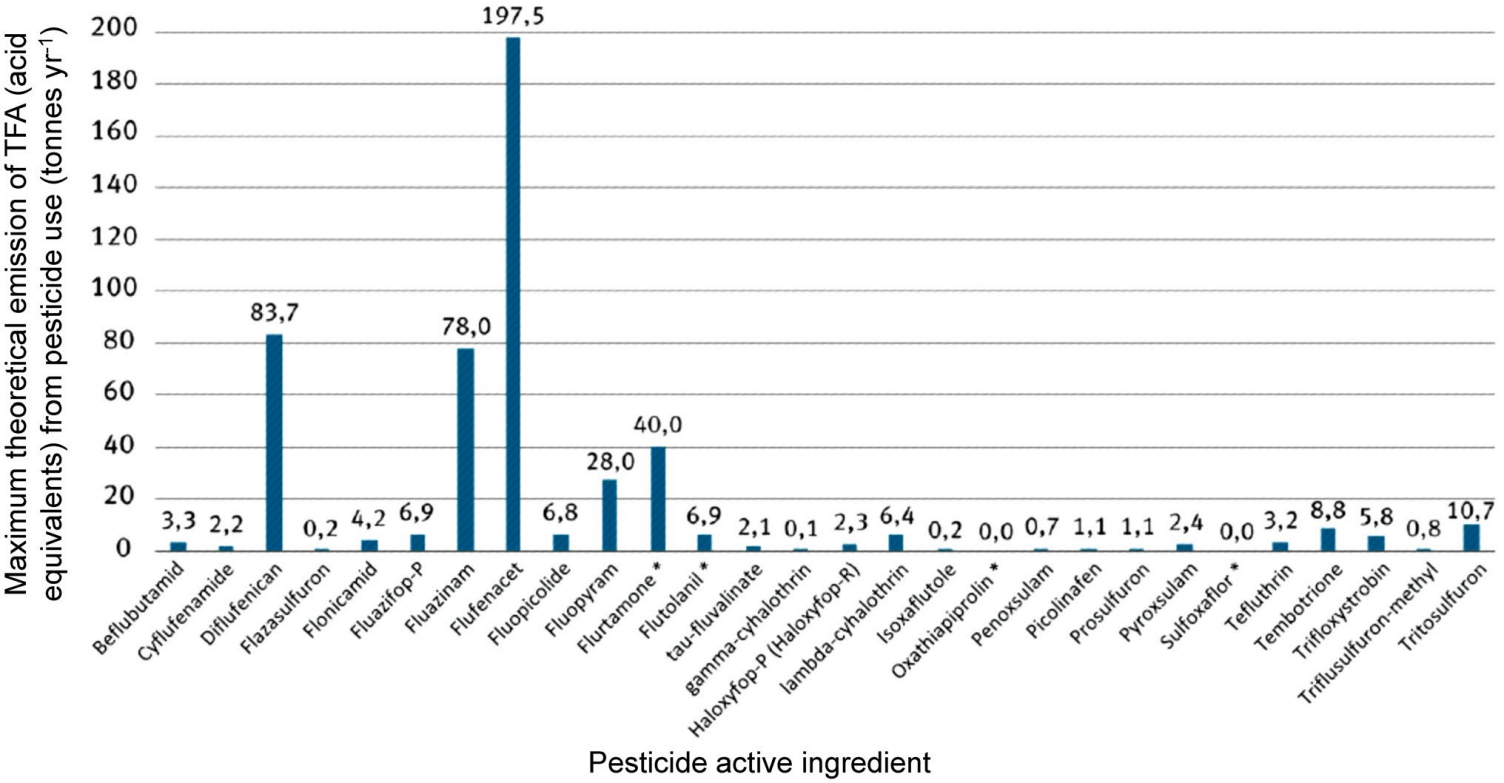
Maximum possible annual emissions of TFA from plant protection products in Germany for active ingredients that can theoretically form TFA. Data based on mean sales volume of each of the active substances over the three years 2016, 2017 and 2018 (Figure from [259])

A recent analysis of sources of TFA in the environment from the German Environment Agency [214] reported on precursors of TFA and included uses of refrigerants gases and other uses of chemicals for 2016–2018. Most of the TFA was estimated to be sourced from five products. The potential annual production of TFA from use of pesticides in Germany was 504 tons per year (Fig. 14), based on a mean of 3 years) but should not be compared directly with the data from the United States (Table 4), which presents total estimated use over 26 years.

### Human and environmental risks associated with trifluoroacetic acid in the environment 

#### Risks from exposure to TFA in terrestrial animals

Since the last Quadrennial Assessment, only one report on the potential effects of TFA in mammals was found in the literature [260]. This was a report on the effects of exposure to TFA salts via drinking water in male laboratory rats. The tests followed OECD guidelines (Tests 417 and 452 [261]) and exposures were for 90, 370, and 412 days at concentrations in the drinking water of 0 (control), 30, 120, and 600 mg TFA a.e. L^−1^. The latter concentration was equivalent to a daily intake of 37.8 mg TFA kg^−1^ (b.m.). Responses measured were activity of the enzymes alanine-amino-transferase (ALT) and glutamate-pyruvate-transferase (GPT) in the blood. No significant effects were observed at 30 mg L^−1^ for any time of exposure but a significant increase in ALT activity was observed at 120 and 600 mg TFA a.e. L^−1^ at 370 days but not at 412 days. No effects on GPT were reported. In a second study with exposures for 14, 28, and 90 days to 0, 600, 1200, and 2400 mg TFA a.e. L^−1^, no significant effects on ALT were observed. Increases in the activity of enzymes in or originating from the liver are considered as compensatory unless accompanied by physiological responses such as loss of weight. This reported effect does not change the conclusion that TFA is of low toxicity in mammals.

An extensive review of the potential effects of TFA in the environment published by the German Environmental Agency [214] did not identify any risks other than the persistence of TFA in the environment, which is a legislative rather than toxicological criterion. The concentrations of TFA in beer and tea discussed above are small when compared to the NOED of TFA in mammals. This indicates that the risk to humans from residues of TFA in beer and tea are *de minimis* (of little importance).

#### Risks of exposure to TFA in aquatic organisms 

**Fig. 15:**
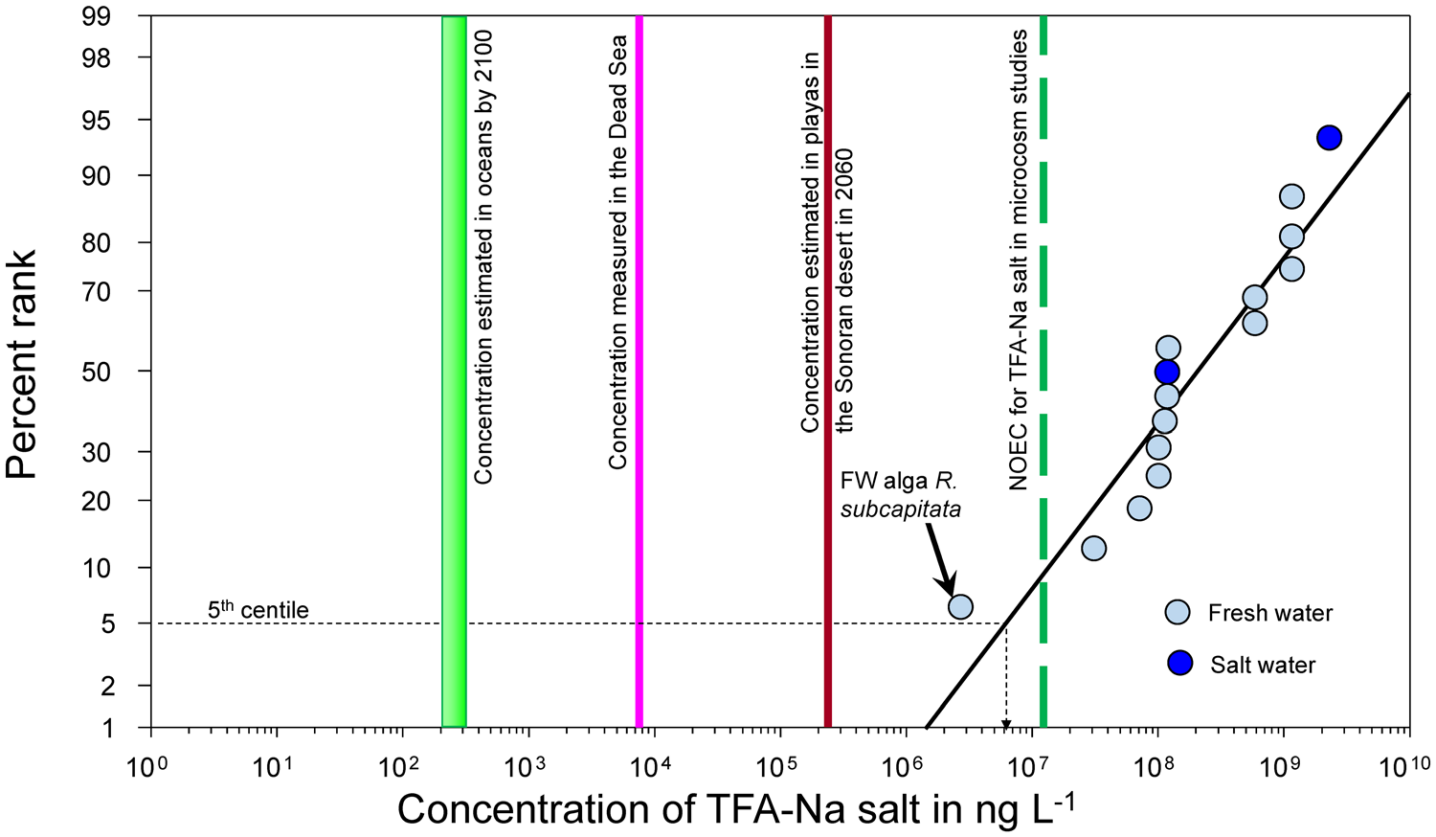
A log-probability cumulative frequency plot of no observed effect concentrations (NOEC) of trifluoroacetic acid salt compared to various environmental concentrations in water. The dashed vertical green line indicates the NOEC for TFA-Na salt in microcosms is a toxicology-based criterion

Since the last Quadrennial Assessment, one new toxicity test for an aquatic organism was located. This was a retest of the most sensitive alga (*Raphidocelis subcapitata*) conducted on behalf of Solvay [192]. The study protocol followed OECD guideline 201 [261]. Effect values were based on growth. A no observed effect concentration (NOEC) of 2.5 mg a.e. L^−1^ (2,500,000 ng L^−1^) was reported based on inhibition of growth. Being a more recent study conducted under OECD guidelines, this data point was substituted for the older (1999) study used in the 2016 risk assessment [232] and is illustrated in Fig. 15. Although this new study on *R. subcapitata* has not been published in the literature, the study was conducted under Good Laboratory Practice Guidelines with Quality Assurance and Quality Control [262]. The detailed report of the study was reviewed by ECHA [263] and was classified as “*reliable without restriction*”, hence it has been used here in the characterization of the toxicity of TFA to aquatic organisms.

The margin of exposure between the distribution of NOECs and the observed and expected concentrations in the oceans and endorheic basins is several orders of magnitude and is indicative of *de minimis* risk.

#### Environmental persistence of TFA as an assessment criterion

Environmental persistence is one of the criteria used to identify persistent organic pollutants (POPs), such as those regulated under the purview of the Stockholm convention. Persistence alone has been suggested to be a criterion for regulatory action [264]. The suggested threshold for this classification is a degradation half-life > 6 months. The stability of TFA and its salts indicates a half-life >> 6 months, but our opinion is that persistence should only be considered as a regulatory criterion for substances that are moderately or highly toxic and/or are bioaccumulative in organisms and/or undergo trophic magnification. TFA does not bioaccumulate nor is it toxic at the low to moderate exposures currently measured in the environment or those predicted in the distant future.

### Other issues relevant to the degradation of fluorinated chemicals and release of TFA

#### Potential effects of hydrofluorocarbons and hydrofluoroolefins on tropospheric ozone

The photochemistry of CFC replacements, including HFCs and halogenated olefins, could impact air quality through formation of tropospheric O_3_ on urban or regional scales. The spatial and temporal variation in VOC emissions, non-linear chemistry of the reacting chemical species, and complex atmospheric mixing and transport factors, all contribute to large uncertainties and thus complicate this assessment. In the past, e.g., [265], the potential impact has been addressed based on the indices of either the photochemical ozone creation potential (POCP) [266] or the Maximum Incremental Reactivity (MIR) index [267]. The MIR index values reflect the mass (grams) of ozone formed relative to the mass (grams) of VOC emitted. The POCP is a relative potential determined from the effect of a small incremental increase in the emission of a chemical on the calculated amount of O_3_ formed, relative to the effect of an identical mass emission of ethene as a reference chemical. The former was developed with a focus on ozone formation in urban plumes and mainly in a United States urban-scale context. The latter addresses ground-level ozone formation on the regional, multi-day episode scale, as it most often occurs in Europe, here being predominantly a long-range transboundary and transport air quality issue. Both indices require fully speciated emission inventories and non-trivial model calculations. However, the POCP values can also be estimated to a first approximation based on structure and reactivity of the VOC, which is especially useful for VOCs for which no full chemical mechanisms have been implemented in atmospheric model studies, i.e., for many of the HFCs and HFOs discussed here.

With the exception of a 3D global modeling study (Geos Chem) of the impact of HCFO-1233zd(E) [215], there have been no new reports of POCPs/MIR values for the HFCs and HFOs/HCFOs in the literature over the last review period. Recently, a methodological update to the original POCP estimation method has become available [268] (see Online Resource, SI Sect. 3 for details). Table SI 3 (Online Resource) lists the updated POCP values for CFCs and their replacements, based on the most recent reactivity evaluations and estimated for both north-west European and United States urban reference conditions. These values now provide index values on the same comparable scale and can be used as an approximate indicator of the potential impact on tropospheric ozone from the CFC replacements. In general, first- and second-generation CFC replacements, HCFCs and HFCs, have very small POCP values. Many of the common HFOs, with some exceptions, have POCP values that lie between those for methane (0.57/0.22) and ethane (10.91/4.52). The POCP values for HFOs are generally larger than those for the analogous HFCs, but much smaller than those for the parent alkenes. This is consistent with the few explicit MIR modeling studies of HFOs available in the literature. e.g., [267, 269]. It is clear from these studies, and from the values in Online Resource SI Table 3, that substitution of e.g., HFC-134a emissions for an equal mass of HFO-1234yf emissions, would lead to an increase in POCP-weighted emissions (two orders of magnitude). Still, MIR studies and atmospheric modeling studies of HFO-1234yf, have shown that O_3_ production from HFO-1234yf is indistinguishable from that from ethane (also consistent with Online Resource Table 3), and that replacing HFC-134a in vehicle air conditioning units with HFO-1234yf across the United States has a negligible impact (< 0.01%) on the formation of tropospheric ozone. It is clear from the above, that the small increases in tropospheric ozone formation generated from a transition from HFC emissions to emissions of HFOs would not be of concern.

#### Relevance of trifluoroacetaldehyde, a precursor of TFA, is increasing

With the transition from HFCs to third-generation alternatives such as HFO-1234ze(E) and HCFO-1233zd(E), the atmospheric abundance of CF_3_CHO, (estimated 40° latitude, annually averaged diurnal tropospheric lifetime of ≤ 2 days [210]) is expected to increase in source regions. Ambient concentrations of HFO-1234ze and HCFO-1233zd(E) have been measured in central Europe (Germany) at urban, semi-urban, and remote sites from 2011 onwards [270–272]. As discussed in Sect. 3.2, these chemicals give CF_3_CHO as an intermediary product of atmospheric degradation, with a yield of 100%. In 2020 the measured mean abundances of HFO-1234ze and HCFO-1233zd(E) in central Europe at Jungfraujoch (remote, 3580 m above sea level) were 0.98 ng/m^3^ (0.21 pptv) and 1.01 ng/m^3^ (0.19 pptv), respectively. This is an increase from 0.18 ng/m^3^ (0.039 pptv) and 0.02 ng m^−3^ (0.003 pptv) in 2013 at this same measurement station. A recent 3D global chemistry and transport model study [273] suggests significant increases in the abundance of CF_3_CHO in source regions, as well as in the global background; however, it appears that the model of Wang et al. does not include photolysis of CF_3_CHO, which is the major tropospheric sink for CF_3_CHO (see above). They employed a high and a low emissions scenario for HFO-1234ze (12.6 or 124.4 ktonne year^−1^ by 2050) and predicted annual average mixing ratios of 11 ng m^−3^ (2.7 pptv) for CF_3_CHO in China (source region) and 0.7 ng m^−3^ (0.18 pptv) as a global average for their low emissions scenario. For their high emissions scenario, they predict annual mixing ratios in China of 413 ng m^−3^ (103 pptv). These values are all significantly larger than those (< 5 pg m^−3 ^level) which can be inferred from a 3D global chemistry model study of the atmospheric degradation of HCFO-1233zd(E) with contemporary emissions estimates (0.5 Gg year^−1^) [215]. The stark difference in the two studies can be explained by the difference in CF_3_CHO chemistry employed by the two models. Due to the fast photolysis of CF_3_CHO, concentrations of CF_3_CHO are unlikely to build up to ng m^−3^ levels, locally or globally.

#### Sinks for CF_3_CHO: a photolytic source of HFC-23 and CF_3_CHO-hydrate formation

The dominant overall sink for CF_3_CHO is thought to be photolysis [210]. The photolysis can proceed through three principal pathways:

(1a) CF_3_CHO + h*ν* → CF_3_ + HCO,

(1b) CF_3_CHO + h*ν* → CF_3_H + CO,

(1c) CF_3_CHO + h*ν* → CF_3_CO + H.

Chiappero et al. [210] reported a quantum yield of Φ_1a_ = 0.17 ± 0.03 and no indication of reaction 1b occurring in the 308 nm photolysis of CF_3_CHO. However, a recent study by Campbell et al. reported that reaction 1b has a quantum yield at 308 nm of Φ_1b_ = 0.01 ± 0.005, and that in effect 11 ± 5.5% of CF_3_CHO would undergo reaction via reaction 1b in the atmosphere to yield CF_3_H (HFC-23) [274]. CF_3_H is a strong GHG (GWP = 12,690), and its photochemical formation through reaction 1b could, in effect, present an additional and potentially significant contribution to the radiative forcing of climate of the parent CFC alternatives. The study of Campbell et al. 2020 [274] was based on an indirect technique using CO as a marker for pathway 1b and conducted at very low, sub-atmospheric pressure and temperatures. A subsequent chamber study at atmospheric pressures by Sulbaek Andersen and Nielsen [275] using broad-band actinic radiation shows no formation of CF_3_H in the tropospheric photolysis of CF_3_CHO and an estimated upper limit for the yield of CF_3_H of 0.3% was established. Whereas the study of Sulbaek Andersen and Nielsen [275] was carried out under tropospheric conditions, none of the past photolytic studies were performed using the same photolysis sources and detection methods. As suggested by Sulbaek Andersen and Nielsen [275], investigations of the photolysis at wavelengths relevant to the upper troposphere/lower stratosphere would be of interest for a more comprehensive modeling of the atmospheric photolysis of CF_3_CHO.

The yield of TFA from CF_3_CHO may depend on whether it remains in gaseous form. It has been reported that formaldehyde is efficiently converted to gaseous formic acid via a multiphase pathway involving a hydrated form of formaldehyde [276]. Earlier, using computational methods, Rayne and Forest [277] suggested that CF_3_CHO will be dominantly present as the hydrated form in aqueous solution. The reaction of OH radicals with the hydrated form of CF_3_CHO in the gas phase is known to be an effective route for formation of TFA (100%) [211]; however, to what extent CF_3_CHO could be removed from the atmosphere through wet scavenging and undergo multiphase chemistry is unknown.

#### CF_3_CHO and TFA: Interactions with particle growth and formation of new particles

Recent simulations have shown that both CF_3_CHO and TFA can participate in particle growth and particle formation processes [278–280]. However, these processes are highly dependent on season and atmospheric conditions. The reaction of CF_3_CHO/(CH_3_)_2_NH/H_2_O can compete well as a sink for CF_3_CHO at night (when photolysis is not occurring and concentrations of OH radical are low) if relative humidity and dimethylamine concentrations are high.

TFA enhances the formation rate of new dimethylamine/sulfuric particles significantly (up to 227-fold [278]), but only under conditions of relatively low temperatures and sulfuric acid concentrations, and relatively high TFA and dimethylamine concentration. With increasingly effective regulations on emissions of sulfur-containing pollutants, the enhancement of new particle formation by TFA may become increasingly important in urban areas where emissions of sulfur are expected to decline.

#### Potential impact of reactions of TFA with stabilized Criegee intermediates

Stabilized Criegee intermediates (SCI), highly reactive chemicals formed in the atmosphere, react rapidly with PFCAs including TFA, producing hydroperoxyfluoroesters. This is likely the dominant gas-phase fate of PFCAs [281], although this only constitutes a temporary reservoir. SCIs exist in the greatest concentrations over forested regions, where emissions of biogenic alkene are high. The lifetime of TFA would be as short as 2 days over land areas with significant SCI-mediated loss, such as in tropical forests [216]. However, hydrolysis of the ester products, and reaction with OH, simply regenerates TFA; therefore, reactions of TFA with SCI are unlikely to have a substantial impact on the overall loss of gas-phase TFA [281]. In effect, the SCI-mediated oxidation products were found to have no significant impact on concentrations or distributions of TFA in the atmosphere [216] and this is not a pathway for net destruction of TFA in the environment.

### Conclusions and uncertainties

TFA is a perfluorinated acid that has been included in the class of per- and poly-fluoroalkyl substances (PFAS). This class of chemicals contains 4730 substances, of which about 256 are in commercial use. Even in the subclass of perfluorinated alkanoic acids, the physical, chemical, and biological properties of these substances differ widely, mostly in relation to length of the alkyl chain. To regulate these substances as a class (as has been suggested) is not scientifically defensible and TFA should be treated as a unique chemical for the purposes of regulation.

TFA is an acid when formed in the atmosphere but on reaching the surface (soil or water) it forms salts with alkali metals (e.g., sodium, potassium, calcium, etc.). Because of its lack of reactivity, TFA salts are persistent in the environment and estimates of half-life are uncertain but could be in the range of centuries or millennia. This persistence is not a major concern because it does not react with biomolecules. TFA and its salts are easily excreted by animals and do not bioaccumulate in food chains. Salts of TFA have low toxicity to animals and plants and there are very wide margins between current/projected exposures and toxicity values.

One source of TFA in the environment is the degradation of replacements for chemicals that contribute to the destruction of stratospheric O_3_. These are the HCFCs, HFCs, and HFOs, all of which are replacements for chemicals that fall under the purview of the Montreal Protocol. Some of these are greenhouse gases and contribute to global climate change. Because of this, there is a trend to replace long-lived HCFCs and HFCs with HFOs, which have very short atmospheric lifespans and do not contribute to climate change. The use of these replacements is monitored under the auspices of the Montreal Protocol and estimates of current and future releases of TFA are regularly assessed. These releases will add to the existing load of TFA in the environment but predicted amounts are well below the threshold for concern with respect to human and environmental health.

Initial reactions in the atmospheric degradation of HCFCs, HFCs, and HFOs that lead to TFA are well understood. Some uncertainties still exist for the atmospheric fate of CF_3_CHO. With the transition from HFCs to HFOs, the importance of the degradation product, CF_3_CHO, in the environment is increasing. Nevertheless, CF_3_CHO is likely to be only a minor source of TFA. Other than HCFCs, HFCs, and HFOs, there are additional sources of TFA in the environment. TFA is used as a laboratory reagent and is the starting material for many industrial products. It is formed from combustion of fluoropolymers and as a terminal breakdown product of fluorinated pharmaceuticals and pesticides. Fugitive releases have resulted in high levels of contamination near manufacturing facilities and TFA is routinely detected in surface waters. Other than precipitation, which contains TFA formed in the atmosphere, the sources for TFA in surface waters are uncertain. However, preliminary estimates of possible releases from use of pesticides in the United States and Germany suggest that total amounts are less than those from HCFCs, HFCs, and HFOs. Amounts released from degradation of pharmaceuticals are very uncertain and amounts from fugitive releases from manufacturing are completely unknown.

TFA released into the environment will eventually collect in terminal basins such as endorheic lakes or the oceans. Because the HCFCs and HFCs are long-lived in the atmosphere, they distribute globally and TFA from these substances is more evenly deposited. The HFOs and HCFOs have shorter lifetimes in the atmosphere and deposition of TFA from these substances is likely to be more localized. This will result in greater concentrations near the locations of release. This is unlikely to present a risk to humans or the environment in these locations but changes in concentration in surface water (or soil) would respond rapidly to releases. Monitoring of the environment for residues of TFA would provide an early warning if trends in concentration indicate rapid increases.

Presence of TFA in precipitation and flowing waters will be driven by release from precursors and other sources as well as the hydrology and will likely fluctuate. Concentrations in terminal basins such as the oceans will fluctuate less but will be dependent on rates of inputs from precipitation and rivers. Once in the oceans, concentrations will be influenced by rates of input of fresh water as well as currents and mixing in the oceans. Current and projected (to 2100) concentrations of TFA in the oceans provide a very large margin of exposure (thousand-fold) when compared to thresholds of toxicity, and risks to the environment and human health are *de minimis*. However, there is some uncertainty in the environmental toxicity values because only two marine species are included in the toxicity data set for aquatic species; and marine macrophytes, which are keystone species with respect to habitat, have not been tested.

There are several national and regional programs that monitor and report on concentrations of chemicals such as pesticides in surface water. For example, the National Water-Quality Assessment program in the United States [257] and NAIADES program in France [282]. These programs have the infrastructure to routinely sample flowing waters from many watersheds and analyze these for residues of pesticides. It should not be difficult or costly to include TFA in these analyses. If started soon, these data could be useful in characterizing inputs of TFA from the short-lived HFOs as well as in identifying point-sources of industrial inputs.

## Knowledge gaps

There have been significant efforts to improve air quality through reductions in VOCs and NOx emissions. However, major uncertainties remain for future emissions scenarios, as well as changes in environmental conditions such as UV-B radiation, temperature, and humidity, which are sensitive to changes in stratospheric O_3_ and climate. Furthermore, models need to be improved to better characterize the production and destruction of ground-level O_3_ in complex urban VOC-NOx mixtures, the propensity for producing SOA, and the contribution of the transport of stratospheric O_3_ to the troposphere.

Knowledge of global OH concentrations and their variability must be enhanced to constrain the estimated atmospheric lifetime of the many gases removed by OH. This will require improved assessment of anthropogenic and natural emissions of species that control the concentration of atmospheric OH, including CO, methane, and NOx. Better methods are needed to estimate global-scale OH concentrations, since the use of methyl chloroform for this purpose has become less reliable due to its rapidly decreasing emissions.

Some uncertainties remain in our understanding of the sources, routes of formation, and environmental fate of TFA. Identification and quantification of potential natural sources of TFA are urgently needed. Similarly, better local/regional emission inventories are needed for short-lived precursors of TFA arising from CFC replacements, as well as characterization of all other anthropogenic sources of TFA. In addition, reliable estimates of yields of TFA are required for current and new replacement compounds and their partially oxidized degradation intermediates. Finally, there is some uncertainty in toxicity values for TFA because of the limited number of marine species tested.

## Overall conclusions

UV-B radiation has positive and negative impacts on tropospheric air quality. UV-B radiation is essential to the formation of photochemical smog, including ground-level O_3_, particulate matter, but is also essential for removing pollutants from the atmosphere. Thus, changes in UV-B radiation have consequences for both air quality and the lifetime of many gases, including some GHGs and VSLSs. Poor air quality remains a major health problem globally, despite progress in reducing anthropogenic emissions of air pollutants. The Montreal Protocol has prevented large increases in UV-B radiation; however, interactions with climate change complicate predictions. Future changes in UV-B radiation are uncertain but present substantial dangers given the widespread vulnerability of humans to, e.g., photochemical smog.

The Montreal Protocol has led to the replacement of ODSs with fluorinated chemicals, some of which can undergo degradation in the atmosphere to give TFA in various yields. TFA is known to have a long environmental lifetime and accumulates in surface and ground waters. At present, there are large uncertainties associated with the concentrations of TFA in various environmental compartments in some regions, as well as the relative proportion of anthropogenic sources related to the Montreal Protocol, compared to the other anthropogenic and natural sources. There is some uncertainty in toxicity values because of the limited number of marine species tested. Current and predicted concentrations (to year 2100) of TFA in the oceans provide a large margin of exposure (thousand-fold) when compared to thresholds of toxicity.

The topics of this assessment align closely with several of the Sustainable Development Goals (SDGs) [283]. Issues in air-quality (Sect. 2) specifically relate to SDG 3 (*Good Health and Well-being*), but also inform SDG 2 (*Zero Hunger—*via* damage to crops*), SDG 11 (*Sustainable Cities and Communities—*via* impacts on livability*), and SDG 13 (*Climate Action*—via OH controlling the lifetimes of many climate-relevant gases). Atmospheric processing of CFC replacements leading to persistent chemicals such as TFA (Sect. 3) raises concerns about SDG 12 (*Responsible Consumption and Production*) and in the context of SDG 6 (*Clean Water and Sanitation*—via accumulation in sources of drinking water).

## Supplementary Information

Below is the link to the electronic supplementary material.

Supplementary file1 (PDF 757 KB)

## Data Availability

All data generated or analyzed are included or published previously.
