# Supplementary material for: Changes in tropospheric air quality related to the protection of stratospheric ozone in a changing climate
Source: Photochem Photobiol Sci. 2023 Jun 13:1–48. Online ahead of print. doi: 10.1007/s43630-023-00369-6 (PMC10262938; doi:10.1007/s43630-023-00369-6)
Supplement: Supplementary file 1 — Supplementary file1 (PDF 757 KB) [file 43630_2023_369_MOESM1_ESM.pdf]

Supplementary information

Changes in tropospheric air quality related to the protection of stratospheric ozone in a changing climate

S. Madronich[1,2], B. Sulzberger[3], J. D. Longstreth[4], T. Schikowski[5], M. P. Sulbæk Andersen[6], K. R. Solomon[7], and S. R. Wilson[8]

1 National Center for Atmospheric Research, Boulder, USA.
2 USDA UV-B Monitoring and Research Program, Natural Resource Ecology Laboratory, Colorado State University, Fort Collins, USA.
3 Emerita from Eawag, Swiss Federal Institute of Aquatic Science and Technology, CH-8600Duebendorf, Switzerland.
4 The Institute for Global Risk Research, LLC, Bethesda, USA.
5 Research Group of Environmental Epidemiology, Leibniz Institute of Environmental Medicine, Dusseldorf, Germany.
6 Department of Chemistry and Biochemistry, California State University, Northridge, USA.
7 School of Environmental Sciences, University of Guelph, Guelph, Canada.
8 School of Earth, Atmospheric and Life Sciences, University of Wollongong, Wollongong, Australia.

Corresponding authors: S. Madronich (sasha@ucar.edu); S.R. Wilson (swilson@uow.edu.au)

SI 1 Physical, chemical, and biological properties of the linear perfluorinated carboxylic acids

SI Table 1. Physical, chemical, and biological properties of the linear perfluorinated carboxylic acids from 2-8 carbons.

**Property**	**Trifluoroacetic acid**	**Perfluoro-propanonic acid**	**Perfluoro-butanoic acid**	**Perfluoro-pentanoic acid**	**Perfluoro-hexanoic acid**	**Perfluoro-heptanoic acid**	**Perfluoro-octanoic acid**
Abbreviation	TFA	PFPrA	PFBA	PFPeA	PFHxA	PFHpA	PFOA
CAS#	76-05-1	422-64-0	375-22-4	2706-90-3	307-24-4	375-85-9	335-67-1
Molecular formula	CF_3COOH	CF_3CF_2COOH	$CF_3(CF_2)_2COOH$	$CF_3(CF_2)_3COOH$	$CF_3(CF_2)_4COOH$	$CF_3(CF_2)_5COOH$	$CF_3(CF_2)_6COOH$
# of C atoms	2	3	4	5	6	7	8
Molecular weight	114.02	164.03	214.04	264.05	314.05	414.0	464.08
BP (°C)	73	96.5	121.0	140 [a]	157	188-192	189 [f]
Solubility (H_2O mg L^{-1})	Miscible	Miscible	Miscible	112,600	21,700	3400–9500	9500
Vapor pressure (Pa)	11	3.93 [a]	1307 [a]	1057	263	1.77 [a]	1.72 [a]
Log K_{OW}	0.5	1.5 [a]	2.43 [a]	3.262 [a]	3.48	5.024 [a]	5.905 [a]
Henry's Law Constant (atm m^{-3} mol^{-1})	1.11×10^{-7}	4.43×10^{-6} [a]	0.0051 [a]	0.029 [a]	0.174 [a]	1.521 [a]	3.044 [a]

SI Table 1. Physical, chemical, and biological properties of the linear perfluorinated carboxylic acids from 2-8 carbons.

**Property**	**Trifluoroacetic acid**	**Perfluoro-propanonic acid**	**Perfluoro-butanoic acid**	**Perfluoro-pentanoic acid**	**Perfluoro-hexanoic acid**	**Perfluoro-heptanoic acid**	**Perfluoro-octanoic acid**
pKa	0.3	0.38 [a]	-0.2–0.7	-0.06	-0.13	-0.15	-0.16–3.8
Koc (L kg^{-1})	0.17–20	12.7 [a]	58 [a]	270 [a]	1247 [a]	5761	30,440
Acute oral toxicity in rat (mg kg^{-1})	> 500	> 750	NA	NA	1750–5000 [b]	NA	NA
NOEL (rat mg kg^{-1})	114 [b]	NA	3.01	NA	15–30 [b]	20 [c]	1 [c]
NOEL Repro. (rat, mg kg^{-1})	NA	NA	50	NA	35 [j]	100 [c]	0.1 [c]
NOEC most sensitive aquatic plant (ng L^{-1})	2.5×10^6 [d]	1.44×10^7 [e]	6.21×10^8 [e]	> 1.00×10^9 [e]	NA	> 1.02×10^9 [e]	5.80×10^3 [e]
NOEC most sensitive aquatic animal (ng L^{-1})	LC50 = 7×10^7 [e]	LC50 = 8.0×10^7 [f]	LC50 = 1.1×10^8 [f]	LC50 = 1.3×10^8 [f]	LC50 = 1.4×10^8 [f]	LC50 > 1.02×10^6 [f]	LC50 = 1.5×10^8
Half-life in rats [g]	34–48 h [i]	NA	1–9 h	NA	2–5 h	1.4–2.4 h	44–322 h
Half-life in humans [g]	16 h	NA	72–81 h	NA	14–49 d [j]	1.2–1.5 y	2.1–10 y

Unless otherwise stated, references are from (PubChem, 2022). Other sources are: [a] (RSC, 2022) [b] (NICNAS, 2016) [c] (EFSA, 2020) [d] (Chabot, 2017) [e] (Boudreau, 2002) [f] (Wang et al., 2014) [g] (ATSDR, 2021) [h] (Boutonnet et al., 1999).

SI Table 2. Annual usage of pesticides containing one or more trifluoromethyl moieties in the USA from 1992 to 2018.

Name	MW	Formula	#C-CF3	Molar Yield TFA	Molar Yield TFA-Na salt	Multiplier in lb	1992	1993	1994	1995	1996	1997	1998	1999	2000	2001	2002	2003	2004	2005	2006	2007	2008	2009	2010	2011	2012	2013	2014	2015	2016	2017	2018	TFA tonnes	TFA-Na tonnes
acifluorfen	361.657	$C_{14}H_7ClF_3NO_5$	1	0.315	0.376	1000000	1.5	1.4	2.1	1.6	2.2	1.7	1	0.7	0.7	0.7	0.5	0.4	0.4	0.4	0.3	0.2	0.2	0.4	0.3	0.3	0.3	0.8	1.2	1	1.1	1	1.3	3332	3975
bicyclopyrone	399.366	$C_{19}H_{20}F_3NO_5$	1	0.286	0.341	1000000																								0	0.2	0.3	0.4	117	139
bifenthrin	422.872	$C_{23}H_{22}ClF_3O_2$	1	0.270	0.322	1000000	0.2	0.2	0.2	0.2	0.2	0.2	0.2	0.2	0.2	0.2	0.4	0.4	0.4	0.3	0.2	0.4	0.5	0.8	0.8	0.8	1.6	1.6	1.5	1.3	1.4	1.7	1.8	2116	2524
chlorfenapyr	407.615	$C_{15}H_{11}BrClF_3N_2O$	1	0.280	0.334	1000000				0.1	1.4	0.1	0.9	0	0																			315	376
cyflumetofen	447.454	$C_{24}H_{24}F_3NO_4$	1	0.255	0.304	1000																							1	40	70	65	66	28	33
cyhalothrin-gamma	449.854	$C_{23}H_{19}ClF_3NO_3$	1	0.253	0.302	1000000													0	0	0	0	0	0	0	0	0.1	0.1	0.1	0.1	0	0.1	0.1	73	88
cyhalothrin-lambda	449.854	$C_{23}H_{19}ClF_3NO_3$	1	0.253	0.302	1000000	0.2	0.2	0.1	0.3	0.3	0.3	0.4	0.3	0.3	0.3	0.4	0.3	0.3	0.4	0.4	0.4	0.6	0.6	0.6	0.6	0.6	0.8	0.8	0.8	0.8	0.8	0.9	1431	1707
dithiopyr	401.41	$C_{15}H_{16}F_5NO_2S_2$	1	0.284	0.339	1000																	0.1	0.1	0.3	0.2	0.3	0.5	0.8	1.1	1.8	2.2	1.7	1	1
ethalfluralin	333.267	$C_{13}H_{14}F_3N_3O_4$	1	0.342	0.408	1000000	4.8	4	4.6	5.1	4.2	3.2	4.6	4.5	3	2.8	2.2	2.7	2.7	2	1.8	1.5	1.5	1.7	1.6	1.2	1.5	1.4	1.6	1.7	1.4	4	2.4	11437	13643
fipronil	437.141	$C_{12}H_4Cl_2F_6N_4OS$	2	0.522	0.622	1000000							0.2	0.2	0.3	0.4	0.4	0.2	0.3	0.4	0.1	0.2	0.1	0.2	0.2	0.1	0	0	0	0	0	0	0	809	965
flonicamid	229.162	$C_9H_6F_3N_3O$	1	0.498	0.594	1000000															0	0	0	0	0.1	0.1	0.1	0.1	0.1	0.1	0.1	0.1	0.1	168	201
fluazifop	327.259	$C_{15}H_{12}F_3NO_4$	1	0.348	0.416	1000000	0.8	0.9	0.9	1.1	1	0.1	0.8	0.6	0.6	0.5	0.3	0.4	0.3	0.2	0.3	0.3	0.2	0.4	0.3	0.1	0.2	0.2	0.2	0.4	0.4	0.2	0.4	1887	2251
fluazinam	465.089	$C_{13}H_4Cl_2F_6N_4O_4$	2	0.490	0.585	1000000									0	0	0	0.1	0.2	0.1	0.1	0	0.1	0.1	0.1	0.1	0.1	0.1	0.1	0.2	0.1	0.2	0.2	407	485
flubendiamide	682.392	$C_{23}H_{22}F_7IN_2O_4S$	2	0.334	0.399	1000000																		0.1	0	0.1	0.3	0.3	0.2	0.3	0.2	0.1	0	233	278
flucarbazone	396.297	$C_{12}H_{11}F_3N_4O_6S$	1	0.288	0.343	1000										10	18	19	42	35	60	30	62	33	35	55	82	54	58	55	41	32	80	105	125
flufenacet	363.331	$C_{14}H_{13}F_4N_3O_2S$	1	0.314	0.374	1000000							0.9	1.2	1.5	1.4	1.3	1.4	1.5	1.3	0.9	1.3	0.9	1.4	0.7	0.4	0.8	0.3	0.1	0.9	0.1	0	0.6	2623	3129
flumetralin	421.733	$C_{16}H_{12}ClF_4N_3O_4$	1	0.270	0.323	1000000	0.1	0.1	0.1	0.2	0.1	0.1	0.1	0.1	0.1	0.1	0.1	0.1	0.1	0.1	0.1	0.1	0.1	0.1	0.1	0.1	0.1	0.1	0.2	0.1	0.1	0.1	0.1	306	365
fluometuron	232.206	$C_{10}H_{11}F_3N_2O$	1	0.491	0.586	1000000	4.7	4.9	6	7.2	5.8	4.9	4.9	3.2	1	0.6	1.1	0.7	0.8	0.6	0.6	0.7	0.5	0.5	0.8	1.1	0.8	0.6	0.7	0.8	1	1	1.2	12618	15051
fluopicolide	383.576	$C_{14}H_8Cl_3F_3N_2O$	1	0.297	0.355	1000																	3	7	8	17	26	31	29	31	19	22	32	30	36
fluopyram	396.717	$C_{16}H_{11}ClF_6N_2O$	2	0.575	0.686	1000000																					0	0.1	0.1	0.2	0.2	0.3	0.3	292	348
fluridone	329.322	$C_{19}H_{14}F_3NO$	1	0.346	0.413	1000			42																					29	56	24	24	27	33
flutolanil	323.315	$C_{17}H_{16}F_3NO_2$	1	0.353	0.421	1000000				0.1	0.2	0.1	0.1	0.1	0.1	0.1	0.2	0.2	0.2	0.2	0.1	0.2	0.1	0.3	0.4	0.5	0.6	0.4	0.6	0.9	0.7	0.9	0.7	1229	1465
fluvalinate	502.918	$C_{26}H_{22}ClF_3N_2O_3$	1	0.227	0.270	1000	5.7	1.8	2.5	6.5	2.6	1.7	1.5	1.5	1.4	1.4	1.2	0.8	0.7	0.6	0.6	0.5	0.6	0.6	0.5	0.5	0.5	0.7	0.8	0.8	0.9	0.8	0.7	4	5
fomesafen	438.758	$C_{15}H_{10}ClF_3N_2O_6S$	1	0.260	0.310	1000000	0.4	0.5	0.6	0.7	1.3	1.2	1.3	1.2	1.4	0.8	0.7	0.7	0.7	0.8	0.7	0.7	0.7	1.6	2	2.6	3	3	3.6	5.1	6.7	7	6.3	6519	7776
isoxaflutole	359.319	$C_{15}H_{12}F_3NO_4S$	1	0.317	0.379	1000000								0.3	0.3	0.4	0.5	0.5	0.5	0.5	0.5	0.4	0.3	0.4	0.4	0.6	0.7	0.7	0.8	0.7	0.7	0.7	0.7	1495	1784
lactofen	461.774	$C_{19}H_{15}ClF_3NO_7$	1	0.247	0.295	1000000	0.3	0.2	0.3	0.4	0.4	0.4	0.2	0.2	0.2	0.2	0.2	0.1	0.1	0.1	0.1	0.1	0.1	0.3	0.3	0.3	0.5	0.8	0.8	0.9	0.8	0.5	1.2	1120	1336
mesotrione (tembotrio	440.814	$C_{17}H_{16}ClF_3O_6S$	1	0.259	0.309	1000000										0	0.5	0.8	1.5	1.8	2	2.1	1.9	1.8	2	2.1	2.5	2.6	2.5	2.9	3.4	4.1	4.4	4565	5446
novaluron	492.706	$C_{17}H_9ClF_8N_2O_4$	1	0.231	0.276	1000000													0	0.1	0	0.1	0.1	0.1	0.1	0.1	0.1	0.1	0.1	0.1	0.9	0.1	0.1	221	263
oxyfluorfen	361.701	$C_{15}H_{11}ClF_3NO_4$	1	0.315	0.376	1000000	0.4	0.5	0.5	0.7	1.2	0.7	0.8	1	0.7	0.7	0.6	0.7	0.7	0.7	0.8	0.7	0.7	0.7	0.6	0.9	0.9	1.1	0.9	0.9	1	1.2	1.6	3117	3718
prosulfuron	419.379	$C_{15}H_{16}F_3N_5O_4S$	1	0.272	0.324	1000000				0	0.1	0.1	0.1	0.1	0.1	0.1	0.1	0.1	0.1	0.1	0	0.1	0	0	0	0	0	0.1	0	0	0	0	0	151	181
saflufenacil	500.85	$C_{17}H_{17}ClF_4N_4O_5S$	1	0.228	0.272	1000000																			0.2	0.3	0.4	0.5	0.6	0.8	0.7	0.8	0.8	525	626
tefluthrin	418.736	$C_{17}H_{14}ClF_7O_2$	1	0.272	0.325	1000000	0.4	0.4	0.5	0.5	0.9	0.8	0.8	0.6	0.7	0.6	0.8	1	0.8	0.8	0.8	0.7	0.6	0.4	0.3	0.3	0.5	0.6	0.5	0.4	0.4	0.3	0.2	1893	2259
thiazopyr	396.376	$C_{16}H_{17}F_5N_2O_2S$	1	0.288	0.343	1000						2	0	1.8	3.2	2.6	6.7	2.6	1.8	6.3	14	2.6	3.6	0	0	3	3.5	3						7	9
trifloxystrobin	408.377	$C_{20}H_{19}F_3N_2O_4$	1	0.279	0.333	1000000									0.1	0.1	0.1	0.2	0.2	0.3	0.3	0.4	0.5	0.8	0.6	0.7	1	1	1.8	1	0.8	1.2	0.9	1492	1780
trifluralin	335.283	$C_{13}H_{16}F_3N_3O_4$	1	0.340	0.406	1000000	27	28	31	29	28	27	23	23	21	14	13	13	12	11	11	11	11	7.5	8	8.5	10	8	12	11	8	10	8	65327	77926
triflusulfuron	478.403	$C_{16}H_{17}F_3N_6O_6S$	1	0.238	0.284	1000					15	22	22	19	17	20	23	17	19	23	18	30	10	2	3	3.5	1	1	1	2	2.5	2.5	1	30	35

US use data from https://water.usgs.gov/nawqa/pnsp/usage/maps/compound_listing.php
Upper estimate of use.

SI 3 Estimated photochemical ozone creation potentials (POCP$_{\varepsilon}$s) for ODSs, replacement compounds and related VOCs

The POCP estimation method employed here (Jenkin, 2017) is based on structure and reactivity for the species and different parameters for each geographical region (Northwest European or urban USA), and follows the equation $POCP_{\varepsilon} = (A \times \gamma_S \times R \times S \times F) + P + R_{O_3} - Q$, where A is a simple multiplier, γ_S describes the structure of the VOC, R is a OH reactivity element, S is a parameter related to the size of the VOC, and F is parameter that can account for the impact of unreactive carbonyl degradation products (set to 1 for all chemicals in SI Table 3). P applies only for chemicals that undergo photolysis at tropospheric conditions (set to 0 for all chemicals in Table 6) and R_{O_3} accounts for the formation of free radicals through the direct reaction of O_3 with the VOC. The latter is only included for the aliphatic alkenes in SI Table 3, as a) of the compounds in SI Table 3, only these compounds have significant reactivities towards O_3, b) the main OH formation mechanism requires at least one H atom attached to the carbon adjacent to the double bond – none of the HFO/HCFOs have this (Cox et al., 2020), and c) any additional OH formation routes (e.g., from excited CH_2OO radicals) are likely to be unimportant. Finally, Q applies only for a specific set of aromatic VOCs.

It is germane to note that the POCP estimation method was developed and tested for application on hydrocarbons, aliphatic olefins, and oxygenated VOCs. It was not developed explicitly cover halogenated compounds. Note also that the most recent POCP estimation equation published by Jenkin and co-workers (Jenkin, 2017) "*for alkenes and unsaturated oxygenates that react significantly with O₃*", contains an error in Eq. (9), which should correctly read "R_{O3} = E × m" (Jenkin, 2022). The values below are POCPs estimated for both North-West European and USA urban conditions, using updated OH radical kinetics.

SI Table 3. Atmospheric lifetimes, OH radical rate coefficients, estimated photochemical ozone creation potentials (POCPεs) for olefins and related chemicals, calculated for North-West European conditions and USA urban conditions. Unless otherwise noted, estimated total atmospheric lifetimes are those quoted in IPCC AR6 (Smith, 2021) and OH rate coefficients are from Burkholder et al. (Burkholder et al., 2020). POCPεs calculated using the (updated) approach by Jenkin et al. (Jenkin, 2022; Jenkin, 2017). See main text for details.

**Chemical**	**Industrial Designation**	**Total Atmospheric Lifetime**	**OH radical rate-coefficient,** (k_{OH}, cm^3 molecule^{-1}s^{-1}, 298 K, 1 atm)	**POCP$_{\varepsilon}$**, North-west European conditions (relative units)	**POCP$_{\varepsilon}$,** USA urban conditions (relative units)
Alkanes					
CH_4	*Methane*	11.8 years	6.3 × 10^{-15}	0.6	0.2
CH_3CH_3	*Ethane*	58 days	2.4 × 10^{-13}	10.9	4.5
Alkenes					
CH_2=CH_2	*Ethene*	1.5 days [a]	7.9 × 10^{-15}	100.4	98.6
CH_3CH=CH_2	*Propene*	0.4 days [a]	2.5 × 10^{-13}	110.6	134.5
CH_3CH_2CH=CH_2	*But-1-ene*	0.4 days [a]	3.2 × 10^{-15}	107.1	127.3

Chemical	Industrial Designation	Total Atmospheric Lifetime	OH radical rate-coefficient, (k_{OH}, cm^3 molecule^{-1}s^{-1}, 298 K, 1 atm)	POCP$_\varepsilon$, North-west European conditions (relative units)	POCP$_\varepsilon$, USA urban conditions (relative units)
ODSs					
CH_3CCl_3	*Methyl chloroform*	5 years	1.0×10^{-14}	0.11	0.04
$CHCl_2F$	*HCFC-21*	1.7 years	2.9×10^{-14}	0.38	0.16
$CHClF_2$	*HCFC-22*	11.9 years	4.0×10^{-15}	0.08	0.03
HFCs					
CHF3	*HFC-23*	228 years	3.0×10^{-16}	0.01	<0.01
CH_2F_2	*HFC-32*	5.4 years	1.0×10^{-14}	0.30	0.12
CF_3CHF_2	*HFC-125*	30 years	1.0×10^{-15}	0.02	0.01
CF_3CH_3	*HFC-143a*	3.6 years	1.0×10^{-15}	0.02	0.01
CF_3CH_2F	*HFC-134a*	14 years	4.0×10^{-15}	0.06	0.02
CHF_2CH_3	*HFC-152a*	1.6 years	3.0×10^{-14}	0.70	0.27
CF_3CHFCF_3	*HFC-227ea*	36 years	1.0×10^{-15}	0.01	<0.01
CF_3CH_2FCHF	*HFC-245eb*	3.2 years	1.0×10^{-14}	0.18	0.07
CHF_2CHFCF_3	*HFC-236ea*	11.4 years	5.2×10^{-15}	0.05	0.02
HFOs/HCFOs					
CF_2=CH_2	*HFO-1132a*	4.7 days	2.8×10^{-12}	19.8	15.6
CHF=CF_2	*HFO-1123*	1.5 days	8.1×10^{-12}	15.1	17.5
CH_2=CHF	*HFO-1141*	2.6 days	5.0×10^{-12}	38.0	33.6
CF_2=CF_2	*HFO-1114*	1.1 days	1.0×10^{-11}	8.69	11.3
CF_3CH=CH_2	*HFO-1243zf*	9.1 days	1.5×10^{-12}	11.2	6.56
CF_3CF=CH_2	*HFO-1234yf*	12 days	1.1×10^{-12}	7.32	4.23
CF_3CF=CF_2	*HFO-1216*	5.5 days	2.2×10^{-12}	4.65	4.16
Z-CF_3CF=CHF	*HFO-1225ye(Z)*	9.9 days	1.3×10^{-12}	5.91	3.92
E-CF_3CF=CHF	*HFO-1225ye(E)*	5.8 days	2.3×10^{-12}	7.25	5.81
E-CF_3CH=CHF	*HFO-1234ze(E)*	19 days	7.0×10^{-13}	5.60	2.88
Z-CF_3CH=CHF	*HFO-1234ze(Z)*	9.9 days	1.4×10^{-12} [c]	1.51	0.61
CF_3CF_2CH=CH_2	*HFO-1345zfc*	9 days	1.4×10^{-12}	6.96	3.96
E-CF_3CH=$CHCF_3$	*HFO-1336mzz(E)*	122 day	1.3×10^{-13}	0.98	0.39
Z-CF_3CH=$CHCF_3$	*HFO-1336mzz(Z)*	27 days	4.8×10^{-13}	2.90	1.35
E-CF_3CF=$CFCF_3$	*Octafluoro-2-butene*	31 days	5.8×10^{-13}	2.07	1.19
$(CF_3)_2C$=CH_2	*3,3,3-Trifluoro-2-(trifluoromethyl)-prop-1-ene*	10 days	7.8×10^{-13} [d]	4.02	2.08
E-$(CF_3)_2CFCH$=CHF	*HFO-1438ezy(E)*	43 days [b]	3.2×10^{-13}	1.55	0.65
E-CF_3CH=CHCl	*HCFO-1233zd(E)*	42 days	3.5×10^{-13}	0.55	0.21
Z-CF_3CH=CHCl	*HCFO-1233zd(Z)*	13 days	9.4×10^{-13}	1.64	0.67
cyc(-CH=$CFCF_2CF_2$-)	*1,3,3,4,4-Pentafluoro-cyclobutene*	270 days	6.2×10^{-14}	2.97	1.34

Chemical	Industrial Designation	Total Atmospheric Lifetime	OH radical rate-coefficient, (k_{OH}, cm^3 $molecule^{-1}s^{-1}$, 298 K, 1 atm)	$POCP_\varepsilon$, North-west European conditions (relative units)	$POCP_\varepsilon$, USA urban conditions (relative units)
cyc(-CH=CHCF_2CF_2-)	*3,3,4,4-Tetrafluoro-cyclobutene*	84 days	1.7×10^{-13} [d]	5.86	3.24

[a] Estimated total lifetime as quoted in Calvert et al. (Calvert et al., 2000).
[b] IPCC AR6 (Smith, 2021) erroneously gives an atmospheric lifetime of 122 days for this species. The atmospheric lifetime of 43 days quoted here is calculated as the tropospheric partial lifetime only due reaction with the OH radical. The value is a scaling to the lifetime for CH_3CCl_3 (6.1 years) using $k(OH+CH_3CCl_3, 272\ K) = 6.14 \times 10^{-15}$ cm^3 $molecule^{-1}$ s^{-1} (Burkholder et al., 2020), and the OH rate-coefficient listed in SI Table 3 for E-$(CF_3)_2$CFCH=CHF which is essentially temperature independent from 214-296 K (Papadimitriou & Burkholder, 2016).
[c] JPL Evaluation Number 19 (Burkholder et al., 2020) lists this as 1.35×10^{-13} but it should be 1.37×10^{-12} cm^3 $molecule^{-1}s^{-1}$ (Zhang et al., 2015).
[d] OH rate coefficient from Papadimitriou *et al.* (Papadimitriou et al., 2015).

SI 4 Estimated molar yields (%) of TFA from ODS replacements

Emissions and TFA yields in Figure 12 of the main text were estimated as outlined below.

SI 4.1.1 HCFC-123 (CF_3CHCl_2), 60 ± 10 %

CF_3CHCl_2 has a global annually averaged lifetime of 1.3 years (Smith, 2021). The main atmospheric fate of CF_3CHCl_2 is reaction with OH radicals in the troposphere. Atmospheric degradation leads to CF_3CCl_2O alkoxy radicals. Reaction of CF_3CCl_2O with O_2 is of minor importance and CF_3CCl_2O is converted to CF_3CClO through Cl elimination (Edney et al., 1991; Hayman et al., 1994; Tuazon & Atkinson, 1993). The estimated tropospheric photolytic lifetime for CF_3CClO for an overhead sun is 23 days (Calvert et al., 2008). The atmospheric lifetime of CF_3CClO with respect to uptake and hydrolysis in cloud water is 5-30 days (Wallington et al., 1994). On average about 60% of CF_3CClO is converted into TFA (Hayman et al., 1994) (see Figure 10 in the main text). The uncertainty in this yield is of the order of ± 5%. The yield of TFA in the atmospheric degradation of CF_3CHCl_2 is expected to be 60 ± 10 %.

CF_3CHCl_2 has been used as a refrigerant and a fire suppressant. Global annual emissions have been provided in a bottom-up estimate by Wuebbles & Patten (Wuebbles & Patten, 2009) as 0.130-0.135 Gg yr^{-1} for 2009.

SI 4.1.2 HCFC-124 (CF_3CHFCl), ~100%

CF_3CHFCl has a global annually averaged lifetime of 5.9 years (Smith, 2021). The main atmospheric fate of CF_3CHFCl is reaction with OH radicals in the troposphere. Atmospheric degradation of CF_3CHFCl leads to CF_3CFClO alkoxy radicals. The dominant, if not sole, fate of CF_3CFClO is decomposition to give CF_3CFO and Cl (Bhatnagar & Carr, 1995; Tuazon & Atkinson, 1993). The atmospheric fate of CF_3CFO is incorporation into water droplets followed by hydrolysis to give TFA, occurring on a time scale of 5-15 days (Wallington et al., 1994). The yield of TFA in the atmospheric degradation of CF_3CHFCl is expected to be very close to 100%.

CF_3CHFCl has been used as a refrigerant and fire suppressant. Global annual emissions have been estimated by Simmonds et al. (Simmonds et al., 2017) as 3.3±0.89 Gg yr−1 for 2015.

SI 4.1.3 HCFC-133a (CF_3CH_2Cl), 22 – 55 %

CF_3CH_2Cl has a global annually averaged lifetime of 4.6 years (Smith, 2021). The main atmospheric fate of CF_3CH_2Cl is reaction with OH radicals in the troposphere. The degradation initiated by OH radicals leads to the formation CF_3CHClO alkoxy radicals. The atmospheric fate of CF_3CHClO is decomposition and reaction with O_2 (Møgelberg, Nielsen, et al., 1995). Reaction with O_2 gives CF_3CClO and HO_2 while decomposition can proceed through three channels:

$CF_3CHClO + M \rightarrow CF_3 + HCOCl + M$ (1)

$CF_3CHClO + M \rightarrow CF_3CO + HCl + M$ (2)

$CF_3CHClO + M \rightarrow CF_3CHO + Cl + M$ (3)

At ground level and at room temperature, decomposition and reaction with O_2 are of equal importance, i.e., the yield of CF_3CClO is 52% (Møgelberg, Nielsen, et al., 1995). At higher altitudes, the importance of reaction with O_2 will increase. If CH_3CHClO is formed through reaction of CF_3CHClO_2 radicals with NO this can result in chemical activation of the resulting CF_3CHClO radicals, and in turn lead to enhanced decomposition.

The O_2 reaction channel product, CF_3CClO, undergoes photolysis in competition with incorporation into water droplets. The estimated tropospheric photolytic lifetime for CF_3CClO for an overhead sun is 23 days (Calvert et al., 2008). The atmospheric lifetime of CF_3CClO with respect to uptake and hydrolysis in cloud water is 5-30 days (Wallington et al., 1994). It has been estimated that, on average, 60% of CF_3CClO is converted into TFA (Hayman et al., 1994). Further reactions of CF_3CHClO decomposition products from reaction 2 and 3, CF_3CHO and CF_3CO, have the potential to yield TFA. Current understanding of the atmospheric fate of CF_3CHO suggests that its atmospheric fate is dominated by destruction by photolysis resulting in an atmospheric lifetime of the order of two days (Chiappero et al., 2006) (see also Sections 3.2 and 3.7.2 in the main text). Photolysis of CF_3CHO leads to CF_3 and CHO radicals which cannot contribute to the formation of TFA (Sulbaek Andersen & Nielsen, 2022). The rate of reaction of CF_3CHO with OH radicals is slow (atmospheric lifetime of approximately 20 days), and thus of less importance in the fate of CF_3CHO. Any oxidation of CF_3CHO initiated by OH radicals will produce CF_3CO radicals, which undergo reaction with O_2 to yield acyl peroxy radicals, $CF_3C(O)O_2$. These acyl peroxy radicals can react with HO_2, NO, or NO_2. Reaction of $CF_3C(O)O_2$ with HO_2 radicals can lead to the formation of TFA (39% yield) (Hurley et al., 2006). Finally, on contact with liquid water, CF_3CHO can produce aldehyde hydrates (gem-diols). These can, at least in the gas-phase, react with OH radicals (lifetime of approximately 90 days) and present an efficient way of generating TFA (Sulbaek Andersen et al., 2006). The latter two processes are likely minor fates for CF_3CHO. The importance of formation of TFA from the reaction of OH with CF_3CHO was indirectly accessed by Sulbaek Andersen et al. (Sulbaek Andersen, Schmidt, et al., 2018) in a global modeling study of HCFO-1233(zd). This model, which did not include potential CF_3CHO-hydrate formation, suggested a 2% yield of TFA from CF_3CHO. The contribution to TFA from CF_3CHO hydrate formation and processing remains highly uncertain (Franco et al., 2021; Sulbaek Andersen et al., 2006). Assuming that uptake into cloud water and hydration is efficient, effectively converting CF_3CHO into TFA on a timescale of 5 days (only limited by transport limitations, i.e., the lower limit for the time taken for transport into clouds (Wallington et al., 1994)), then a maximum TFA yield of 27% can be expected from the hydrate formation. Thus, the

TFA yield from processing of CF_3CHO is estimated at 2% with an upper theoretical limit of ~ 30%.

The acyl radical, CF_3CO, generated directly in reaction 2 can also lead to formation of TFA, though the reaction with O_2 and subsequent reaction of the acyl peroxy acyl radical with HO_2, as described above. Reaction with HO_2 radicals occurs in competition with reaction with NO which significantly reduces the maximum possible yield (39%) of TFA from the HO_2 reaction with $CF_3C(O)OO$ (Hurley et al., 2006). If the atmospheric lifetimes for CF_3CHO are 2 days and 20 days with respect to photolysis and reaction with OH radicals, respectively, then the model results of Sulbaek Andersen and co-workers (Sulbaek Andersen, Schmidt, et al., 2018) suggest, that the molar yield of TFA, starting with CF_3CO, can be on the order of ~20 ± 10 %.

Based on the discussion above regarding the fate of CF_3CClO, the yield of TFA from hydrolysis of CF_3CClO can be calculated as 0.52 × (60%) = 31%. An uncertainty of ± 10% is estimated for this yield. Additional contributions from the atmospheric processing of CF_3CHO or CF_3CO could be as much as 0.48 × (2-30%) = 1-14%. Hence, the best estimate of the yield of TFA in the atmospheric degradation of CF_3CH_2Cl is 32%, but with an upper limit of 55% and a lower limit of 22%. Without a detailed atmospheric chemistry and transport model study, a more quantitative assessment of the yield of TFA is not possible.

CF_3CH_2Cl has been used as a chemical feedstock/ intermediate. Global annual emissions have been estimated by Vollmer et al. (Vollmer, Rigby, et al., 2015) as ~1.5 Gg yr^{-1} for 2014.

SI 4.2.1 HFC-125 (CF_3CF_2H), ~1–10%

CF_3CF_2H has an atmospheric lifetime of approximately 30 years (Smith, 2021). The atmospheric fate of CF_3CF_2H is reaction with OH radicals. Reaction with OH produces an alkyl radical that reacts with oxygen to give the peroxy radical $CF_3CF_2O_2$. The peroxy radical reacts with NO to yield the alkoxy radical CF_3CF_2O which decomposes to give COF_2 and CF_3 radicals. However, Ellis et al. (Ellis et al., 2004) suggested a mechanism by which $CF_3CF_2O_2$ radicals can react with α-hydrogen containing peroxy radicals (e.g., CH_3O_2) in the atmosphere to give CF_3CF_2OH in small amounts (1-10%) (Wallington et al., 2006)). CF_3CF_2OH will eliminate HF to give CF_3CFO. The sole atmospheric fate of CF_3CFO is incorporation into water droplets followed by hydrolysis, occurring on a time scale of 5-15 days to give TFA (Wallington et al., 1994). Thus, the yield of TFA from CF_3CF_2H is estimated as 1–10%.

CF_3CF_2H is used as a fire suppressant. Global annual emissions have been estimated as 59.7±9.5 Gg yr^{-1} in 2015 (Simmonds et al., 2017).

SI 4.2.2 HFC-134a (CF_3CH_2F), 7–20 %

CF_3CH_2F has an atmospheric lifetime of 14 years (Smith, 2021). Reaction of OH radicals with CF_3CH_2F yields CF_3CHFO_2 radicals, which react with either other peroxy radicals, RO_2, or NO. Reaction with RO_2 yields a stabilized CF_3CHFO radical, which can either decompose or react with O_2 to give CF_3CFO. The atmospheric fate of CF_3CFO is incorporation into water droplets occurring on a time scale of 5–15 days (Wallington et al., 1994) and subsequent hydrolysis yields TFA. Reaction of CF_3CFO_2 with NO can produce excited peroxy radicals, CF_3CFHO, which undergo rapid decomposition and limit the formation of CF_3CFO to a range of 7 to 20 %, depending on conditions (see Figure 10 in the main text) (Wallington et al., 1996). Thus, the TFA yield is estimated as 7–20%.

CF_3CH_2F is used as a refrigerant. Global annual emissions in 2015 were estimated at 209.0±42.9 Gg yr^{-1} (Simmonds et al., 2017) and estimated in the Science Assessment Panel 2022 report (WMO (2022) as 64-192 Gg yr^{-1} for 2020. The latter is currently the best estimate for this refrigerant (see also Table 3 in the main text).

SI 4.2.3 HFC-143a (CF_3CH_3), 2–30%

CH_3CF_3 has a global annually averaged lifetime of 51 years (Smith, 2021). The atmospheric fate of CH_3CF_3 is reaction with OH radicals. Reaction with OH produces an alkyl radical that reacts with O_2 and NO to yield the alkoxy radical, CF_3CH_2O. The dominant fate of CF_3CH_2O in the atmosphere is reaction with O_2 to give CF_3CHO and HO_2 (Nielsen et al., 1994). Current understanding of the atmospheric fate of CF_3CHO suggests that its atmospheric lifetime is dominated by photolysis. As discussed in SI Section 4.1.3, atmospheric processing of CF_3CHO can lead to formation of TFA in small amounts. Thus, the TFA yield from processing of CH_3CF_3 is estimated at 2%, with an upper limit of ~ 30%.

CH_3CF_3 is used as a refrigerant. Global annual emissions in 2015 have been estimated as 27.4±3.0 Gg yr^{-1} (Simmonds et al., 2017).

SI 4.2.4 HFC-227ea (CF_3CHFCF_3), ~100%

CF_3CHFCF_3 has an atmospheric lifetime of 36 years (Smith, 2021). The atmospheric fate of CF_3CHFCF_3 is reaction with OH radicals. Reaction with OH produces an alkyl radical that reacts with O_2 and NO to yield the alkoxy radical CF_3CFOCF_3. The atmospheric fate of CF_3CFOCF_3 is decomposition via C-C cleavage to give CF_3CFO and CF_3 radicals (Zellner et al., 1994). The atmospheric fate of CF_3CFO is incorporation into water droplets followed by hydrolysis to give TFA, occurring on a time scale of 5-15 days (Wallington et al., 1994). The yield of TFA in the atmospheric degradation of CF_3CHFCF_3 is 100%.

CF_3CHFCF_3 is used as a fire extinguishant. Global annual emissions have been estimated as 2.53±0.99 Gg yr^{-1} for 2010 (Vollmer et al., 2011).

SI 4.2.5 HFC-236fa ($CF_3CH_2CF_3$), 20 ± 10 %

$CF_3CH_2CF_3$ has an atmospheric lifetime of 213 years (Smith, 2021). Reaction of OH radicals with $CF_3CH_2CF_3$ generate CF_3CHCF_3 radicals which react with oxygen and NO to yield the alkoxy radicals, CF_3CHOCF_3. The sole fate of CF_3CHOCF_3 is reaction with O_2 to yield CF_3COCF_3 (Møgelberg, Platz, et al., 1995). The dominant fate of CF_3COCF_3 is likely tropospheric photolysis to give CF_3 and CF_3CO radicals. As discussed in SI Section 4.1.3, further reaction of CF_3CO radicals with O_2 and HO_2 radicals can lead to formation of TFA in small amounts. Thus, the yield of TFA in the atmospheric oxidation of $CF_3CH_2CF_3$ is estimated at 20% (± 10%).

$CF_3CH_2CF_3$ is used as a fire suppressant. Global annual emissions have been estimated as 0.16±0.11 Gg yr^{-1} for 2010 (Vollmer et al., 2011).

SI 4.2.6 HFC-245fa ($CHF_2CH_2CF_3$) 1 – 17%

$CHF_2CH_2CF_3$ has an atmospheric lifetime of 7.9 years (Smith, 2021). The atmospheric fate of $CHF_2CH_2CF_3$ is reaction with OH radicals. Structure activity relationships (SAR) suggests that approximately 56% of the reaction of OH radicals with $CHF_2CH_2CF_3$ proceeds through hydrogen

abstraction from the terminal -CHF_2 group (Calvert et al., 2008). This will lead to the formation of COF_2 and CF_3CHO as major products (Chen et al., 1997). Hydrogen abstraction from the -CH_2-_group is expected to produce a ketone, $CHF_2C(O)CF_3$, through reaction of CHF_2CHOCF_3 with O_2. By analogy to CF_3COCH_3 (Wallington et al., 1994), the main sink for $CHF_2C(O)CF_3$ is expected to be photolysis, yielding COF_2 and CO_2. Thus, the expected yield of TFA in the atmospheric oxidation of $CF_3CH_2CF_2CH_3$ is 0.56 × (2 – 30%) = 1%, with 17% as an upper limit.

$CHF_2CH_2CF_3$ is used as a blowing agent. Global annual emissions have been estimated as 6.77±0.79 Gg yr^{-1} for 2010 (Vollmer et al., 2011).

SI 4.2.7 HFC-365mfc ($CF_3CH_2CF_2CH_3$), 2–30%

$CF_3CH_2CF_2CH_3$ has an atmospheric lifetime of 8.9 years (Smith, 2021). The atmospheric fate of $CF_3CH_2CF_2CH_3$ is reaction with OH radicals. The reaction of OH radicals with $CF_3CH_2CF_2CH_3$ proceeds mainly via attack on the –CH_3 group leading to formation of $CF_3CH_2CF_2CHO$, which in turn is oxidized to CF_3CHO and COF_2 (Inoue et al., 2008). Oxidation of $CF_3CH_2CF_2CHO$ will generate CF_3CHO and COF_2 as secondary products. The only study in the literature on the atmospheric oxidation mechanism of $CF_3CH_2CF_2CHO$ used Cl atoms as a surrogate for OH radicals. However, based on SAR, approximately 76% of the reaction of OH radicals, proceeds through abstraction at the -CH_3 site (Calvert et al., 2008). Reaction at the -CH_2- group would produce a ketone, $CF_3C(O)CF_2CH_3$. Further oxidation of $CF_3C(O)CF_2CH_3$ initiated by OH radicals will generate CF_3CO radicals (and COF_2, CO and CO_2). As discussed in SI Section 4.1.3 both CF_3CHO and CF_3CO radicals can lead to formation of TFA in small amounts. Thus, the expected yield of TFA in the atmospheric oxidation of $CF_3CH_2CF_2CH_3$ is 2%, with 30% as the upper limit. $CF_3CH_2CF_2CH_3$ is used as a blowing agent and refrigerant.

Global annual emissions have been estimated as 2.87±0.60 Gg yr^{-1} for 2010 (Vollmer et al., 2011).

SI 4.2.8 HFC-43-10mee ($CF_3CF_2CFHCFHCF_3$), 54 – 60%

$CF_3CF_2CFHCFHCF_3$ has an atmospheric lifetime of 17 years (Smith, 2021). The atmospheric fate of $CF_3CF_2CFHCFHCF$ is reaction with OH radicals. There has been no mechanistic study conducted on the atmospheric degradation of $CF_3CF_2CFHCFHCF_3$. SAR suggests that approximately half of the reaction proceeds through hydrogen abstraction from the -CHF- group, alpha to the terminal CF_3 group. (Calvert et al., 2008). Reaction of OH radicals with $CF_3CF_2CFHCFHCF_3$ followed by reactions with O_2 and NO will therefore yield both $CF_3CF_2CFOCFHCF_3$ and $CF_3CF_2CFHCFOCF_3$ radicals. These will likely undergo decomposition to the give acyl fluorides, CF_3CF_2CFO and CF_3CFO, and radicals $CFHCF_3$ and CF_3CF_2CFH. The latter two will react with O_2 and RO_2/NO yielding CF_3CHFO and CF_3CF_2CHFO radicals. Reaction of CF_3CHFO and CF_3CF_2CHFO (Møgelberg et al., 1997) with NO can produce excited peroxy radicals which undergo rapid decomposition (see discussion in SI Section **4.2.2** and Figure 10 in the main text). The decomposition pathways will give CFHO, CF_3 and CF_3CF_2 radicals. Reaction with O_2 will lead to CF_3CFO, and CF_3CF_2CFO. The atmospheric fate of CF_3CFO, and likely also CF_3CF_2CFO, is incorporation into water droplets followed by hydrolysis to give TFA and CF_3CF_2COOH, occurring on a time scale of 5-15 days (Wallington et al., 1994). Thus, the yield of TFA in the atmospheric oxidation of $CF_3CF_2CFHCFHCF_3$ is estimated as 50% + ~0.5 × (7 – 20%) = 54 – 60%. A similar yield of CF_3CF_2COOH is also expected.

$CF_3CF_2CFHCFHCF_3$ is used a solvent. Annual global emissions of $CF_3CF_2CFHCFHCF$ have been estimated as 1.13±0.31 Gg yr^{-1} for 2012 (Arnold et al., 2014).

SI 4.3.1 HFO-1234yf ($CF_3CF=CH_2$), ~100%

$CF_3CF=CH_2$ has an atmospheric lifetime of 12 days (Smith, 2021). Atmospheric oxidation of $CF_3CF=CH_2$ proceeds through OH addition to the double bond (see Fig. 10 in the main text). CF_3CFO is subsequently formed in a yield of 100%, independent of which side of the double bond is involved in the initial OH-addition step (Hurley et al., 2008). The atmospheric fate of CF_3CFO is incorporation into water droplets occurring on a time scale of 5–15 days (Wallington et al., 1994), and subsequent hydrolysis yields TFA. The yield of TFA in the atmospheric oxidation of $CF_3CF=CH_2$ is estimated at 100%.

$CF_3CF=CH_2$ is used a refrigerant. Annual global emissions of $CF_3CF=CH_2$ have been estimated in the Science Assessment Panel 2022 report (WMO 2022) as 30 Gg yr^{-1} for 2020 (see also Table 3 in the main text).

SI 4.3.2 HFO-1234ze(E) ($CF_3CH=CHF$), 2–30%

$CF_3CF=CH_2$ has an atmospheric lifetime of 19 days (Smith, 2021). Atmospheric oxidation of $CF_3CH=CHF$ proceeds through OH addition to the double bond (Javadi et al., 2008). Subsequent reaction with O_2 and NO/RO_2 leads to the formation of CF_3CHO and HC(O)F in yields indistinguishable from 100%. As discussed in SI Section 4.1.3, atmospheric processing of CF_3CHO can lead to formation of TFA in small amounts. Thus, the TFA yield from processing of $CF_3CF=CH_2$ is estimated at 2%, with an upper limit of ~ 30%.

$CF_3CH=CHF$ is used as an aerosol propellant, in expanded polystyrene (styrofoam) insulation industry, and as a refrigerant. Annual global emissions of $CF_3CH=CHF$ are not known.

SI 4.3.3 HFO-1336mzz(E) (E-$CF_3CH=CHCF_3$) and HFO-1336mzz(Z) (Z-$CF_3CH=CHCF_3$), 4–60%

E- and Z- $CF_3CH=CHCF_3$ have atmospheric lifetimes of 122 and 27 days, respectively (Smith, 2021). Atmospheric oxidation of E- and Z- $CF_3CH=CHCF_3$ proceeds through OH addition to the double bond. Østerstrøm et al. (Østerstrøm et al., 2017) used Cl atoms in their study of the atmospheric oxidation of E- and Z- $CF_3CH=CHCF_3$. The OH initiated mechanism has not been studied in detail and remains speculative. The initially formed hydroxy-substituted alkoxy radicals will have competing fates of decomposition and reaction with O_2. It is possible that CF_3CHO is formed in this initial step through decomposition, or later through further reactions of possible degradation products, such as $CF_3CH(OH)C(O)CF_3$. An upper limit for the yield of CF_3CHO is 200%. As discussed in SI Section 4.1.3, atmospheric processing of CF_3CHO can lead to formation of TFA in small amounts. Thus, the TFA yield from processing of CF_3CHO is estimated at 4%, with an upper limit of ~ 60%.

E- and Z- $CF_3CH=CHCF_3$ are used as refrigerants. Annual global emissions are not known.

SI 4.3.4 HCFO-1233zd(E) (E-CF_3CH=CHCl) and HCFO-1233zd(Z) (Z-CF_3CH=CHCl), 2–30%

E- and Z- CF_3CH=CHCl have atmospheric lifetimes of 42 and 13 days, respectively (Smith, 2021). Atmospheric oxidation of E- and Z- CF_3CH=CHCl proceeds through OH addition to the double bond (M. P. Sulbaek Andersen et al., 2012; Sulbaek Andersen, Sølling, et al., 2018). The atmospheric degradation pathway for CF_3CH=CHCl is complex and produces CF_3CHO with estimated yield of 100% (see Section 3.2 and Figure 11 in the main text). As discussed in detail in SI Section 4.1.3, atmospheric processing of CF_3CHO can lead to formation of TFA in small amounts. Thus, the TFA yield from processing of CF_3CH=CHCl is estimated at 2%, with an upper limit of ~ 30%.

E- CF_3CH=CHCl is used as a polyurethane foam blowing agent and as a refrigerant. Z- CF_3CH=CHCl is used for degreasing of mechanical parts and dry cleaning. Annual global emissions for E- CF_3CH=CHCl have been estimated by Vollmer et al. (Vollmer, Reimann, et al., 2015) as 0.5 Gg yr^{-1} for 2014.

SI 4.3.5 2-BTP (CF_3CBr=CH_2), 2–30%

CF_3CBr=CH_2 has an estimated atmospheric lifetime of approximately 3 days (Sulbaek Andersen et al., 2009). Atmospheric oxidation of CF_3CBr=CH_2 proceeds through OH addition to the double bond. There is no mechanistic study available in the literature of the atmospheric oxidation of CF_3CBr=CH_2. Sulbaek Andersen et al. (Sulbaek Andersen et al., 2009) speculate that the OH relation leads to the formation of an enol $CF_3C(OH)$=CH_2 or a carbonyl/alcohol compound, $CF_3C(O)$-CH_2OH. These oxidation products would be reactive towards OH radicals and, in the case of $CF_3C(OH)$=CH_2, possibly undergo keto-enol tautomerization. It is possible that CF_3CHO is formed though reaction of OH radicals with the oxidation products or though photolysis of the carbonyl products. An upper limit for the yield of CF_3CHO is 100%. As discussed in SI Section 4.1.3, atmospheric processing of CF_3CHO can lead to formation of TFA in small amounts. Thus, the TFA yield from atmospheric processing of CF_3CBr=CH_2 is estimated at 2%, with an upper limit of ~ 30%.

CF_3CBr=CH_2 is used as a fire extinguishant. Global annual emissions are not known.

SI 4.4.1 Halothane ($CF_3CHBrCl$), 60 ± 10%

$CF_3CHBrCl$ has an atmospheric lifetime of 1 year (Smith, 2021). The reaction of OH radicals with $CF_3CHBrCl$ proceeds via hydrogen abstraction followed by reaction with O_2 and NO, and subsequent Br elimination, to give CF_3CClO in a yield indistinguishable from 100% (Bilde et al., 1998). The estimated tropospheric photolytic lifetime for CF_3CClO for an overhead sun is 23 days (Calvert et al., 2008). The atmospheric lifetime of CF_3CClO with respect to uptake and hydrolysis in cloud water is 5-30 days (Wallington et al., 1994). It has been estimated that, on average, 60% of CF_3CClO is converted into TFA (Hayman et al., 1994). The uncertainty on this yield is likely on the order of ± 10%. Thus, the TFA yield from atmospheric processing of $CF_3CHBrCl$ is estimated at 60 ±10%.

Halothane is used as an inhaled anesthetic agent. Global annual emissions have been estimated by Vollmer et al. (Vollmer, Rhee, et al., 2015) as 0.25 Gg yr^{-1} for 2014.

SI 4.4.2 Isoflurane ($CF_3CHClOCHF_2$), 95 ± 3%

$CF_3CHClOCHF_2$ has an atmospheric lifetime of approximately 3 years (Mads P. Sulbaek Andersen et al., 2012). The atmospheric oxidation of $CF_3CHClOCHF_2$ proceeds via OH-mediated hydrogen abstraction. An estimated 95% of the reaction occurs at the -CHCl- group. Cl elimination subsequently yields the main oxidation product, $CF_3C(O)OCHF_2$ (95 ± 3%) (Wallington et al., 2002). The atmospheric lifetime of $CF_3C(O)OCHF_2$ with respect to reaction with OH is unknown. The atmospheric lifetime for a similar ester, $CF_3C(O)OCH_3$ is approximately 7.5 months (Blanco et al., 2010; Wallington et al., 1988). The atmospheric lifetime is likely to be longer for $CF_3C(O)OCHF_2$. Reaction of OH with $CF_3C(O)OCHF_2$ is not expected to lead to the formation of TFA. Loss of $CF_3C(O)OCHF_2$ via uptake into sea water followed by hydrolysis to give TFA is therefore estimated to be a major atmospheric sink for $CF_3C(O)OCHF_2$ (Kutsuna et al., 2004). The expected yield of TFA in the atmospheric oxidation of $CF_3CHClOCHF_2$ is 95 ± 3%.

Isoflurane is used as an inhaled anesthetic agent. Global annual emissions have been estimated by Vollmer et al. (Vollmer, Rhee, et al., 2015) as 0.88 Gg yr^{-1} for 2014.

SI 4.4.3 Desflurane ($CF_3CHFOCHF_2$), 3–20%

$CF_3CHFOCHF_2$ has an atmospheric lifetime of 9 years (Smith, 2021). The atmospheric fate of $CF_3CHFOCHF_2$ is reaction with OH radicals. No study of the OH initiated oxidation mechanism for $CF_3CHFOCHF_2$ exists in the literature. Based on a study of the chlorine atom initiated oxidation, Sulbaek Andersen et al. (Mads P. Sulbaek Andersen et al., 2012) proposed that the hydrogen abstraction reaction proceeds predominantly from the -CHF- carbon group (83%). Only hydrogen abstraction from the terminal carbon will have the potential to lead to TFA (Mads P. Sulbaek Andersen et al., 2012). Hydrogen abstraction from the terminal carbon (17%) will generate $CF_3CHFOCF_2$ radicals, which will react with O_2 and NO to give $CF_3CHFOCF_2O$ radicals. These will undergo decomposition to give COF_2 and CF_3CHFO radicals. The latter will in one atmosphere of air react with O_2 to give CF_3CFO (18%) (Mads P. Sulbaek Andersen et al., 2012). The atmospheric fate of CF_3CFO is incorporation into water droplets followed by hydrolysis to give TFA, occurring on a time scale of 5-15 days (Wallington et al., 1994). The TFA yield from atmospheric processing of $CF_3CHClOCHF_2$ can be estimated as $0.17 \times 0.18 \times (100\%) = 3\ \%$. A 20% upper limit of the estimate to account for possible differences in the location of hydrogen abstraction for the OH mediated abstraction, i.e., 3 – ~20%.

Desflurane is used as an inhaled anesthetic agent. Global annual emissions have been estimated as 0.96 Gg yr^{-1} for 2014 (Vollmer, Rhee, et al., 2015).

SI 4.4.4 Sevoflurane ($(CF_3)_2HCOCH_2F$), 2–95%

$(CF_3)_2HCOCH_2F$ has an atmospheric lifetime of 1.4 years (Sulbaek Andersen et al., 2021). The atmospheric fate of $(CF_3)_2HCOCH_2F$ is reaction with OH radicals. No study of the OH initiated oxidation mechanism for $CF_3CHFOCHF_2$ exists in the literature. Based on a study of the chlorine atom initiated oxidation, Sulbaek Andersen et al. (Mads P. Sulbaek Andersen et al., 2012) proposed that the hydrogen abstraction reaction proceeds exclusively from the terminal $-CH_2F$ group. Reaction of the initially formed alkoxy radical with O_2 and NO yields $(CF_3)_2HCOCHFO$, which in one atmosphere of air was found to give 7% CF_3COCF_3 (through decomposition) and 93% $(CF_3)_2HCOCFO$ (through reaction with O_2). The dominant fate of CF_3COCF_3 is likely tropospheric photolysis to give CF_3 and CF_3CO radicals (Calvert et al., 2008). As discussed in SI

Section 4.1.3, further reaction of CF_3CO radicals with O_2 and HO_2 radicals can lead to formation of TFA in small amounts (20 ± 10%). The major fate of $(CF_3)_2HCOCFO$ will be dissolution into seawater followed by hydrolysis (Kutsuna et al., 2004). Hydrolysis would possibly result in the formation of $(CF_3)_2HCOCOOH_{(aq)}$, but it is unclear if the hydrolysis of $(CF_3)_2HCOCFO$ would also lead to TFA. A possible upper limit for the estimated yield of TFA from ocean uptake and hydrolysis of $(CF_3)_2HCOCFO$ is 93%. Thus, the yield of TFA from atmospheric processing of $(CF_3)_2HCOCH_2F$ can be estimated as 2% (photolysis of CF_3COCF_3), with an upper limit of 95% (ocean uptake and hydrolysis of $(CF_3)_2HCOCFO$).

Sevoflurane is used as an inhaled anesthetic agent. Global annual emissions have been estimated as 1.20 Gg yr^{-1} for 2014 (Vollmer, Rhee, et al., 2015).

SI 4.4.5 FK-5-1-12 ($CF_3CF_2C(O)CF(CF_3)_2$), 101-110%

$CF_3CF_2C(O)CF(CF_3)_2$ has an atmospheric lifetime of 7 days (Smith, 2021). The atmospheric oxidation of $CF_3CF_2C(O)CF(CF_3)_2$ has been studied by Taniguchi et al. (Taniguchi et al., 2003). The main atmospheric fate of $CF_3CF_2C(O)CF(CF_3)_2$ is removal by photolysis. Photolysis yields CF_3CF_2 + $C(O)CF(CF_3)_2$ radicals and subsequent reactions of the $C(O)CF(CF_3)_2$ yields CF_3CFO in a molar yield of unity. The atmospheric fate of CF_3CFO is incorporation into water droplets followed by hydrolysis to give TFA, occurring on a time scale of 5-15 days (Wallington et al., 1994). CF_3CF_2 will react with O_2 and NO to give CF_3CF_2O radicals. Ellis et al. (Ellis et al., 2004) suggested a mechanism by which the reaction of molecules such as CF_3CF_2O react with α-hydrogen containing peroxy radicals (e.g., CH_3O_2) in the atmosphere to give CF_3CF_2OH in small amounts (1-10%) (Wallington et al., 2006)). CF_3CF_2OH will eliminate HF to give CF_3CFO. Thus, the yield of TFA in the atmospheric degradation of $CF_3CF_2C(O)CF(CF_3)_2$ is expected to be essentially 101%, with an upper limit of 110%.

$CF_3CF_2(O)CF(CF_3)_2$ is used as a fire suppressant. Global annual emissions are unknown.

References

Arnold, T., Ivy, D. J., Harth, C. M., Vollmer, M. K., Mühle, J., Salameh, P. K., Paul Steele, L., Krummel, P. B., Wang, R. H. J., Young, D., Lunder, C. R., Hermansen, O., Rhee, T. S., Kim, J., Reimann, S., O'Doherty, S., Fraser, P. J., Simmonds, P. G., Prinn, R. G., & Weiss, R. F. (2014). HFC-43-10mee atmospheric abundances and global emission estimates. *Geophysical Research Letters*, *41*(6), 2228-2235. https://doi.org/https://doi.org/10.1002/2013GL059143

ATSDR. (2021). *Toxicological Profile for Perfluoroalkyls*. ATSDR. https://www.atsdr.cdc.gov/toxprofiles/tp200.pdf

Bhatnagar, A., & Carr, R. W. (1995). Temperature Dependence of the Reaction of CF_3CFClO_2 Radicals with NO and the Unimolecular Decomposition of the CF_3CFClO Radical. *The Journal of Physical Chemistry*, *99*(49), 17573-17577. https://doi.org/10.1021/j100049a017

Bilde, M., Wallington, T. J., Ferronato, C., Orlando, J. J., Tyndall, G. S., Estupiñan, E., & Haberkorn, S. (1998). Atmospheric Chemistry of CH_2BrCl, $CHBrCl_2$, $CHBr_2Cl$, $CF_3CHBrCl$, and CBr_2Cl_2. *The Journal of Physical Chemistry A*, *102*(11), 1976-1986. https://doi.org/10.1021/jp9733375

Blanco, M. B., Bejan, I., Barnes, I., Wiesen, P., & Teruel, M. a. (2010). Atmospheric Photooxidation of Fluoroacetates as a Source of Fluorocarboxylic Acids. *Environmental Science & Technology*, *44*(7), 2354-2359. https://doi.org/10.1021/es903357j

Boudreau, T. M. (2002). *Toxicity of Perfluorinated Organic Acids to Selected Freshwater Organisms under Laboratory and Field Conditions* [M.Sc., University of Guelph]. Guelph.

Boutonnet, J. C., Bingham, P., Calamari, D., Rooij, C. d., Franklin, J., Kawano, T., Libre, J.-M., McCul-Loch, A., Malinverno, G., & Odom, J. M. (1999). Environmental risk assessment of trifluoroacetic acid. *Human and Ecological Risk Assessment*, *5*, 59-124. https://doi.org/10.1080/10807039991289644

Burkholder, J. B., Sander, S. P., Abbatt, J., Barker, J. R., Cappa, C., Crounse, J. D., Dibble, T. S., Huie, R. E., Kolb, C. E., Kurylo, M. J., Orkin, V. L., Perciva, C. J., Wilmouth, D. M., & Wine, P. H. (2020). *Chemical Kinetics and Photochemical Data for Use in Atmospheric Studies*. http://jpldataeval.jpl.nasa.gov

Calvert, J. G., Atkinson, R., Kerr, J. A., & Madronich, S., Moortgat, G., Wallington, T. J., & Yarwood, G. (2000). *The Mechanisms of Atmospheric Oxidation of the Alkenes*. Oxford University Press.

Calvert, J. G., Derwent, R. G., Orlando, J. J., Tyndall, G. S., & Wallington, T. J. (2008). *Mechanisms of Atmospheric Oxidation of the Alkanes*. Oxford University Press.

Chabot, L. (2017). *Chabot L. (2017). ALGA, GROWTH INHIBITION TEST Effect of the trifuoroacetic acid on the growth of the unicellular alga Pseudokirchneriella subcapitata, according to OECD guideline 201 (Study conducted for RHODIA OPERATIONS – SOLVAY)*.

Chen, J., Young, V., Niki, H., & Magid, H. (1997). Kinetic and Mechanistic Studies for Reactions of $CF_3CH_2CHF_2$ (HFC-245fa) Initiated by H-Atom Abstraction Using Atomic Chlorine. *The Journal of Physical Chemistry A*, *101*(14), 2648-2653. https://doi.org/10.1021/jp963735s

Chiappero, M. S., Malanca, F. E., Argüello, G. A., Wooldridge, S. T., Hurley, M. D., Ball, J. C., Wallington, T. J., Waterland, R. L., & Buck, R. C. (2006). Atmospheric chemistry of perfluoroaldehydes (C_xF_{2x}+1CHO) and fluorotelomer aldehydes (C_xF_{2x}+1CH_2CHO): Quantification of the important role of photolysis. *The Journal of Physical Chemistry A*, *110*(43), 11944-11953. https://doi.org/10.1021/jp064262k

Cox, R. A., Ammann, M., Crowley, J. N., Herrmann, H., Jenkin, M. E., McNeill, V. F., Mellouki, A., Troe, J., & Wallington, T. J. (2020). Evaluated kinetic and photochemical data for atmospheric chemistry: Volume VII – Criegee intermediates. *Atmospheric Chemistry and Physics*, *20*(21), 13497-13519. https://doi.org/10.5194/acp-20-13497-2020

Edney, E. O., Gay, B. W., & Driscoll, D. J. (1991). Chlorine initiated oxidation studies of hydrochlorofluorocarbons: Results for HCFC-123 (CF_3CHCl_2) and HCFC-141b ($CFCl_2CH_3$). *Journal of Atmospheric Chemistry*, *12*(2), 105-120. https://doi.org/10.1007/BF00115774

EFSA. (2020). Risk to human health related to the presence of perfluoroalkyl substances in food. *EFSA Journal*, *18*(9), e06223. https://doi.org/https://doi.org/10.2903/j.efsa.2020.6223

Ellis, D. A., Martin, J. W., De Silva, A. O., Mabury, S. A., Hurley, M. D., Sulbaek Andersen, M. P., & Wallington, T. J. (2004). Degradation of fluorotelomer alcohols: A likely atmospheric source of perfluorinated carboxylic acids. *Environmental Science & Technology*, *38*, 3316-3321. https://doi.org/10.1021/es049860w

Franco, B., Blumenstock, T., Cho, C., Clarisse, L., Clerbaux, C., Coheur, P. F., De Mazière, M., De Smedt, I., Dorn, H. P., Emmerichs, T., Fuchs, H., Gkatzelis, G., Griffith, D. W. T., Gromov, S., Hannigan, J. W., Hase, F., Hohaus, T., Jones, N., Kerkweg, A., . . . Taraborrelli, D. (2021). Ubiquitous atmospheric production of organic acids mediated by cloud droplets. *Nature*, *593*(7858), 233-237. https://doi.org/10.1038/s41586-021-03462-x

Hayman, G. D., Jenkin, M. E., Murrells, T. P., & Johnson, C. E. (1994). Trospospheric degradation chemistry of HCFC-123 (CF_3CHCl_2): A proposed replacement chlorofluorocarbon. *Atmospheric Environment*, *28*(3), 421-437. https://doi.org/https://doi.org/10.1016/1352-2310(94)90121-X

Hurley, M. D., Ball, J. C., Wallington, T. J., Sulbaek Andersen, M. P., Nielsen, O. J., Ellis, D. A., Martin, J. W., & Mabury, S. A. (2006). Atmospheric Chemistry of n-C^xF2_{x+1}CHO (x = 1, 2, 3, 4): Fate of n-C_xF_{2x+1}C(O) Radicals. *The Journal of Physical Chemistry A*, *110*(45), 12443-12447. https://doi.org/10.1021/jp064029m

Hurley, M. D., Wallington, T. J., Javadi, M. S., & Nielsen, O. J. (2008). Atmospheric chemistry of CF3CFCH2: Products and mechanisms of Cl atom and OH radical initiated oxidation. *Chemical Physics Letters*, *450*(4-6), 263-267. https://doi.org/10.1016/j.cplett.2007.11.051

Inoue, Y., Kawasaki, M., Wallington, T. J., & Hurley, M. D. (2008). Atmospheric chemistry of $CF_3CH_2CF_2CH_3$ (HFC-365mfc): Kinetics and mechanism of chlorine atom initiated oxidation, infrared spectrum, and global warming potential. *Chemical Physics Letters*, *462*(4), 164-168. https://doi.org/https://doi.org/10.1016/j.cplett.2008.07.054

Javadi, M. S., Søndergaard, R., Nielsen, O. J., Hurley, M. D., & Wallington, T. J. (2008). Atmospheric chemistry of *trans*-CF_3CH=CHF: products and mechanisms of hydroxyl radical and chlorine atom initiated oxidation. *Atmospheric Chemistry and Physics*, *8*(12), 3141-3147. https://doi.org/10.5194/acp-8-3141-2008

Jenkin, M. E. (2022). Personal Communication.

Jenkin, M. E., Derwent, R.G., Wallington, T.J. (2017). Photochemical ozone creation potentials for volatile organic compounds: Rationalization and estimation. *Atmospheric Environment 163*, 128–137. https://doi.org/10.1016/j.atmosenv.2017.05.024

Kutsuna, S., Chen, L., Ohno, K., Tokuhashi, K., & Sekiya, A. (2004). Henry's law constants and hydrolysis rate constants of 2,2,2-trifluoroethyl acetate and methyl trifluoroacetate. *Atmospheric Environment*, *38*(5), 725-732. https://doi.org/https://doi.org/10.1016/j.atmosenv.2003.10.019

Møgelberg, T. E., Nielsen, O. J., Sehested, J., & Wallington, T. J. (1995). Atmospheric chemistry of HCFC-133a: the UV absorption spectra of CF_3CClH and CF_3CClHO_2 radicals, reactions of CF_3CClHO_2 with NO and NO_2, and fate of CF_3CClHO radicals. *The Journal of Physical Chemistry*, *99*(36), 13437-13444. https://doi.org/10.1021/j100036a018

Møgelberg, T. E., Platz, J., Nielsen, O. J., Sehested, J., & Wallington, T. J. (1995). Atmospheric Chemistry of HFC-236fa: Spectrokinetic Investigation of the $CF_3CHO_2CF_3$ Radical, Its Reaction with NO, and the Fate of the CF_3CHOCF_3 Radical. *The Journal of Physical Chemistry*, *99*(15), 5373-5378. https://doi.org/10.1021/j100015a021

Møgelberg, T. E., Sehested, J., Tyndall, G. S., Orlando, J. J., Fracheboud, J.-M., & Wallington, T. J. (1997). Atmospheric Chemistry of HFC-236cb: Fate of the Alkoxy Radical CF_3CF_2CFHO. *The Journal of Physical Chemistry A*, *101*(15), 2828-2832. https://doi.org/10.1021/jp963021h

NICNAS. (2016). *Short Chain Perfluorocarboxylic Acids and their Direct Precursors: Human Health Tier II Assessment*. N. I. C. N. a. A. S. (NICNAS). https://www.nicnas.gov.au/chemical-information/imap-assessments/imap-group-assessment-report?assessment_id=1686

Nielsen, O. J., Gamborg, E., Sehested, J., Wallington, T. J., & Hurley, M. D. (1994). Atmospheric Chemistry of HFC-143a: Spectrokinetic Investigation of the $CF_3CH_2O_2$ Radical, Its Reactions with NO and NO_2, and the Fate of CF_3CH_2O. *The Journal of Physical Chemistry*, *98*(38), 9518-9525. https://doi.org/10.1021/j100089a026

Østerstrøm, F. F., Andersen, S. T., Sølling, T. I., Nielsen, O. J., & Sulbaek Andersen, M. P. (2017). Atmospheric chemistry of Z- and E-CF_3CH=$CHCF_3$ [10.1039/C6CP07234H]. *Physical Chemistry Chemical Physics*, *19*(1), 735-750. https://doi.org/10.1039/C6CP07234H

Papadimitriou, V. C., & Burkholder, J. B. (2016). OH Radical Reaction Rate Coefficients, Infrared Spectrum, and Global Warming Potential of $(CF_3)_2CFCH$═CHF (HFO-1438ezy(E)). *The Journal of Physical Chemistry A*, *120*(33), 6618-6628. https://doi.org/10.1021/acs.jpca.6b06096

Papadimitriou, V. C., Spitieri, C. S., Papagiannakopoulos, P., Cazaunau, M., Lendar, M., Daële, V., & Mellouki, A. (2015). Atmospheric chemistry of $(CF3)_2C$=CH_2: OH radicals, Cl atoms and O_3 rate coefficients, oxidation end-products and IR spectra [10.1039/C5CP03840E]. *Physical Chemistry Chemical Physics*, *17*(38), 25607-25620. https://doi.org/10.1039/C5CP03840E

PubChem. (2022). *Trifluoracetic acid*. National Center for Biotechnology Information, U.S. National Library of Medicine. Retrieved January from https://pubchem.ncbi.nlm.nih.gov/compound/6422

RSC. (2022). *ChemSpider*. Royal Society of Chemistry. Retrieved May from http://www.chemspider.com/

Simmonds, P. G., Rigby, M., McCulloch, A., O'Doherty, S., Young, D., Mühle, J., Krummel, P. B., Steele, P., Fraser, P. J., Manning, A. J., Weiss, R. F., Salameh, P. K., Harth, C. M., Wang, R. H. J., & Prinn, R. G. (2017). Changing trends and emissions of hydrochlorofluorocarbons (HCFCs)

and their hydrofluorocarbon (HFCs) replacements. *Atmospheric Chemistry and Physics*, *17*(7), 4641-4655. https://doi.org/10.5194/acp-17-4641-2017

Smith, C., Z.R.J. Nicholls, K. Armour, W. Collins, P. Forster, M. Meinshausen, M.D. Palmer, M. Watanabe. (2021). The Earth's Energy Budget, Climate Feedbacks, and Climate Sensitivity Supplementary Material. In V. Masson-Delmotte, P. Zhai, A. Pirani, S.L. Connors, C. Péan, S. Berger, N. Caud, Y. Chen, L. Goldfarb, M.I. Gomis, M. Huang, K. Leitzell, E. Lonnoy, J.B.R. Matthews, T.K. Maycock, T. Waterfield, O. Yelekçi, R. Yu, B. Zhou (Ed.), *Climate Change 2021: The Physical Science Basis. Contribution of Working Group I to the Sixth Assessment Report of the Intergovernmental Panel on Climate Change*

Sulbaek Andersen, M. P., Hurley, M. D., & Wallington, T. J. (2009). Kinetics of the gas phase reactions of chlorine atoms and OH radicals with $CF_3CBr=CH_2$ and $CF_3CF_2CBr=CH_2$. *Chemical Physics Letters*, *482*(1), 20-23. https://doi.org/https://doi.org/10.1016/j.cplett.2009.09.056

Sulbaek Andersen, M. P., & Nielsen, O. J. (2022). Tropospheric photolysis of CF3CHO. *Atmospheric Environment*, *272*, 118935. https://doi.org/10.1016/j.atmosenv.2021.118935

Sulbaek Andersen, M. P., Nielsen, O. J., Hurley, M. D., & Wallington, T. J. (2012). Atmospheric chemistry of *t*-$CF_3CH=CHCl$: products and mechanisms of the gas-phase reactions with chlorine atoms and hydroxyl radicals [10.1039/C1CP22925G]. *Physical Chemistry Chemical Physics*, *14*(5), 1735-1748. https://doi.org/10.1039/C1CP22925G

Sulbaek Andersen, M. P., Nielsen, O. J., Karpichev, B., Wallington, T. J., & Sander, S. P. (2012). Atmospheric Chemistry of Isoflurane, Desflurane, and Sevoflurane: Kinetics and Mechanisms of Reactions with Chlorine Atoms and OH Radicals and Global Warming Potentials. *The Journal of Physical Chemistry A*, *116*(24), 5806-5820. https://doi.org/10.1021/jp2077598

Sulbaek Andersen, M. P., Nielsen, O. J., & Sherman, J. D. (2021). The Global Warming Potentials for Anesthetic Gas Sevoflurane Need Significant Corrections. *Environmental Science & Technology*, *55*(15), 10189-10191. https://doi.org/10.1021/acs.est.1c02573

Sulbaek Andersen, M. P., Schmidt, J. A., Volkova, A., & Wuebbles, D. J. (2018). A three-dimensional model of the atmospheric chemistry of E and Z-CF3CH=CHCl (HCFO-1233(zd) (E/Z)). *Atmospheric Environment*, *179*, 250-259. https://doi.org/10.1016/j.atmosenv.2018.02.018

Sulbaek Andersen, M. P., Sølling, T. I., Andersen, L. L., Volkova, A., Hovanessian, D., Britzman, C., Nielsen, O. J., & Wallington, T. J. (2018). Atmospheric chemistry of (Z)-$CF_3CH=CHCl$: products and mechanisms of the Cl atom, OH radical and O_3 reactions, and role of (E)–(Z) isomerization [10.1039/C8CP04903C]. *Physical Chemistry Chemical Physics*, *20*(44), 27949-27958. https://doi.org/10.1039/C8CP04903C

Sulbaek Andersen, M. P., Toft, A., Nielsen, O. J., Hurley, M. D., Wallington, T. J., Chishima, H., Tonokura, K., Mabury, S. A., Martin, J. W., & Ellis, D. A. (2006). Atmospheric chemistry of perfluorinated aldehyde hydrates (n-C_xF_{2x}+1CH$(OH)_2$,x= 1, 3, 4): Hydration, dehydration, and kinetics and mechanism of Cl atom and OH radical initiated oxidation. *The Journal of Physical Chemistry A*, *110*(32), 9854-9860. https://doi.org/10.1021/jp060404z

Taniguchi, N., Wallington, T. J., Hurley, M. D., Guschin, A. G., Molina, L. T., & Molina, M. J. (2003). Atmospheric Chemistry of $C_2F_5C(O)CF(CF_3)_2$: Photolysis and Reaction with Cl Atoms, OH Radicals, and Ozone. *The Journal of Physical Chemistry A*, *107*(15), 2674-2679. https://doi.org/10.1021/jp0220332

Tuazon, E. C., & Atkinson, R. (1993). Tropospheric transformation products of a series of hydrofluorocarbons and hydrochlorofluorocarbons. *Journal of Atmospheric Chemistry*, *17*(2), 179-199. https://doi.org/10.1007/BF00702825

Vollmer, M. K., Miller, B. R., Rigby, M., Reimann, S., Mühle, J., Krummel, P. B., O'Doherty, S., Kim, J., Rhee, T. S., Weiss, R. F., Fraser, P. J., Simmonds, P. G., Salameh, P. K., Harth, C. M., Wang, R. H. J., Steele, L. P., Young, D., Lunder, C. R., Hermansen, O., . . . Prinn, R. G. (2011). Atmospheric histories and global emissions of the anthropogenic hydrofluorocarbons HFC-365mfc, HFC-245fa, HFC-227ea, and HFC-236fa. *Journal of Geophysical Research: Atmospheres*, *116*(D8). https://doi.org/https://doi.org/10.1029/2010JD015309

Vollmer, M. K., Reimann, S., Hill, M., & Brunner, D. (2015). First observations of the fourth generation synthetic halocarbons HFC-1234yf, HFC-1234ze(E), and HCFC-1233zd(E) in the atmosphere. *Environmental Science & Technology*, *49*(5), 2703-2708. https://doi.org/10.1021/es505123x

Vollmer, M. K., Rhee, T. S., Rigby, M., Hofstetter, D., Hill, M., Schoenenberger, F., & Reimann, S. (2015). Modern inhalation anesthetics: Potent greenhouse gases in the global atmosphere. *Geophysical Research Letters*, *42*(5), 1606-1611. https://doi.org/https://doi.org/10.1002/2014GL062785

Vollmer, M. K., Rigby, M., Laube, J. C., Henne, S., Rhee, T. S., Gooch, L. J., Wenger, A., Young, D., Steele, L. P., Langenfelds, R. L., Brenninkmeijer, C. A. M., Wang, J.-L., Ou-Yang, C.-F., Wyss, S. A., Hill, M., Oram, D. E., Krummel, P. B., Schoenenberger, F., Zellweger, C., . . . Reimann, S. (2015). Abrupt reversal in emissions and atmospheric abundance of HCFC-133a (CF_3CH_2Cl). *Geophysical Research Letters*, *42*(20), 8702-8710. https://doi.org/https://doi.org/10.1002/2015GL065846

Wallington, T. J., Hurley, M. D., Fedotov, V., Morrell, C., & Hancock, G. (2002). Atmospheric Chemistry of $CF_3CH_2OCHF_2$ and $CF_3CHClOCHF_2$: Kinetics and Mechanisms of Reaction with Cl Atoms and OH Radicals and Atmospheric Fate of $CF_3C(O\bullet)HOCHF_2$ and $CF_3C(O\bullet)ClOCHF_2$ Radicals. *The Journal of Physical Chemistry A*, *106*(36), 8391-8398. https://doi.org/10.1021/jp020017z

Wallington, T. J., Hurley, M. D., Fracheboud, J. M., Orlando, J. J., Tyndall, G. S., Sehested, J., Møgelberg, T. E., & Nielsen, O. J. (1996). Role of Excited CF_3CFHO Radicals in the Atmospheric Chemistry of HFC-134a. *The Journal of Physical Chemistry*, *100*(46), 18116-18122. https://doi.org/10.1021/jp9624764

Wallington, T. J., Hurley, M. D., Xia, J., Wuebbles, D. J., Sillman, S., Ito, A., Penner, J. E., Ellis, D. A., Martin, J., Mabury, S. A., Nielsen, O. J., & Sulbaek Andersen, M. P. (2006). Formation of $C_7F_{15}COOH$ (PFOA) and Other Perfluorocarboxylic Acids during the Atmospheric Oxidation of 8:2 Fluorotelomer Alcohol. *Environmental Science & Technology*, *40*(3), 924-930. https://doi.org/10.1021/es051858x

Wallington, T. J., Liu, R., Dagaut, P., & Kurylo, M. J. (1988). The gas phase reactions of hydroxyl radicals with a series of aliphatic ethers over the temperature range 240–440 K [https://doi.org/10.1002/kin.550200106]. *International Journal of Chemical Kinetics*, *20*(1), 41-49. https://doi.org/https://doi.org/10.1002/kin.550200106

Wallington, T. J., Schneider, W. F., Worsnop, D. R., Nielsen, O. J., Sehested, J., Debruyn, W. J., & Shorter, J. A. (1994). The environmental impact of CFC replacements HFCs and HCFCs. *Environmental Science & Technology*, *28*(7), 320A-326A. https://doi.org/10.1021/es00056a714

Wang, Y., Niu, J., Zhang, L., & Shi, J. (2014). Toxicity assessment of perfluorinated carboxylic acids (PFCAs) towards the rotifer *Brachionus calyciflorus* [Research Support, Non-U.S. Gov't]. *The Science of the Total Environment*, *491-492*, 266-270. https://doi.org/10.1016/j.scitotenv.2014.02.028

WMO (2022). Scientific Assessment of Ozone Depletion: 2022. In P. A. Newman, & X. X. X (Eds.), *Global Ozone Research and Monitoring Project – Report No. XX* (pp. XXX). Geneva, Switzerland: World Meteorological Organization. *In press*.

Wuebbles, D. J., & Patten, K. O. (2009). Three-Dimensional Modeling of HCFC-123 in the Atmosphere: Assessing Its Potential Environmental Impacts and Rationale for Continued Use. *Environmental Science & Technology*, *43*(9), 3208-3213. https://doi.org/10.1021/es802308m

Zellner, R., Bednarek, G., Hoffmann, A., Kohlmann, J. P., Mörs, V., & Saathoff, H. (1994). Rate and mechanism of the atmospheric degradation of 2H-heptafluoropropane (HFC-227) [https://doi.org/10.1002/bbpc.19940980202]. *Berichte der Bunsengesellschaft für physikalische Chemie*, *98*(2), 141-146. https://doi.org/https://doi.org/10.1002/bbpc.19940980202

Zhang, N., Chen, L., Mizukado, J., Quan, H., & Suda, H. (2015). Rate constants for the gas-phase reactions of (*Z*)-CF_3CHCHF and (*E*)-CF_3CHCHF with OH radicals at 253–328K. *Chemical Physics Letters*, *621*, 78-84. https://doi.org/https://doi.org/10.1016/j.cplett.2014.12.044
